# Neuropsychological Assessments of Patients With Acquired Brain Injury: A Cluster Analysis Approach to Address Heterogeneity in Web-Based Cognitive Rehabilitation

**DOI:** 10.3389/fneur.2021.701946

**Published:** 2021-08-09

**Authors:** Alejandro García-Rudolph, Alberto García-Molina, Eloy Opisso, Josep María Tormos, Vince I. Madai, Dietmar Frey, Montserrat Bernabeu

**Affiliations:** ^1^Universitat Autònoma de Barcelona, Cerdanyola del Vallès, Spain; ^2^Fundació Institute d'Investigació en Ciències de la Salut Germans Trias i Pujol, Badalona, Spain; ^3^Institut Guttmann Hospital de Neurorehabilitacio, Badalona, Spain; ^4^CLAIM Charité Lab for AI in Medicine, Charité Universitätsmedizin Berlin, Berlin, Germany; ^5^QUEST Center for Transforming Biomedical Research, Berlin Institute of Health (BIH), Berlin, Germany; ^6^Faculty of Computing, Engineering and the Built Environment, School of Computing and Digital Technology, Birmingham City University, Birmingham, United Kingdom

**Keywords:** acquired brain injury, cluster analysis, cognitive rehabilitation, web-based platform, neuropsychological assessment, mixed-type data, functional independence

## Abstract

We aimed to (1) apply cluster analysis techniques to mixed-type data (numerical and categorical) from baseline neuropsychological standard and widely used assessments of patients with acquired brain injury (ABI) (2) apply state-of-the-art cluster validity indexes (CVI) to assess their internal validity (3) study their external validity considering relevant aspects of ABI rehabilitation such as functional independence measure (FIM) in activities of daily life assessment (4) characterize the identified profiles by using demographic and clinically relevant variables and (5) extend the external validation of the obtained clusters to all cognitive rehabilitation tasks executed by the participants in a web-based cognitive rehabilitation platform (GNPT). We analyzed 1,107 patients with ABI, 58.1% traumatic brain injury (TBI), 21.8% stroke and 20.1% other ABIs (e.g., brain tumors, anoxia, infections) that have undergone inpatient GNPT cognitive rehabilitation from September 2008 to January 2021. We applied the k-prototypes algorithm from the clustMixType R package. We optimized seven CVIs and applied bootstrap resampling to assess clusters stability (fpc R package). Clusters' *post hoc* comparisons were performed using the Wilcoxon ranked test, paired *t-*test or Chi-square test when appropriate. We identified a three-clusters optimal solution, with strong stability (>0.85) and structure (e.g., Silhouette > 0.60, Gamma > 0.83), characterized by distinctive level of performance in all neuropsychological tests, demographics, FIM, response to GNPT tasks and tests normative data (e.g., the 3 min cut-off in Trail Making Test-B). Cluster 1 was characterized by severe cognitive impairment (*N* = 254, 22.9%) the mean age was 47 years, 68.5% patients with TBI and 22% with stroke. Cluster 2 was characterized by mild cognitive impairment (*N* = 376, 33.9%) mean age 54 years, 53.5% patients with stroke and 27% other ABI. Cluster 3, moderate cognitive impairment (*N* = 477, 43.2%) mean age 33 years, 83% patients with TBI and 14% other ABI. Post hoc analysis on cognitive FIM supported a significant higher performance of Cluster 2 vs. Cluster 3 (*p* < 0.001), Cluster 2 vs. Cluster 1 (*p* < 0.001) and Cluster 3 vs. Cluster 1 (*p* < 0.001). All patients executed 286,798 GNPT tasks, with performance significantly higher in Cluster 2 and 3 vs. Cluster 1 (*p* < 0.001).

## Introduction

Cognitive rehabilitation is widely recognized as a standard element of rehabilitation services for patients with acquired brain injury (ABI) in several international clinical guidelines [e.g., the European Federation of Neurological Sciences (1) or the Brain Injury Special Interest Group of the American Congress of Rehabilitation Medicine (2)].

New strategies for providing cognitive rehabilitation programs are required and continuously being integrated into clinical practice (3). One of such strategies is the incorporation of web-based systems, with several of them having already contributed to optimizing cognitive interventions (4, 5). Due to the relative recent arrival of such services, best strategies for integrating them into everyday clinical practice are still in progress (6). Nevertheless, those aiming for personalization of the proposed rehabilitation activities to patients according to their specific needs, appear to be more effective (7). It has been previously reported that approaches that: (1) use a baseline cognitive evaluation founded on standardized neuropsychological tests to personalize the therapeutic interventions (2) offer immediate task-specific feedback to end-users and (3) dynamically adjust the rehabilitation scheme accordingly, are most effective (8).

The heterogeneity of ABI is extensively considered as one of the most significant barriers to finding effective therapeutic interventions (9). Identifying subgroups of patients who have distinguishable cognitive profiles that, in turn can assist in treatment planning and patient care, is crucial. Cluster analysis (CA) allows for the identification of homogeneous subgroups where cognitive heterogeneity is present, based on similarities in performance on baseline neuropsychological tests (10, 11).

The application of CA to baseline assessments of patients with ABI, still presents several limitations, despite the extensive production of research literature for over 25 years: (i) lack of internal validation using standardized cluster validity indices (CVI) (12) (ii) CA has been applied as isolated instances, barely integrated in the context of web-based cognitive rehabilitation (11) (iii) lack of external validation of the obtained clusters (e.g., considering patients' performance in activities of daily living) (iv) reduced samples (rarely larger than *n* = 500) (v) CA has been applied to baseline assessments of individual cognitive functions, therefore not allowing for a comprehensive description of patient's profiles (13) (vi) CA has been mostly applied to numerical variables (vii) CA methods have been traditionally implemented using commercial software packages as opposed to open access libraries (14, 15).

Therefore, in this work we aimed to (1) apply CA techniques to mixed data types (categorical and numerical variables) from baseline neuropsychological assessments of patients with ABI (2) apply seven different state-of-the-art CVIs for assessing clusters' internal validity to identify the optimal number of clusters considering different criteria (3) once an optimal number of clusters is selected, study their external validity, considering (i) the baseline assessments not used for creating the clusters, (ii) other clinical and demographic variables relevant to ABI rehabilitation (such as gender or time since injury to assessment) and (iii) motor and cognitive functional independence measure (FIM) in activities of daily living (4) characterize the identified profiles by using demographic and clinically relevant variables and (5) extend the external validation of the obtained clusters to all cognitive rehabilitation tasks executed by the participants included in the study along their whole rehabilitation process in a web-based cognitive rehabilitation platform.

Guttmann, NeuroPersonalTrainer^®^ (GNPT) (16) is the web-based cognitive rehabilitation platform used in the present study. At admission to cognitive rehabilitation using GNPT, every patient is assessed using a comprehensive standardized battery of neuropsychological tests (baseline assessment). The actual GNPT implementation integrates an automatic therapy planning functionality, the Intelligent Therapy Assistant (ITA) (17). The ITA provides therapists with a recommended schedule of cognitive tasks to be executed by each patient during a given period of time. Therapists can in turn manually modify the proposed schedule of tasks to their own preferences, according to their criteria (for example changing their order or modifying their execution parameters). In order to propose such schedule of tasks, the ITA takes as starting point a set of patient's cognitive profiles, obtained using CA from the baseline neuropsychological assessment (17).

Therefore, the main clinical implication of this work is to provide a validated (internally and externally) hands-on approach based on standardized neuropsychological baseline assessments and CA methods, implemented using open access software libraries. The proposed approach can thus be applied in the context of other web-based cognitive platforms that usually integrate an initial neuropsychological profiling of the patients to overcome heterogeneity. Extensions to similar web-based platforms (18) and to other populations with cognitive impairments may also build on this work.

## Materials and Methods

### Subject Selection

All patients were consecutively admitted to the rehabilitation unit of the ABI Department of Institut Guttmann, Barcelona Spain, between January 2008 and December 2020. Institut Guttmann is a specialized clinical center certified in quality of care and patient safety (Joint Commission International since 2005, consecutively recertified in 2009, 2012 and 2018) (19).

From an initial cohort of 2,312 patients with ABI, we enrolled only those who fulfilled the following inclusion criteria: (1) Traumatic brain injury (TBI), ischemic or hemorrhagic stroke or other ABI (brain tumor, anoxia,…) identified based on medical records relative to the acute phase of the intensive care unit (ICU) period; (2) age ≥18 years; and (3) first admission to neurorehabilitation unit. Exclusion criteria were: (1) the presence of aphasia; (2) the presence of a premorbid history of psychiatric disease or other severe disability (e.g., tetraplegia, paraplegia; and (3) time since injury to GNPT rehabilitation admission >365 days.

**Figure 1:**
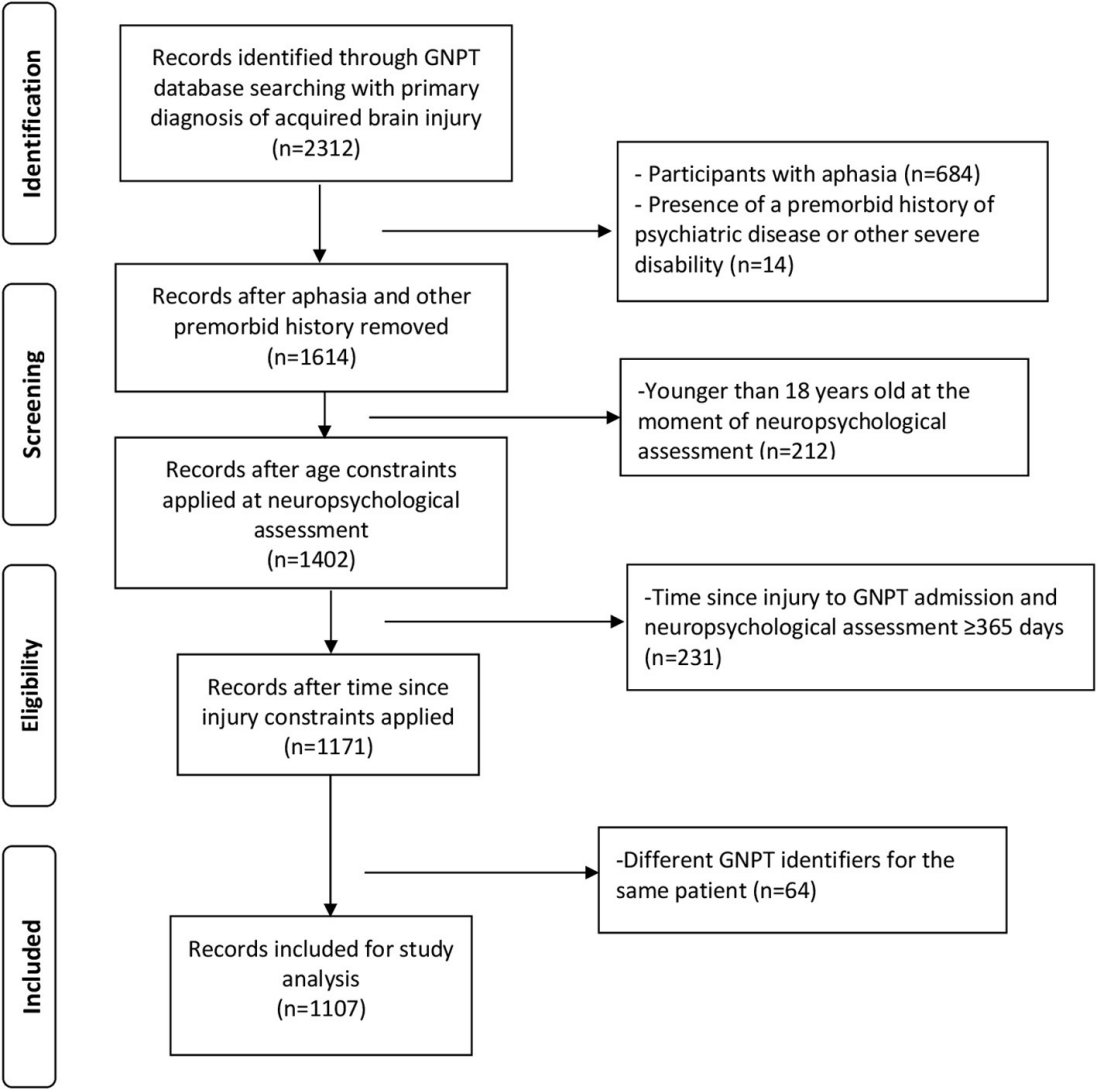
Participants' selection flowchart.

From the initial group, 684 patients with aphasia and 14 with other severe disability were removed. In the resulting 1,614 patients 212 were younger than 18 years old at the moment of neuropsychological assessment and 231 with more than 365 years since injury to neuropsychological assessment before starting GNPT. Finally, from the 1,171 resulting patients, 64 had more than one GNPT identifier for the same patient, therefore we kept only the first one of them, leaving the 1,107 patients included in this study (Figure 1).

### Procedure and Neuropsychological Measures at Admission

This was a retrospective observational study conforming to the STROBE Guidelines (“Strengthening the Reporting of Observational Studies in Epidemiology”) (20).

At admission to the rehabilitation unit of the ABI Department of Institut Guttmann, each patient is assigned a medical doctor, who coordinates the rehabilitation team (a nurse, a neuropsychologist, a physiotherapist, an occupational therapist, a social worker and a clinical psychologist based on the characteristics of the case). Therefore, admission neuropsychological assessments (as well as all clinical and demographic data analyzed in this study) are systematically recorded in the electronic health records of the hospital. Such standard neuropsychological assessments (summarized in Table 1), were performed by trained neuropsychologists with experience in neurological cognitive disorders, who were blind to any other result.

**Table 1:** Neuropsychological assessments at admission with the identifiers used for each of them along this study.

**Test**	**Identifier**	**Function/** **Subfunction**
Test Barcelona—Personal Orientation	T1	Orientation/Personal
Test Barcelona—Spatial Orientation	T2	Orientation/Spatial
Test Barcelona—Temporal Orientation	T3	Orientation/Temporal
WAIS-III—Direct digits	T4	Attention/Selective
TMT–A	T5	Attention/Selective
STROOP—words	T6	Attention/Selective
STROOP—color	T7	Attention/Selective
STROOP—words/colors	T8	Attention/Selective
WAIS III—Keys	T9	Attention/Selective
WAIS III—Cubes	T14	Executive functions/Planning
WAIS III—Inverse digits	T15	Memory/Working memory
WAIS III—Letters and numbers	T16	Executive functions/Sequencing
RAVLT immediate recall	T17	Memory/verbal
RAVLT deferred recall	T18	Memory/verbal
RAVLT recognition	T19	Memory/verbal
TMT–B	T20	Executive functions/Flexibility
WCST—Categories	T21	Executive functions/Categorization
WCST—Perseverative errors	T22	Executive functions/Flexibility
STROOP—Interference	T23	Executive functions/Inhibition

*WAIS III, Wechsler Adult Intelligence Scale 3^rd^ version; TMT, Trail Making Test; RAVLT, Rey Auditory Verbal Learning Test; WCST, Wisconsin Card Sorting Test*.

Orientation was assessed using 3 specific items of the Barcelona Test (21) i.e., personal (denoted throughout this work as T1), spatial (T2) and temporal (T3) (22).

Attention was assessed using the Direct Digits (T4) of the Wechsler Adult Intelligence Test–III (WAIS-III) (23), the Trail Making Test–part A (TMT A) (T5) (24), the Stroop test (25) Words (T6), Colors (T7) and Words/colors (T8) (26) and the Keys (T9) subtest of the WAIS III (27).

Working memory was assessed using the WAIS III–Inverse digits (T15) and verbal memory using the Rey Auditory Verbal Memory Test (RAVLT) (28), specifically RAVLT learning (T17), RAVLT free-recall memory (T18) and RAVLT recognition (T19) (29).

In relation to executive functions, planning was assessed using the WAIS III—Cubes (T14), sequencing was assessed with the WAIS III—Letters and numbers (T16), the Trail Making Test—part B (TMT B) for flexibility (T20) (30). Categorization was assessed using the Wisconsin Card Sorting Test (31) (WCST)—Categories (T21) and WCST—Perseverative errors (T22) (32) and inhibition using the STROOP—Interference (T23) (33).

The authors confirm that this study is compliant with the Helsinki Declaration of 1975, as revised in 2008 and it was approved by the Ethics Committee of Clinical Research of Institut Guttmann.

The participants were anonymized and non-identifiable. A specific written informed consent was not required for participants to be included in this study, nevertheless at admission participants provide written informed consent to be included in research studies addressed by the Institut Guttmann hospital.

### Web-Based Cognitive Rehabilitation

The GNPT (34) web-based cognitive rehabilitation platform used in this study is composed by a set of 149 different web-based cognitive rehabilitation tasks. There is not an established previous order in which patients should execute such tasks. Therefore every patient executes (eventually) a different subset of them in a different order during their rehabilitation process, taking between 2 and 6 months, distributed in 2 to 5 sessions a week. During each session the patient executes between 4 to 10 cognitive rehabilitation tasks, the total duration of one session ranges between 45 minutes and 1 hour. Each task mainly addresses one of the following functions: memory, executive functioning, attention, gnosias, calculus, orientation, language and social cognition. Immediately after each execution of a task, the patient gets a feedback on performance (ranging from 0 to 100, as the percentage of compliance), 0% being the lowest level of compliance and 100% the highest (15).

### Statistical Analysis

Statistical analyses were performed using R (version 3.5.31) (35) a value of *P* < 0.05 is considered statistically significant.

#### Data Pre-processing

Before running the CA methods, a pre-processing phase was performed involving all neuropsychological assessments presented in Table 1. We run Spearman correlation analysis, using the corrplot R package (36). We analyzed all pairs of correlations and removed highly correlated variables (*r* > 0.5 *P* < 0.05) keeping at least one variable of each cognitive function represented in Table 1 (orientation, attention, memory and executive functions) no pair of variables with a significant correlation coefficient larger than 0.5 was kept.

Pre-processing phase also included Z-normalization (37) of all the included numerical variables, using the scale () function of the base R package (38).

#### Cluster Analysis Method: k-Prototypes

For cluster analysis based on mixed-type data (i.e., data consisting of numerical and categorical variables), comparatively few clustering methods are available. One popular approach to deal with this kind of problems is an extension of the k-means algorithm (39), the so-called k-prototypes algorithm, in this work we applied the implementation provided in the clustMixType R package (40).

The algorithm iterates in a manner similar to the k-means algorithm where for the numeric variables the mean and for the categorical variables the mode, minimizes the total within cluster distance.

The steps of the implementation are based on the Huang's k-prototypes algorithm (41):

1. Initialization with random cluster prototypes.
2. For each observation do:
  - Assign observations to its closest prototype according to a distance metric.
  - Update cluster prototypes by cluster-specific means/modes for all variables.
3. As long as any observations have swapped their cluster assignment in 2 or the maximum number of iterations has not been reached: repeat from 2.

#### Optimal Number of Clusters: Internal Validation

It is further known that the selection of a suitable number of clusters k is particularly crucial in partitioning cluster procedures. Many implementations of cluster validation indices are not suitable for mixed-type data as recently reported (42).

We compared the internal validity of the k-prototypes algorithm for different number of clusters (k = 2..6) using 7 state-of-the-art cluster validation indices implemented in the clustMixType R package: Tau, Gamma, GPlus, McClain, PtBiseral, Silhouette, Sum of Squares (43).

#### Clusters Stability

To assess whether a cluster represents true structure is to see if the cluster holds up under plausible variations in the dataset. We used bootstrap resampling (44) from the fpc package (45) to generate such perturbations in the input data and evaluate how stable the obtained clusters were.

#### External Validation

In order to validate any cluster solution, it is important to compare the resulting clusters on variables that were not included in the original clustering process (46).

We included external variables found in previous related research (15) such as gender, age, age intervals, educational level or time since injury. Furthermore, ABI is a major cause of long-term ADL disability (47) therefore we included a standard measure of functional independence extensively used in population after ABI, the FIM (Functional Independence Measure). The FIM is typically reported as a total score or subdivided into a motor and a cognitive sub-score (48). External validation also included all the GNPT tasks executed by all participating patients during the period under study.

#### Post hoc Analyses

We conducted post *hoc* comparisons considering both the variables used and not used for building the clusters. We used the Wilcoxon ranked test, paired *t*-test or Chi-square test when appropriate. The Shapiro Wilk test was used to assess normality and the Levene test for homogeneity of variances.

## Results

### Demographic and Clinical Characteristics at Baseline

In Table 2, demographic and neuropsychological variables collected in all participating patients at study entry are reported, 58.1% were patients with TBI, 21.8% patients with stroke and 20.1% other acquired brain injuries. Severity of injury was reported for patients with TBI, mean reported GCS was 6.5 (3.4) [regarded as severe (49)] and for patients with stroke mean NIHSS was 10.8 (5.2) [moderately severe (50)].

**Table 2:** Demographics and clinical characteristics at admission.

**Variable**	***N*** = **1,107**
Sex, male, *n* (%)	793 (71.6%)
Age	43.5 (14.8)
Injury, *n* (%)	
TBI	643 (58.1%)
Stroke	241 (21.8%)
Hemorrhagic	121 (50.2%)
Ischemic	120 (49.8%)
Other ABI	223 (20.1%)
Tumor	73 (32.7%)
Anoxia	65 (29.1%)
Infection	21 (9.4%)
Iatrogenesis	15 (6.7%)
Other	49 (22.1%)
TBI severity (GCS)	6.5 (3.4)
Stroke severity (NIHSS)	10.8 (5.2)
Test Barcelona—Personal Orientation (T1)	6.5 (1.5)
Test Barcelona—Spatial Orientation (T2)	4.3 (1.2)
Test Barcelona—Temporal Orientation (T3)	20.3 (5.8)
WAIS-III—Direct digits (T4)	5.2 (1.6)
TMT–A (T5)	100.9 (84.4)
STROOP—words (T6)	70.1 (20.4)
STROOP—color (T7)	48.9 (14.8)
STROOP—words/colors (T8)	28.6 (12.4)
WAIS III—Keys (T9)	37.8 (17.3)
WAIS III—Cubes (T14)	24.2 (12.0)
WAIS III—Inverse digits (T15)	3.7 (1.0)
WAIS III—Letters and numbers (T16)	7.1 (2.9)
RAVLT immediate recall (T17)	31.6 (11.3)
RAVLT deferred recall (T18)	4.4 (3.8)
RAVLT recognition (T19)	7.7 (5.1)
TMT–B (T20)	225.2 (163.3)
WCST—Categories (T21)	2.7 (2.4)
WCST—Perseverative errors (T22)	41.4 (35.9)
STROOP—Interference (T23)	−0.4 (6.1)
Time since injury in days	93.5 (68.2)
Time since injury in days (ranges), n (%)	
0–45	270 (24.4%)
46–90	410 (37.0%)
91–180	303 (27.4%)
181–364	124 (11.2%)
Education, *n* (%)	
Primary	518 (46.8%)
Secondary	365 (33.0%)
University	224 (20.2%)

*TBI, Traumatic brain injury; ABI, Acquired brain injury; GCS, Glasgow Coma Scale; NIHSS, National institute of Health Stroke Scale; WAIS III, Wechsler Adult Intelligence Scale 3^rd^ version; TMT, Trail Making Test; RAVLT, Rey Auditory Verbal Learning Test; WCST, Wisconsin Card Sorting Test*.

### Preprocessing Phase: Correlation Analysis and z Normalization

Spearmans' correlations for all pairs of variables were performed as initial pre-processing phase. As detailed in Supplementary Table 1, a representative example is the Barcelona Test, orientation variables were found to be highly correlated: T1 and T2 (*r* = 0.56, *p* < 0.001); T2 and T3 (*r* = 0.63, *p* < 0.001), therefore only T3 was kept in the final set of variables, as shown in Supplementary Table 1 T3 is not correlated to any other variable. Similarly, T5 is highly correlated with T6 (*r* = −0.60, *p* < 0.001), T7 (*r* = −0.57, *p* < 0.001), T9 (*r* = −0.72, *p* < 0.001), T20 (*r* = 0.79, *p* < 0.001) therefore only T5 was kept.

The final set of variables was: T3, T5, T14, T15, T18, T22, age at the moment of neuropsychological assessment and type of injury (TBI, STROKE, OTHER ABI). After z-normalization of the numerical variables, these were used as input to the clustering k-prototypes algorithm.

### Cluster Analysis: Internal Validation

Table 3 summarizes the results obtained for all ICVs with the k number of clusters ranging from 2 to 6. A positive value (+) in the Criteria column indicates that the higher the maximum value of the index is used to indicate the optimal number of clusters. Similarly, a negative value (–) in the Criteria column indicates that the minimum value of the index is used to indicate the optimal number of clusters.

**Table 3:** ICV indexes obtained for the different number of clusters (*k* = 2..6).

**Validation index**	**2**	**3**	**4**	**5**	**6**	**Criteria**
Tau	0.5103	0.5823	0.4648	0.4908	0.4635	+
Gamma	0.9844	0.8338	0.7060	0.7181	0.7474	+
GPlus	0.0020	0.0405	0.0656	0.0472	0.0486	−
McClain	0.0755	0.1068	0.1279	0.1177	0.1205	−
PtBiseral	0.7826	0.3209	0.2954	0.2578	0.2262	+
Silhouette	0.8926	0.6063	0.4199	0.4359	0.3758	+
Sum of Squares	731,194	553,509	450,482	394,372	356,340	−

The solutions with *k* = 2 and 3 clusters clearly show optimal values for all ICVs (as highlighted in bold in Table 3), but *k* = 3 outperforms *k* = 2 in the *sum of squares* index. Therefore a 3 clusters solution is proposed in this work.

Supplementary Figures 1–7 show the graphical representation of each of the ICV indexes for *k* = 2..6. Solutions with higher *k* values were also tested, but did not improve the obtained performances.

### Cluster Analysis: k-Prototypes Results

Table 4 presents the *k*-prototypes results considering only the variables used for creating the clusters and the corresponding post *hoc* comparisons.

**Table 4:** Kproto results considering only the variables used for creating the clusters with post *hoc* analysis.

**Variable**	**Cluster 1 (***N*** = 254)**	**Cluster 2 (***N*** = 376)**	**Cluster 3 (***N*** = 477) (***N*** = 670)**	**Post ***hoc*** comparisons**	***p***
Age	47.6 (14.7)	54.1 (9.4)	32.9 (10.7)	Cluster 1 vs. Cluster 2	< 0.001
				Cluster 1 vs. Cluster 3	< 0.001
				Cluster 2 vs. Cluster 3	< 0.001
T3	12.1 (9.1)	22.3 (1.7)	22.0 (2.1)	Cluster 1 vs. Cluster 2	< 0.001
				Cluster 1 vs. Cluster 3	< 0.001
				Cluster 2 vs. Cluster 3	0.0048
T5	250.1 (80.8)	68.1 (42.9)	75.4 (48.6)	Cluster 1 vs. Cluster 2	< 0.001
				Cluster 1 vs. Cluster 3	< 0.001
				Cluster 2 vs. Cluster 3	0.01777
T14	13.3 (6.9)	25.9 (11.6)	24.9 (12.1)	Cluster 1 vs. Cluster 2	< 0.001
				Cluster 1 vs. Cluster 3	< 0.001
				Cluster 2 vs. Cluster 3	0.1965
T15	2.9 (0.7)	4.1 (0.9)	3.6 (0.9)	Cluster 1 vs. Cluster 2	< 0.001
				Cluster 1 vs. Cluster 3	< 0.001
				Cluster 2 vs. Cluster 3	< 0.001
T18	1.0 (1.8)	6.8 (3.6)	3.7 (3.2)	Cluster 1 vs. Cluster 2	< 0.001
				Cluster 1 vs. Cluster 3	< 0.001
				Cluster 2 vs. Cluster 3	< 0.001
T22	95.9 (13.6)	20.1 (14.8)	37.6 (32.2)	Cluster 1 vs. Cluster 2	< 0.001
				Cluster 1 vs. Cluster 3	< 0.001
				Cluster 2 vs. Cluster 3	< 0.001
Injury, ***n*** (%)					
TBI	174 (68.5%)	73 (19.4%)	396 (83.0%)		< 0.001
STROKE	24 (9.4%)	201 (53.5%)	16 (3.4%)		
OTHER ABI	56 (22.0%)	102 (27.1%)	65 (13.6%)		

### Stability

We analyzed perturbations on input data considering *B* = 100 resampling runs, obtaining stable results. Vector of obtained cluster stabilities for Cluster1, Cluster2 and Cluster3 = [.0.88, 0.87, 0.90].

### Cluster Analysis: External Validation

We performed twofold external validation: (1) by using demographic (age, gender, education level, age ranges), clinical variables (T1, T2, T4, T6, T7, T8, T9, T13, T16, T17, T19, T20, T21, T23, T25) and then by using the total FIM, cognitive FIM and motor FIM subtotals at admission and (2) considering all cognitive tasks executed by the patients in GNPT during the period under study. Table 5 presents Kproto results considering the variables presented in Table 2 but not used for creating the clusters.

**Table 5:** Kproto results considering the variables not used for creating the clusters.

**Variables**	**Cluster 1 (***N*** = 254)**	**Cluster 2 (***N*** = 376)**	**Cluster 3 (***N*** = 477) (***N*** = 670)**
Sex, male, ***N*** (%)	185 (72.8%)	251 (66.8%)	357 (74.8%)
Age range, ***N*** (%)			
17–30	46 (18.1%)	7 (1.9%)	228 (47.8%)
31–55	129 (50.8%)	202 (53.7%)	235 (49.3%)
≥56	79 (31.1%)	167 (44.4%)	14 (2.9%)
T1	5.0 (2.9)	6.9 (0.5)	6.8 (0.4)
T2	2.9 (2.0)	4.7 (0.6)	4.6 (0.7)
T4	3.6 (2.3)	5.8 (1.2)	5.4 (1.1)
T6	53.3 (24.1)	75.0 (17.9)	69.4 (19.8)
T7	36.7 (16.6)	52.1 (13.3)	48.8 (14.4)
T8	21.3 (12.9)	30.4 (11.0)	28.6 (12.9)
T9	22.4 (16.1)	41.6 (17.7)	37.5 (15.8)
T13	16.7 (3.8)	18.8 (2.4)	18.8 (2.3)
T16	5.0 (2.9)	8.0 (2.5)	6.7 (2.8)
T17	22.0 (8.7)	37.1 (10.2)	29.5 (10.3)
T19	2.7 (3.9)	10.4 (4.2)	7.3 (4.8)
T20	480.8 (95.8)	142.5 (81.8)	209.6 (147.9)
T21	0.2 (0.8)	3.7 (2.2)	2.7 (2.4)
T23	1.5 (3.2)	1.6 (2.3)	1.2 (4.9)
T25	162.3 (140.7)	84.8 (79.8)	85.1 (79.1)
TSO	101.2 (67.1)	90.7 (73.7)	91.7 (63.9)
TSO range ***n*** (%)			
0–45	39 (15.4%)	120 (31.9%)	111 (23.3%)
46–90	97 (38.2%)	125 (33.2%)	188 (39.4%)
91–180	91 (35.8%)	87 (23.1%)	125 (26.2%)
181–364	27 (10.6%)	44 (11.7%)	53 (11.1%)
studies			
Primary (6 years)	139 (54.7%)	162 (43.1%)	217 (45.5%)
Secondary (11 years)	66 (26.0%)	119 (31.6%)	180 (37.7%)
Tertiary (12 + years)	49 (19.3%)	95 (25.3%)	80 (16.8%)

*TSO, Time since onset to rehab assessment*.

As presented in Table 5, mean TMT-B (T20) values were 480.8 (95.8) for Cluster 1, 142.5 (81.8) for Cluster 2 and 209.6 (147.9) for Cluster 3. Therefore, Cluster 1 is remarkably lower but both Cluster 2 and 3 higher than the normative value for Spanish people with TBI. It is important to remark that the percentage of patients with TBI in each of the obtained clusters was 68.5% in Cluster 1, 19.4% in Cluster 2 and 83.0% in Cluster 3.

Table 6 presents the post *hoc* comparisons of Cluster 2 vs. Cluster 3 of the variables not used for creating the clusters.

**Table 6:** Post *hoc* comparisons of Cluster 2 and Cluster 3 of the variables not used for creating the clusters.

**Variables**	**Cluster 2 (***N*** = 376)**	**Cluster 3 (***N*** = 477) (***N*** = 670)**	***p***
T1	6.9 (0.5)	6.8 (0.4)	0.02073
T2	4.7 (0.6)	4.6 (0.7)	0.002147
T4	5.8 (1.2)	5.4 (1.1)	< 0.001
T6	75.0 (17.9)	69.4 (19.8)	0.0112
T7	52.1 (13.3)	48.8 (14.4)	0.02537
T8	30.4 (11.0)	28.6 (12.9)	0.1004
T9	41.6 (17.7)	37.5 (15.8)	0.0117
T13	18.8 (2.4)	18.8 (2.3)	0.8172
T16	8.0 (2.5)	6.7 (2.8)	< 0.001
T17	37.1 (10.2)	29.5 (10.3)	< 0.001
T19	10.4 (4.2)	7.3 (4.8)	< 0.001
T20	142.5 (81.8)	209.6 (147.9)	< 0.001
T21	3.7 (2.2)	2.7 (2.4)	< 0.001
T23	1.6 (2.3)	1.2 (4.9)	< 0.001
T25	84.8 (79.8)	85.1 (79.1)	0.367
TSO	90.7 (73.7)	91.7 (63.9)	0.07775

*TSO, Time since onset to rehab assessment*.

Table 7 presents the external validation using the total FIM, cognitive FIM and motor FIM for *N* = 947 patients, therefore FIM was available for 85% of the initial 1,107 patients. Post *hoc* analysis shows significant differences between all three clusters for cognitive FIM (details are presented in Supplementary Figure 8). When comparing motor and total FIM, significant differences were found between Cluster 1 vs Cluster 2, Cluster 1 vs Cluster 3, but not between Cluster 2 vs Cluster 3.

**Table 7:** FIM post *hoc* analysis.

**Variable**	**Cluster 1 (***N*** = 213)**	**Cluster 2 (***N*** = 329)**	**Cluster 3 (***N*** = 405) (***N*** = 670)**	**Post ***hoc*** comparisons**	***p***
Age	50.0 (13.7)	54.4 (9.3)	33.0 (10.6)	Cluster 1 vs. Cluster 2	< 0.001
				Cluster 1 vs. Cluster 3	< 0.001
				Cluster 2 vs. Cluster 3	< 0.001
TOTAL FIM	69.9 (33.6)	79.7 (29.7)	79.9 (33.3)	Cluster 1 vs. Cluster 2	< 0.001
				Cluster 1 vs. Cluster 3	< 0.001
				Cluster 2 vs. Cluster 3	0.621
COGNITIVE FIM	21.7 (8.5)	26.6 (7.6)	24.4 (8.2)	Cluster 1 vs. Cluster 2	< 0.001
				Cluster 1 vs. Cluster 3	< 0.001
				Cluster 2 vs. Cluster 3	< 0.001
MOTOR FIM	48.2 (27.2)	53.1 (24.7)	55.5 (27.0)	Cluster 1 vs. Cluster 2	0.035
				Cluster 1 vs. Cluster 3	0.003
				Cluster 2 vs. Cluster 3	0.156

*FIM, Functional Independence Measure*.

Cluster 2 and Cluster 3 were significantly different in cognitive FIM, median and IQR values were 29.0 (22.0−32.0) and 26.0 (20.0–31.0) respectively, with mean values 26.6 (7.6) and 24.4 (8.2) (*p* < 0.001) as presented in Table 7. Cognitive FIM cut-off value for home discharge was previously reported as 23.5 points, with a sensitivity of 73.7% and a specificity of 80.6% (51). Therefore, the mean reported value for patients in Cluster 3 was less than one point above the cut-off for home discharge, meanwhile participants in Cluster 2 were more than three points above it.

As presented in Table 6 significant differences were found between Cluster 2 and Cluster 3 in post *hoc* comparisons of TMT-B (T20). Differences with Cluster 1 were not reported in Table 6 because they were significantly lower in all tests, also as confirmed in the cognitive, motor and total FIM in Table 7.

Table 8 presents Kproto results considering the total number of GNPT tasks executed by the 1,107 participants. The total number of executed GNPT tasks were 286,798 with 66,933 executed by patients from Cluster 1, 81,031 executed by patients from Cluster 2 and 138,834 executed by patients from Cluster 3.

**Table 8:** GNPT tasks executions by cluster.

**Variable**	**Cluster 1 (***N*** = 66,933)**	**Cluster 2 (***N*** = 81,031)**	**Cluster 3 (***N*** = 138,834) (***N*** = 670)**
result	65.1 (33.8)	65.5 (35.4)	68.7 (33.7)
	76.4 (41.1–95.0)	80.0 (40.0–97.0)	82.0 (50.0–97.2)
Memory	26,177 (39.1%)	36,117 (44.6%)	54,944 (39.6%)
Executive	15,359 (22.9%)	23,101 (28.5%)	38,350 (27.6%)
ATTENTION	15,742 (23.5%)	14,421 (17.8%)	29,417 (21.2%)
Language	5,333 (8.0%)	2,494 (3.1%)	6,597 (4.8%)
Calculus	2,288 (3.4%)	2,048 (2.5%)	3,184 (2.3%)
Gnosias	1,047 (1.6%)	1,614 (2.0%)	3,293 (2.4%)
Orientation	628 (0.9%)	782 (1.0%)	1,796 (1.3%)
Socialization	359 (0.5%)	454 (0.6%)	1,253 (0.9%)

**Figure 2:**
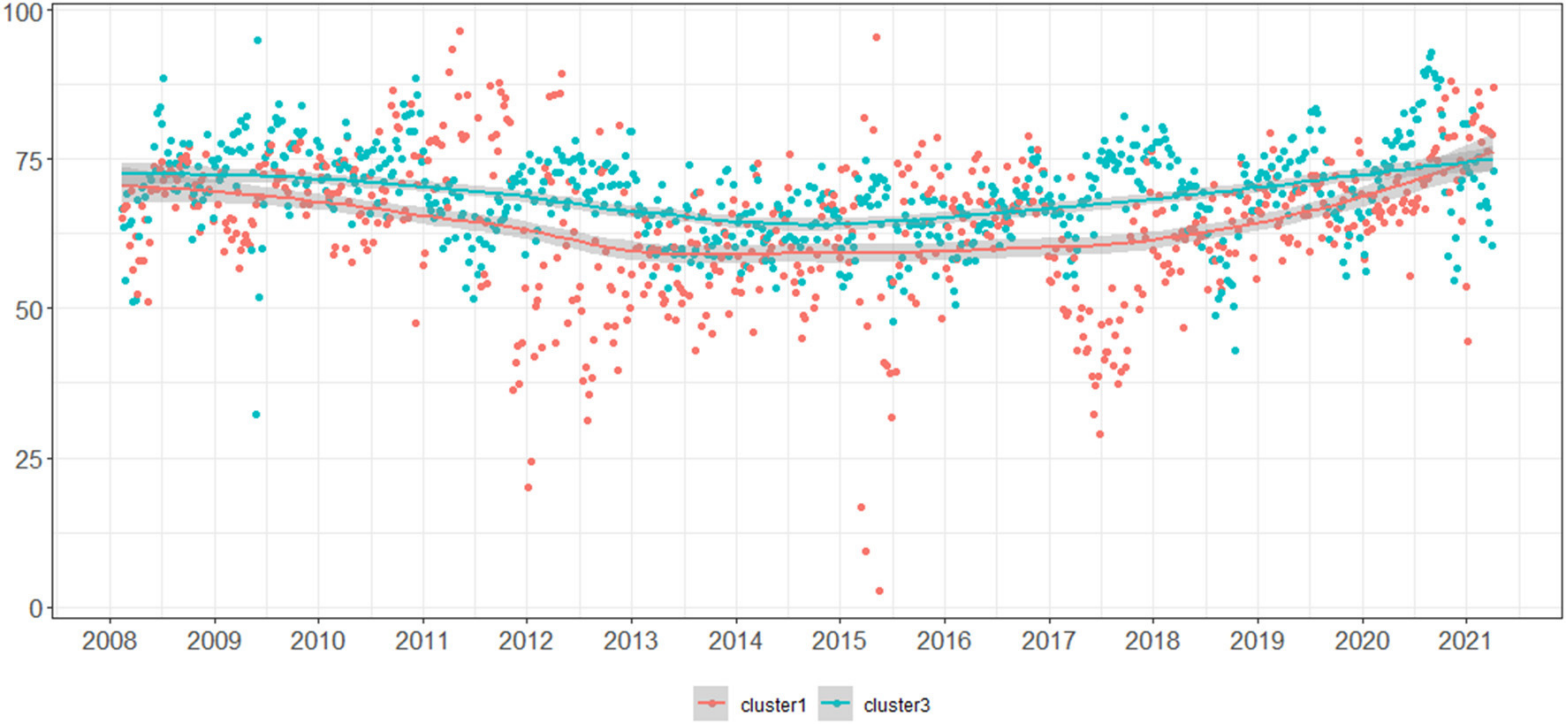
Mean weekly results obtained by all participants grouped in Cluster 1 (red) and Cluster 3 (blue).

Figure 2 plots the mean weekly results obtained by all participants grouped in Cluster 1 (red) and Cluster 3 (blue), showing a consistent higher performance by participants from Cluster 3 during the whole period under study.

## Discussion

In this work we extended the application of CA techniques to mixed data variables from baseline neuropsychological assessments of patients with ABI. We applied several state-of-the-art CVI involving such new set of categorical variables to assess clusters' internal validity. We studied the external validity of the obtained clusters considering relevant aspects of ABI rehabilitation such functional independence in activities of daily life and characterized the identified profiles by using demographic and clinically relevant variables. Finally, clusters' characterization was confirmed using all cognitive rehabilitation tasks executed by the patients included in the study along their whole rehabilitation process in a web-based GNPT cognitive rehabilitation platform.

CA has been previously applied to the neuropsychological assessments presented in Table 1, the number of identified clusters varied from 3 to 6, to our best knowledge most of them addressing patients with TBI, very few of them included patients with stroke or with other ABI. Besides, very few of them involving a sample larger than 300, none of them presented the CA as a component of a web-based cognitive rehabilitation service, very few of them considered more than one CVIs, none of them performed external validation using activities of daily living, neither cognitive rehabilitation tasks.

For example, Thaler et al. (52) used CA to address heterogeneity in TMT A and B in participants with mild TBI (N=78). Three clusters were identified, two of them characterized by both TMT scores and the third with a single performance pattern characterized by lower scores on TMT B. Clusters did not differ on demographic or other clinical variables.

Harman-Smith et al. (53) analyzed all subtests of the WAIS-III from patients with mild, moderate, and severe TBI (n=220). They produced two solutions, one with 4 and the other with 6 clusters. According to the authors the latter better captures subtle variations in cognitive functioning. The 6 clusters differed in the levels and profiles of cognitive performance, self-reported recovery, education and injury severity.

Sherer et al. (54) applied CA to an Australian cohort of persons with TBI (*N* = 170) using the Wechsler Letter-Number Sequencing and Coding, the Rey Auditory Verbal Learning Test and the Trail Making Test. They identified a 5-clusters solution that largely replicated another cohort of persons with TBI previously presented by Sherer et al. (55).

Zimmermann et al. (56) analyzed a Brazilian cohort of outpatients with mild and moderate/ severe TBI (N=84) using the TMT, the Modified Wisconsin Card Sorting Test−48 cards, Verbal fluency measured using the Montreal Communication Assessment and Auditory oral word span in sentences. Three clusters emerged and were characterized by deficits in: (1) inhibition, flexibility and focused attention; (2) inhibition, flexibility, working memory and focused attention; and (3) no expressive executive deficits. Clusters did not differ in clinical or demographical variables.

Melendez-Moral and colleagues (57) applied CA to address heterogeneity in mild cognitive impairment (*N* = 30) using some items of the Test Barcelona, the Wechsler Memory Scale III and the TAVEC (Test de Aprendizaje Verbal España-Complutense). Three clusters emerged. Cluster 1 corresponded to the participants with low scores both in memory tests and in other cognitive domains, Cluster 2 included those subjects who showed low scores in memory tests, and Cluster 3 included those who did not show low scores in none of the evaluated tests.

In relation to patients with stroke, CA has been mostly applied to identify communication patterns, in patients with aphasia, therefore using tests not included in Table 1. For example, Ferré and colleagues (58) analyzed right brain damaged individuals (*N* = 71) from 3 nationalities (Canadians, Brazilians, Argentinians). CA led to five distinct clinical profiles of communication impairment.

Similarly, Akinina et al. (59) recently applied partitioning clustering to explore patterns of impairment in verb and sentence processing in a sample (*N* = 54) of people with aphasia. The analysis yielded a two clusters solution. The main difference between them was the severity of impairment, both at the single verb and at the sentence level, in production and in comprehension.

In relation to patients with other acquired brain injuries, to the best of our knowledge, previous CA research has not been applied using any of the neuropsychological tests presented in Table 1. CA applications addressed for example brain tumors segmentation of MRI images (60, 61), monitoring glioma heterogeneity during tumor growth (62) or region of interest analysis based on Mass Spectrometry Imaging (63).

As shown in Tables 4–8, in our case CA yielded to 3 clearly different clusters characterized by level of performance in neuropsychological tests, demographics, level of independence in ADLs as measured using the FIM and response to GNPT tasks:

Cluster 1 (*N* = 254. 22.9%) the mean age of participants in Cluster 1 at the moment of neuropsychological assessment was 47 years, 72.8% were males, 68.5% patients with TBI and 22% with stroke, 80.7% with <12 years of education. Cluster 2 (*N* = 376, 33.9%) mean age 54 years, 66.8% males, 53.5% patients with stroke and 27% other acquired brain injury, 74.7% with <12 years of education. Cluster 3 (*N* = 477, 43.2%) mean age 33 years, 74.8% males, 83% patients with traumatic brain injury and 14% other acquired brain injury, 83.2% with <12 years of education.

The FIM is currently considered as the most widely used measure to describe the degree of impairment in activities of daily living in clinical practice (64). Based on Rasch analysis FIM motor scores were categorized in previous research (65) into three levels: good, fair, and poor outcomes. A “good” outcome was defined as a patient achieving a FIM motor score of 65 or above. With a score of 65, patients usually require either supervision or minimal assistance with mobility and self-care, indicating that the patient' physical care requirements for daily activities are minimal. Scores above 46 indicate some improvement (“fair” outcome) and scores under 46 indicate a large physical burden of care (“poor” outcome). As shown in Table 7, mean motor FIM for patients in Cluster 1 was 48.2(27.2), motor FIM boxplots are presented in Supplementary Figure 8, median and IQR values were 44.0 (22.0–74.0) suggesting a large burden of care (poor outcome). Meanwhile Cluster 2 and 3 median and IQR values were 55.0 (31.0–76.0) and 59.0 (30.0–80.0) respectively, indicating fair outcome, close to minimal physical care requirements for daily activities. Table 7 shows no significant differences between Cluster 2 vs Cluster 3 (*p* = 0.621) as also detailed in Supplementary Figure 8.

In relation to level of performance in neuropsychological tests, Tables 4–6 support the differences observed in the FIM. According to different authors (30), TMT is one of the most widely used instruments in neuropsychological assessment. It has been used to test speed of processing, sequence alternation, cognitive flexibility, visual search, motor performance, and executive functioning (30). Besides, a positive linear relation between brain-injury severity and TMT performance has also been previously reported (66). Nevertheless, normative TMT values for Spanish population with ABI are scarce. According to a set of normative values (30) specifically developed for Spanish healthy middle-aged group (25–54 years; education: 0–12 years) mean TMT-A was 33.04 (7.89) and mean TMT-B was 71.5 (31.07). For Spanish population with TBI, mean age 35.26(12.88), education: 0–11 years, mean TMT-A was 82.76(52) and mean TMT-B was 218 [155]. Therefore, as presented in Table 4, mean TMT-A (T5) values were 68.1 (42.9) and 75.4 (48.6) for Cluster 2 and Cluster 3 respectively, in both cases lower than normative values for Spanish population with TBI. Meanwhile the mean value reported in Table 4 for T5 in Cluster 1 was 250.1 (80.8).

An important difference when comparing Cluster 2 and Cluster 3 in relation to TMT-B can be related to the 180 s cut-off value used (along with several other tests) for assessing fitness to drive. It is important to remark that car driving is a complex task that requires the successful integration of perceptual, physical, cognitive, and emotional systems (67), beyond the obtained score in TMT-B.

A systematic review of the evidence for TMT-B cut-off scores in assessing fitness-to-drive (68) concluded that there is informed support for the 180 s TMT-B cut-off. In our case, this cut-off value sets a clear difference between Cluster 2 and Cluster 3. The mean TMT-B score in Cluster 2 of 142.5 (81.8) with median and IQR 122.0 (92.2–170.0) was below the 180.0 s cut-off. Meanwhile for Cluster 3 the mean TMT-B score of 209.6 (147.9) with median and IQR 157.0 (107.0–255.5) was above the 180.0 s cut-off.

We are not suggesting that patients in Cluster 2 would be able to successfully return to drive, neither that patients in Cluster 3 would not, we are mentioning the 180 s cut-off value with the only purpose of remarking the differences between Cluster 2 and Cluster 3 in a relevant test such as TMT-B.

The level of interest in computerized cognitive training is growing more rapidly than other areas of rehabilitation aimed at healthy aging, possibly due to the increasing evidence of efficacy, sophistication of delivery systems, and accessibility of these systems across different platforms. The scientific community interest is also shown by the increasing number of related PubMed publications (69).

Recently reported (70) advantages of computerized cognitive programs include: (1) their programed self-sufficiency giving feedback, adjusting task difficulty and changing tasks on a customized basis according to an individual's performance; (2) their easy access on home computers and hand held devices; (3) their cost-effectiveness compared with paid-person support; and (4) most individuals' familiarity with—and enjoyment using—low technology computers and handheld devices.

A recent systematic review (71) including 28 studies of the use of computerized treatment as a rehabilitation tool for attention and executive function in adults (aged 18 years or older) who suffered TBI, stroke or other acquired brain injuries. In 23 studies, significant improvements in attention and executive function subsequent to computerized treatment were reported; in the remaining 5, promising trends were observed.

In terms of post-stroke cognitive improvement, the effectiveness of computerized cognitive training in some specific aspects of cognition, such as memory and executive functions, has been suggested in recent systematic reviews and clinical guidelines (72–74).

A systematic review and meta-analysis (75) based on randomized controlled trials from the last 10 years (2010–2020) was recently published to identify the effect of computer-based training compared to routine methods on post-stroke cognitive rehabilitation. Ten out of 201 studies were included in the systematic review, with a total of 600 stroke survivors. The authors did not obtain supporting evidence to identify the superiority of computer-based cognitive training for post-stroke cognitive impairment recovery compared to traditional interventions. However, they did not find any evidence for its side effects on cognitive rehabilitation. In addition, the advantages of convenience and time savings [e.g., (76)] with computer-based cognitive training cannot be neglected. The authors recommend to conduct more high-quality studies focusing on different illness phases and various types of intervention software in order to improve the meta-analysis and to explore the influence of computer-based cognitive rehabilitation by subgroup analysis.

In this context, our results support the execution of subgroup analysis using a set of publicly available R libraries (R-3.5.1) within a web-based cognitive platform (the GNPT) and extend the application of CA techniques to mixed data variables from baseline neuropsychological assessments of patients with ABI. Such techniques and R implementations can be used within other state-of-the-art computer-based cognitive rehabilitation platforms for subgroup analysis involving mixed data variables.

Furthermore, as presented in the Introduction section of this work, the Intelligent Therapy Assistant (ITA) (17) is already integrated in the GNPT platform. The ITA provides therapists with a recommended schedule of cognitive tasks to be executed by each patient during a given period of time. In order to propose such schedule of tasks, the ITA takes as starting point a set of patient's cognitive profiles, obtained using CA from the baseline neuropsychological assessment (17). Our results provide an alternative (straightforward and sound) initial step for the ITA, leaving room for a future comparative study involving the actual CA approach with the results of this work.

## Limitations of This Study

Several limitations to our study need to be remarked. First, we conducted a single-center study; an advantage of this is that data were reported by clinicians, with specific training in the applied neuropsychological rehabilitation procedures, and all patients were managed under the same ABI rehabilitation protocols. Nevertheless, the GNPT platform is already integrated into the clinical practice of several centers specialized in ABI care. Their patients were not included in this analysis, leaving space for future work. An important aspect to address in such future multicenter work, would be in relation to the external validation assessments (for example the FIM used in this study) which should be common to all participating centers.

Second, the health area studied belongs mainly to the Catalan Health Service, (with 75.5% of the 1,107 included patients from Catalonia and 24,5% from the rest of Spain). Furthermore, considering only participants from Catalonia, 79.5% of them were from Barcelona, 10.1% from Girona, 5.4% from Lleida and 5.1% from Tarragona). Therefore, from the total 1,107 included patients 60.1% were from Barcelona, that may be prone to selection bias. Nevertheless, we included standardized and widely applied assessments tools, extensively applied in related clinical centers.

Third, our analysis lacked computerized tomography or magnetic resonance imaging examinations that describe the presence of contusion, hematoma, hemorrhage, ischemia, or other signs of parenchymal lesion on frontal, temporal, parietal, occipital, and cerebellar lobes or diffuse axonal injury.

Fourth, k-prototypes implements a partitional clustering strategy, the most studied research theme in mixed data clustering as recently reported (42). Nevertheless, other available approaches such as hierarchical or model-based, can be considered. In this work we included as Supplementary Material_II the application of a popular hierarchical strategy for mixed data types, the Partitioning Around Medoids (PAM) (77) with Gower's dissimilarity, as implemented in the functions daisy () of the R package cluster (78) and gowdis () of the fd R package (79). As shown in Supplementary Material_II, the categorical variables seem to mask the other variables. For example, when including the injury categorical variable, the optimal solution yields to three clusters with all patients with TBI in one cluster (and only patients with TBI), all patients with stroke (and only patients with stroke) in the second cluster and all patients with other ABI (and only patients with other ABI) in the third cluster. Therefore, this approach requires further analysis leaving room for future work.

Fifth, our analysis did not include indicators of mental health or other comorbidities. Still, other medical comorbidities may begin months or years following injury in comparison to uninjured control groups. For example, previous studies have reported that individuals with TBI have more than twice the rates of pain, growth hormone deficiency, insomnia, fatigue, new-onset stroke, urinary incontinence, and epilepsy (80). Therefore, we aim to include comorbidity analysis in future research studies.

Sixth, a final point which is important to clarify is in relation to the actual tests used in this study. WAIS-III, TMT, RAVLT, WCST, STROOP or Barcelona Test date back over more than 30 years and could be regarded as outdated. Nevertheless, cluster analysis has been extensively applied using such tests as input in recent publications (52–63) and those tests are widely used as standard tools for neuropsychological evaluations in adults nowadays as recently reported (81).

## Conclusions

CA techniques implemented using a set of publicly available R libraries were applied in this work to mixed (numerical and categorical) variables from baseline neuropsychological assessments of patients with ABI, allowing the identification of three clinically sound and meaningful patients' profiles. The application of several state-of-the-art indexes confirmed the clusters' strong internal validity and stability. The external validity was also confirmed, considering a relevant aspect of ABI rehabilitation such as functional independence in activities of daily life and using all cognitive rehabilitation tasks executed by the patients included in the study along their whole rehabilitation process, in a web-based cognitive rehabilitation platform. The applied CA techniques and R implementations could eventually be used within other state-of-the-art computer-based cognitive rehabilitation tools for subgroup analysis involving mixed data variables, given the growing level of interest in computerized cognitive rehabilitation treatments, increasingly integrated into clinical practice.

## Data Availability Statement

The datasets generated for this study will be made available upon reasonable request to the corresponding author of the article.

## Ethics Statement

A specific written informed consent was not required for participants to be included in this study, in accordance with the local legislation and institutional requirements. Nevertheless at admission participants provide written informed consent to be included in research studies addressed by the Institut Guttmann hospital. The authors confirm that this study is compliant with the Helsinki Declaration of 1975, as revised in 2008 and it was approved by the Ethics Committee of Clinical Research of Institut Guttmann.

## Author Contributions

AG-R pre-processed the data, conducted data analysis, prepared the figures, drafted the initial manuscript, and revised the manuscript. AG-M designed the study, pre-processed the data, interpreted the data, and revised the manuscript. JMT designed the study, revised the manuscript, and interpreted the data. EO designed the study, conducted data analysis, interpreted the data, and revised the manuscript. DF and VIM acquired funding for the project leading to this publication and revised the manuscript. MB interpreted the data and revised the manuscript. All authors have contributed to manuscript revision, read, and approved the submitted version.

## Conflict of Interest

AG-R, AG-M, JMT, EO and MB work at Institut Guttmann, Hospital de Neurorehabilitació, proprietary of the Guttmann, NeuroPersonalTrainer^®^ platform. VIM reported receiving personal fees from ai4medicine outside the submitted work. There is no connection, commercial exploitation, transfer or association between the projects of ai4medicine and the results presented in this work. The remaining author declares that the research was conducted in the absence of any commercial or financial relationships that could be construed as a potential conflict of interest.

## Publisher's Note

All claims expressed in this article are solely those of the authors and do not necessarily represent those of their affiliated organizations, or those of the publisher, the editors and the reviewers. Any product that may be evaluated in this article, or claim that may be made by its manufacturer, is not guaranteed or endorsed by the publisher.
