# Supplementary material for: Neuropsychological Assessments of Patients With Acquired Brain Injury: A Cluster Analysis Approach to Address Heterogeneity in Web-Based Cognitive Rehabilitation
Source: Front Neurol. 2021 Aug 9;12:701946. doi: 10.3389/fneur.2021.701946 (PMC8380987; doi:10.3389/fneur.2021.701946)
Supplement: Supplementary file 1 [file Data_Sheet_1.PDF]

frontiers

Supplementary Material

Supplementary Table 1. Pearsons' correlations for all Neuropsychological assessments at admission with the identifiers (introduced in Table 1) used for each of them along this study

T1	**0.56**	0.49	0.31	-0.39	0.16	0.17	0.23	0.22	0.14	0.14	0.19	0.22	0.39	0.38	-0.45	0.41	-0.50	-0.06
	T2	**0.63**	0.31	-0.44	0.21	0.25	0.25	0.26	0.19	0.20	0.25	0.39	0.48	0.48	-0.50	0.47	-0.50	0.05
		T3	0.27	-0.41	0.21	0.24	0.23	0.27	0.23	0.21	0.27	0.37	0.45	0.43	-0.43	0.39	-0.43	0.04
			T4	-0.36	0.38	0.39	0.37	0.27	0.26	**0.55**	0.46	0.32	0.28	0.30	-0.45	0.40	-0.52	0.01
				T5	**-0.60**	**-0.57**	-0.52	**-0.72**	-0.45	-0.35	-0.44	-0.41	-0.44	-0.45	**0.79**	0.53	0.62	-0.18
					T6	**0.75**	**0.60**	0.58	0.38	0.40	0.48	0.40	0.29	0.29	-0.50	0.32	-0.29	0.12
						T7	**0.74**	0.56	0.38	0.41	0.50	0.45	0.38	0.35	-0.52	0.31	-0.30	0.19
							T8	**0.60**	0.43	0.40	0.49	0.45	0.34	0.29	-0.52	0.34	-0.40	0.73
								T9	**0.52**	0.39	0.47	0.49	0.41	0.38	**-0.64**	0.32	-0.38	0.21
									T14	0.34	0.46	0.30	0.22	0.22	-0.39	0.47	-0.51	0.19
										T15	**0.58**	0.37	0.25	0.25	-0.35	0.23	-0.32	0.05
											T16	0.47	0.37	0.38	-0.49	0.37	-0.36	0.19
												T17	**0.80**	0.69	-0.40	0.32	-0.31	0.08
													T18	**0.78**	-0.49	0.45	-0.47	0.01
														T19	-0.45	0.43	-0.46	0.03
															T20	-0.56	**0.65**	-0.12
																T21	-0.93	0.16
																	T22	-0.16
																		T23

NOTE: Significant correlations $r \geq 0.50$ ($p < 0.001$) are highlighted in bold.

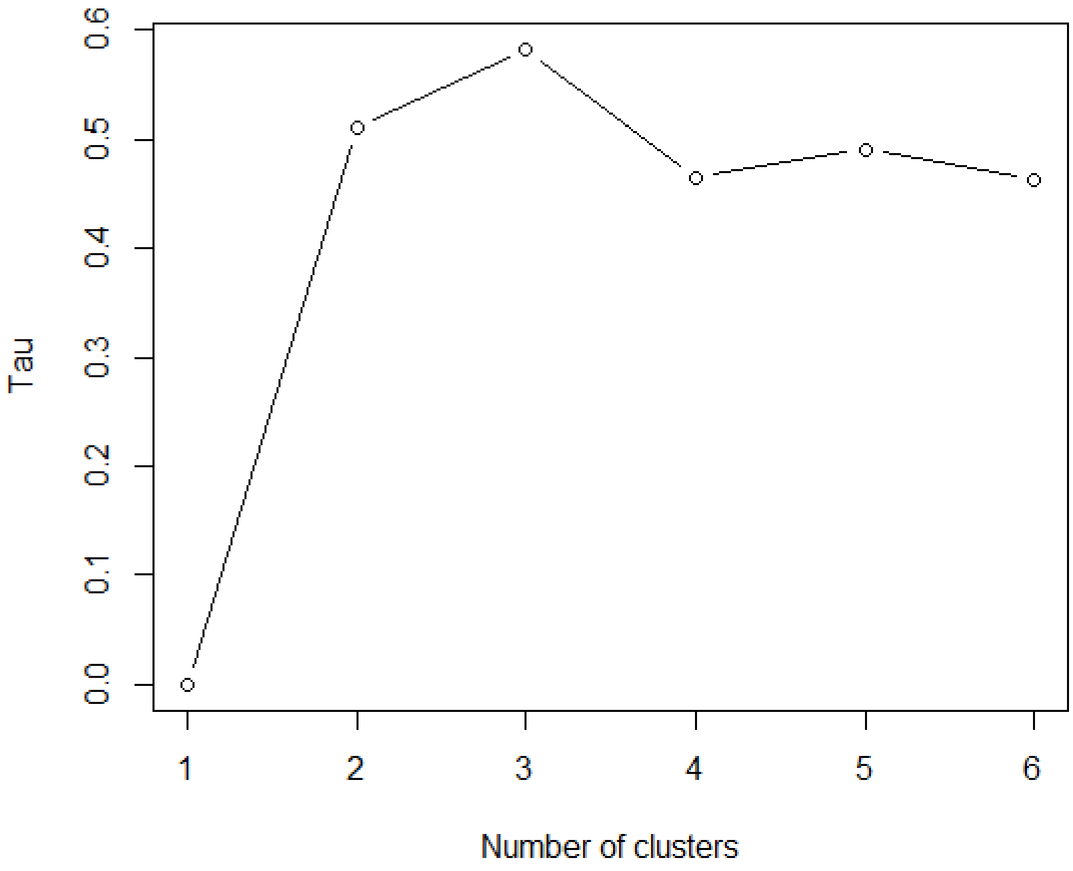

Supplementary Figure 1. Tau plot for k= 1..6 number of clusters

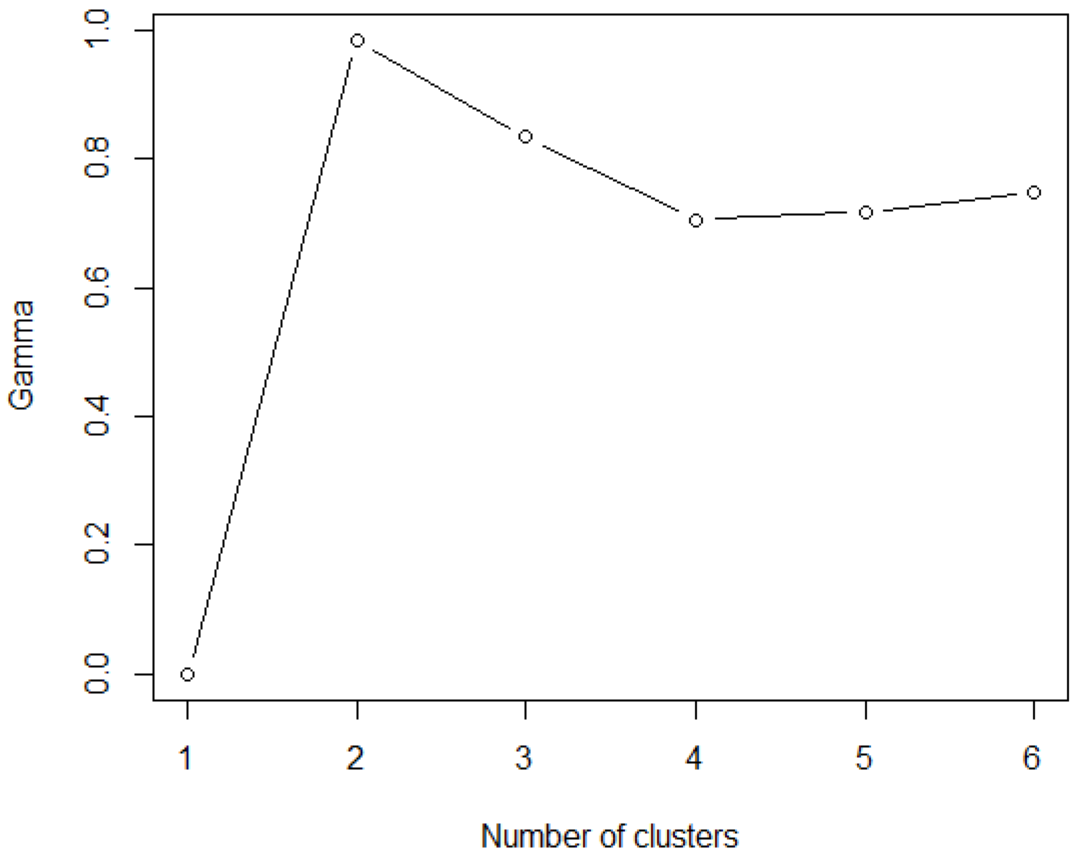

Supplementary Figure 2. Gamma plot for k= 1..6 number of clusters

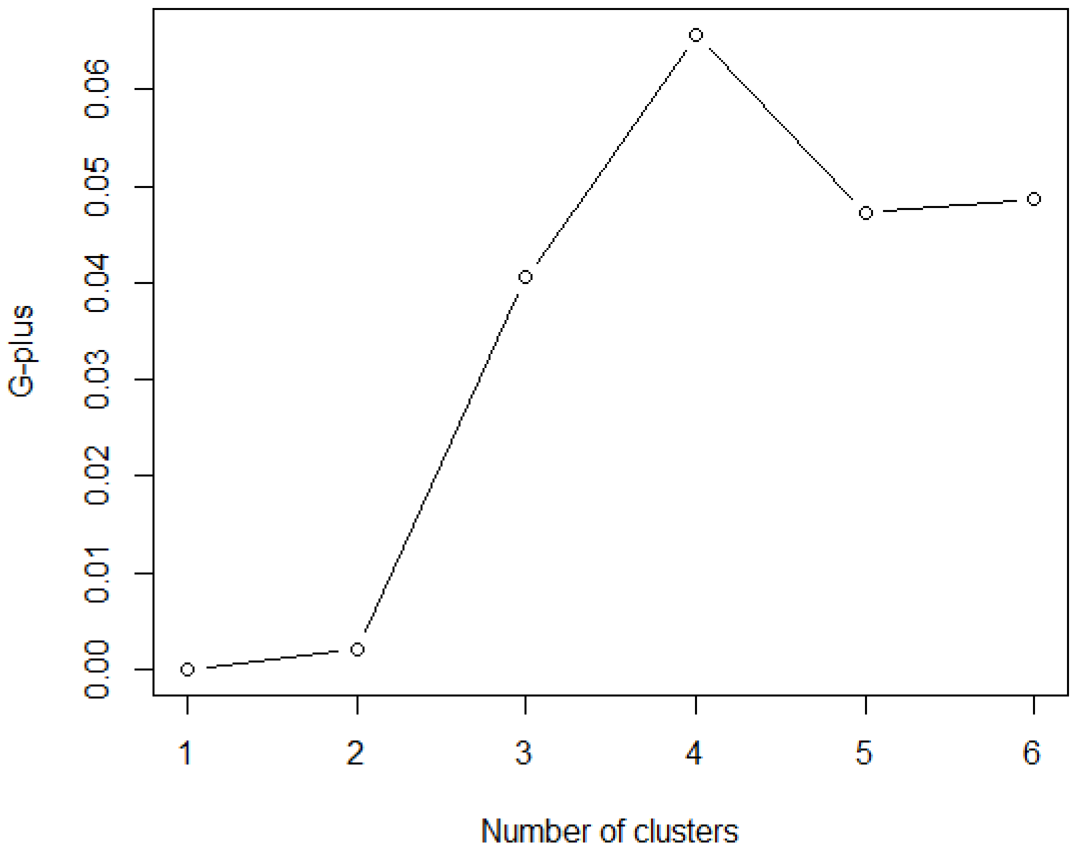

Supplementary Figure 3. GPlus plot for k= 1..6 number of clusters

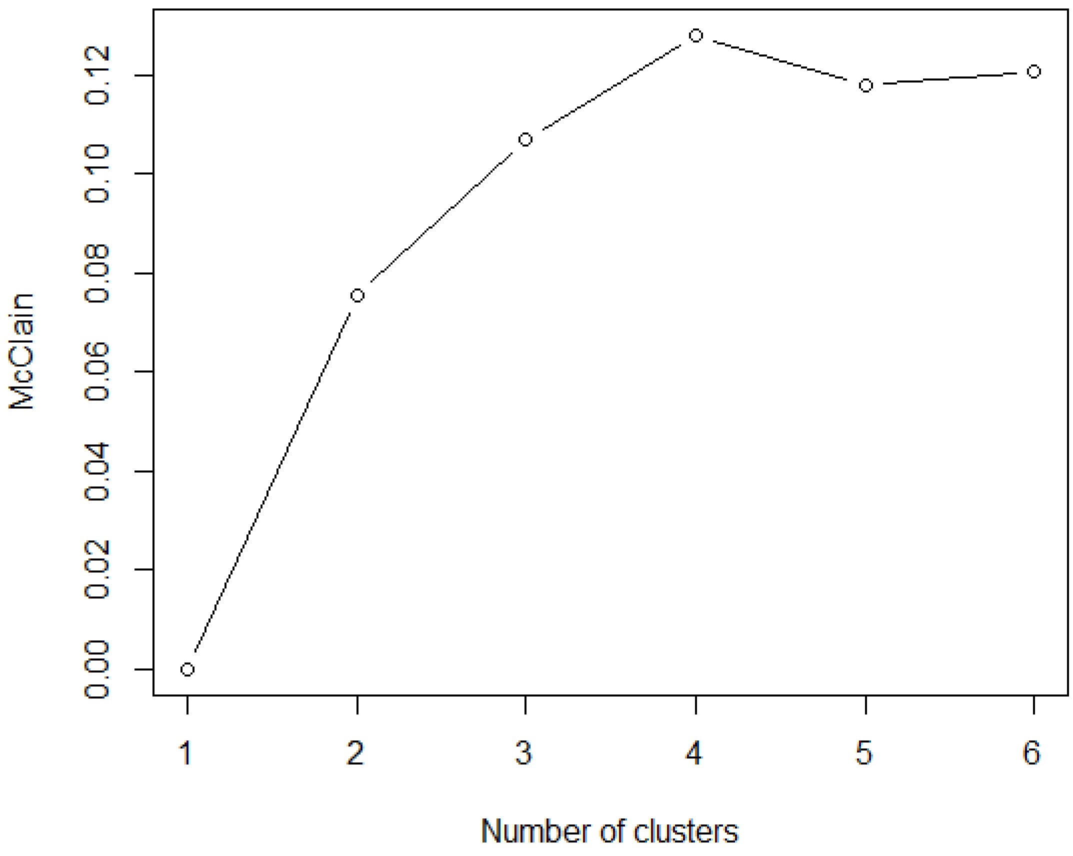

Supplementary Figure 4. McClain plot for k= 1..6 number of clusters

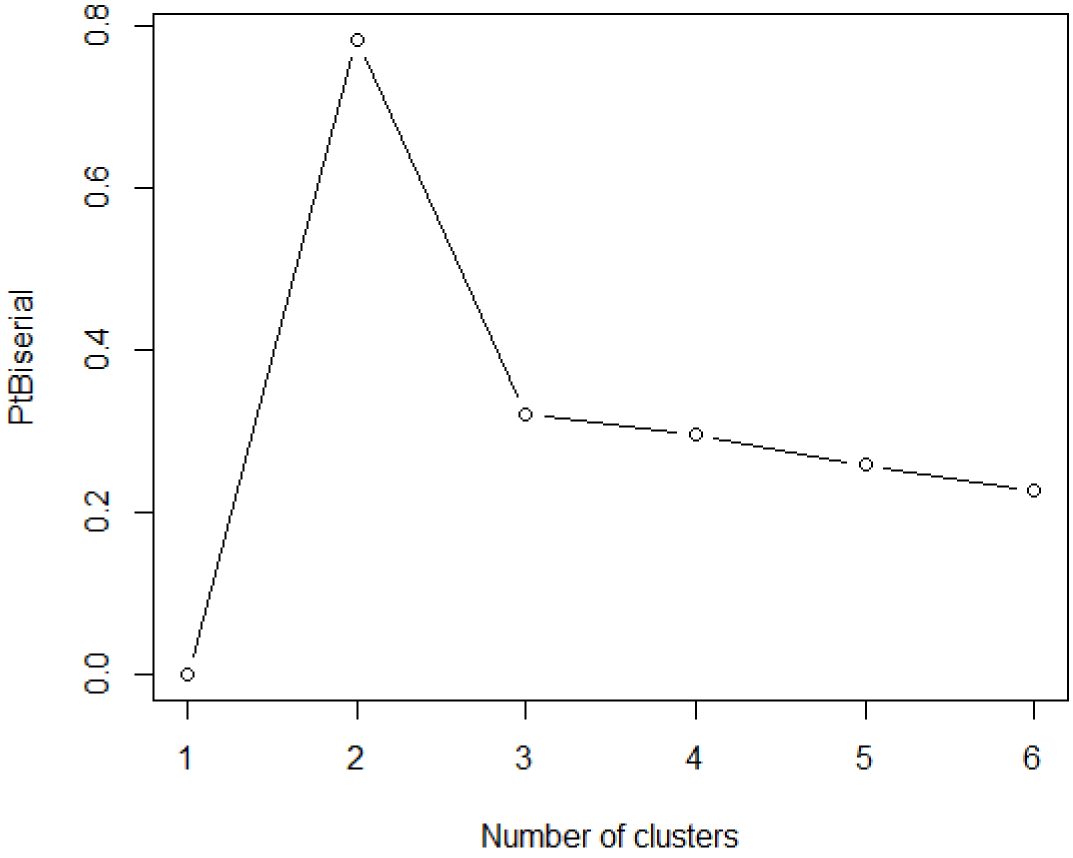

Supplementary Figure 5. PtBiseral plot for k= 1..6 number of clusters

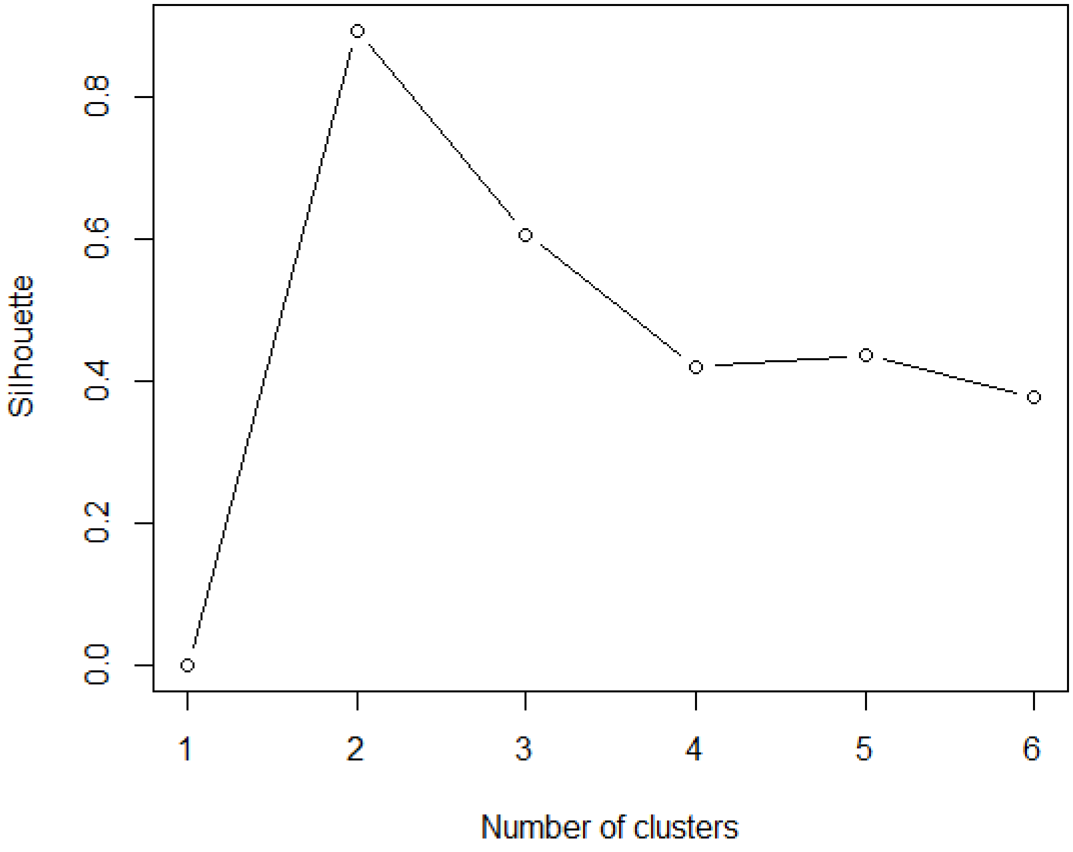

Supplementary Figure 6. Silhouette plot for k= 1..6 number of clusters

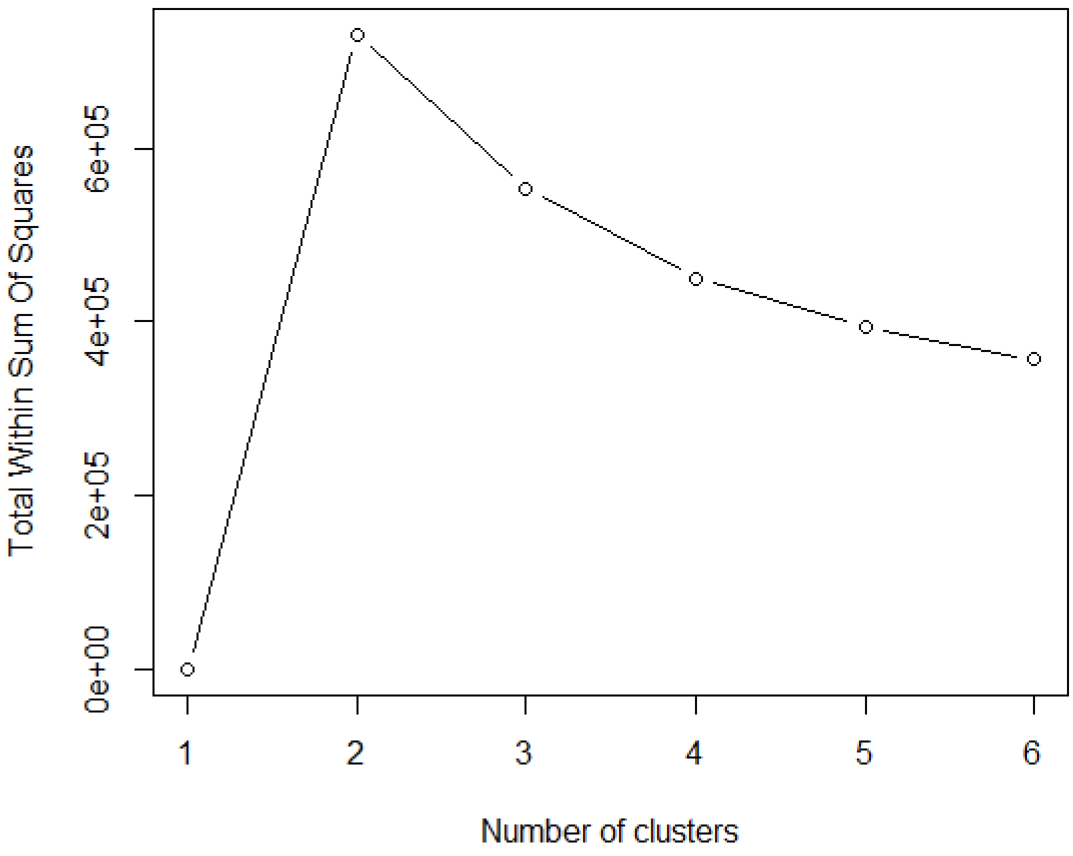

Supplementary Figure 7. Sum of squares plot for k= 1..6 number of clusters

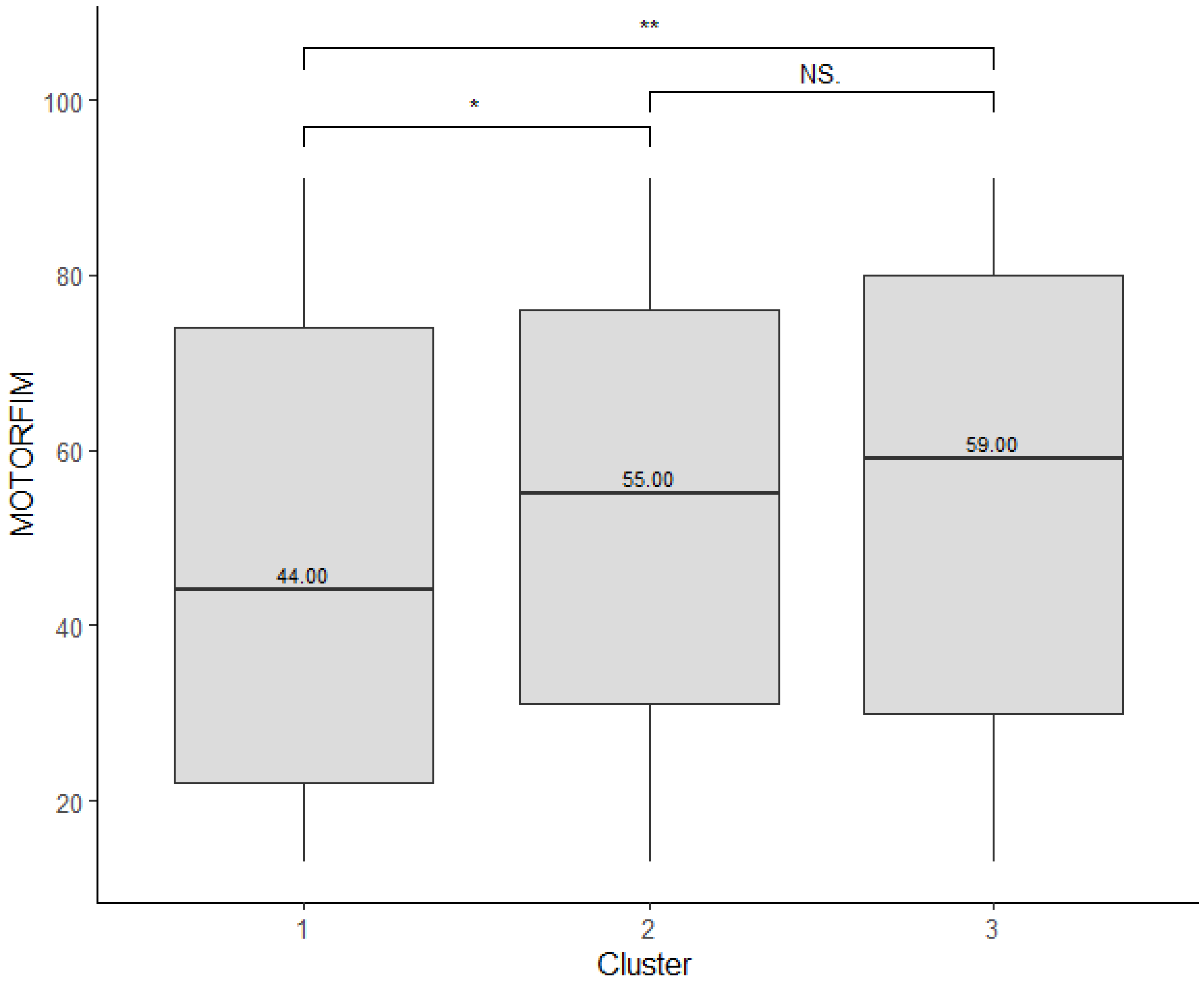

Supplementary Figure 8. Boxplots for motor FIM scores obtained by 85% of the initial N=1107 participants

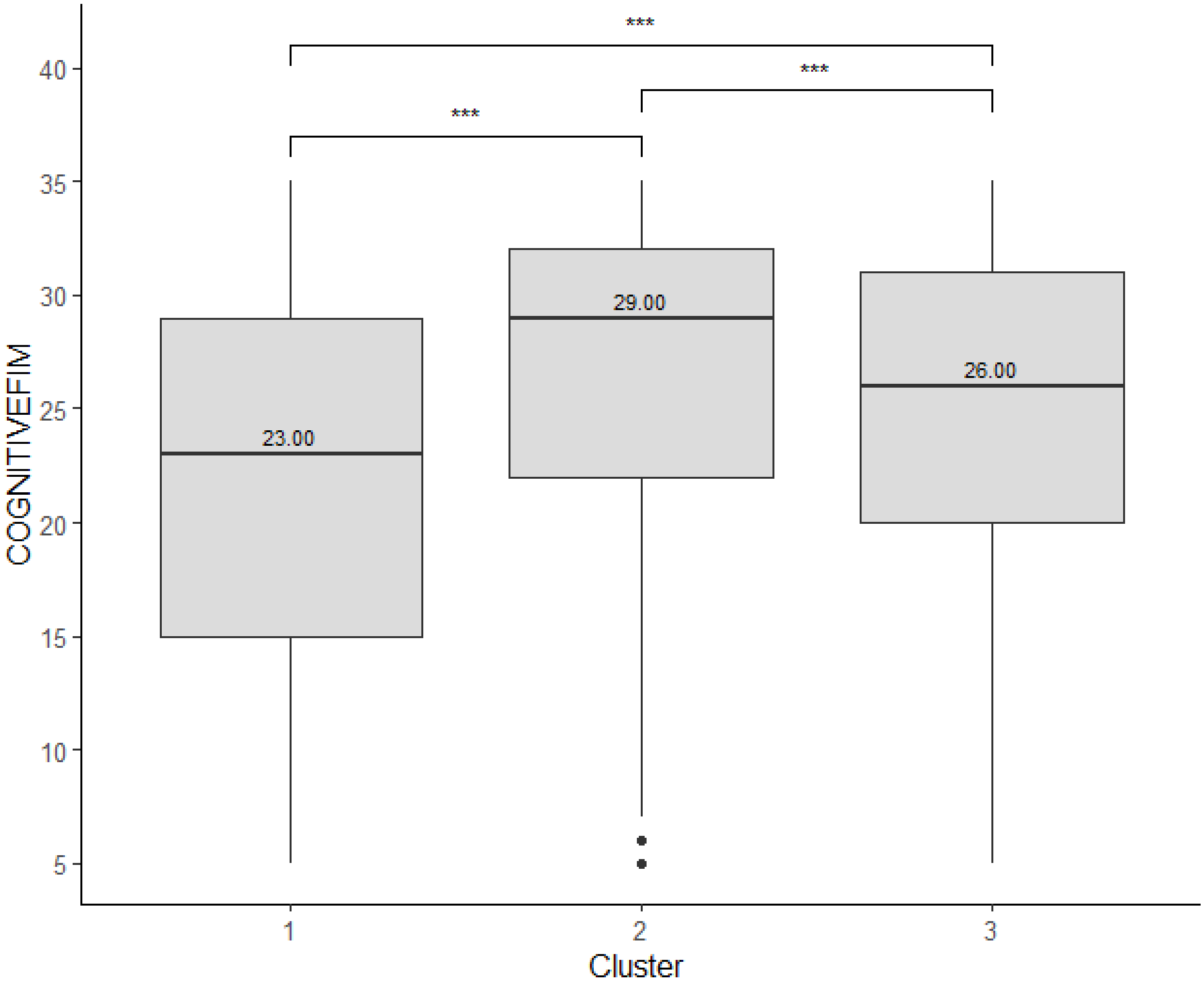

Supplementary Figure 9. Boxplots for cognitive FIM scores obtained by 85% of the initial N=1107 participants

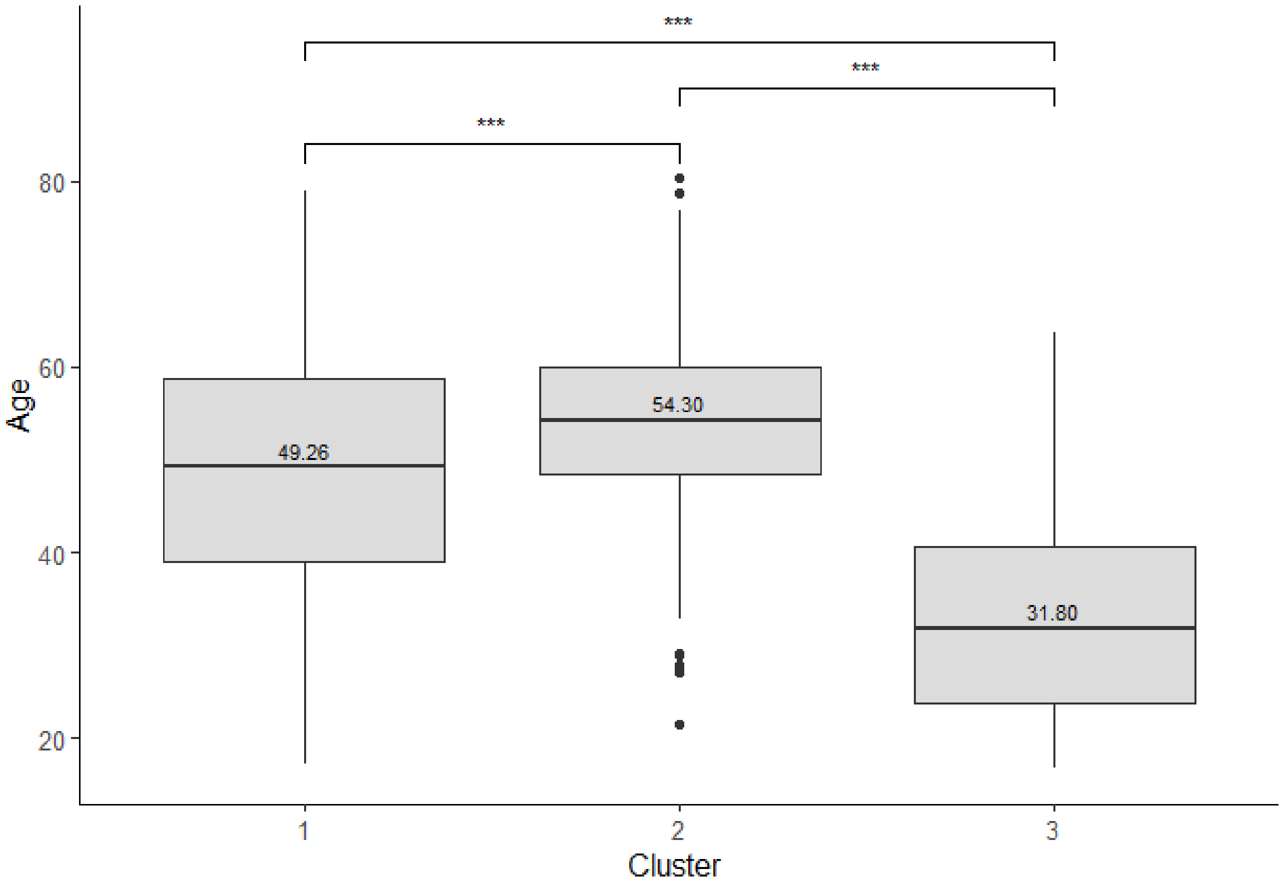

Supplementary Figure 10. Boxplots for age at the moment of neuropsychological assessment
