# Supplementary material for: Neuropsychological Assessments of Patients With Acquired Brain Injury: A Cluster Analysis Approach to Address Heterogeneity in Web-Based Cognitive Rehabilitation
Source: Front Neurol. 2021 Aug 9;12:701946. doi: 10.3389/fneur.2021.701946 (PMC8380987; doi:10.3389/fneur.2021.701946)

Supplementary material

Contents

Introduction 3
Injury 4
Only injury 4
Number of clusters 4
Optimal Clusters’ Silhouette (k=3) 4
Optimal Clusters’ Descriptive statistics 5
Optimal Clusters’ tSNE 6
Injury and studies 7
Number of clusters 7
Optimal Clusters’ Silhouette (k=5) 7
Optimal Clusters’ Descriptive statistics 8
Optimal Clusters’ tSNE 9
Injury and age 10
Number of clusters 10
Optimal Clusters’ Silhouette (k=5) 10
Optimal Clusters’ Descriptive statistics 11
Optimal Clusters’ tSNE 12
Injury and time since injury 13
Number of clusters 13
Optimal Clusters’ Silhouette (k=5) 13
Optimal Clusters’ Descriptive statistics 14
Optimal Clusters’ tSNE 15
Injury, studies and age 16
Number of clusters 16
Optimal Clusters’ Silhouette (k=5) 16
Optimal Clusters’ tSNE 17
Injury, studies and time since injury 18
Number of clusters 18
Optimal Clusters’ Silhouette (k=4) 18
Optimal Clusters’ tSNE 19
Injury, time since injury and age 20
Optimal Clusters’ Silhouette (k=4) 20
Optimal Clusters’ tSNE 21
Injury, studies, time since injury and age 22
Optimal Clusters’ Silhouette (k=5) 22
Optimal Clusters’ tSNE 23
Studies 24
Only studies 24
Number of clusters 24
Optimal Clusters’ Silhouette (k=3) 24
Optimal Clusters’ Descriptive statistics 25

Optimal Clusters' tSNE 26
Studies and age 27
Number of clusters 27
Optimal Clusters' Silhouette (k=6) 27
Optimal Clusters' tSNE 28
Studies and time since injury 29
Number of clusters 29
Optimal Clusters' Silhouette (k=8) 29
Optimal Clusters' tSNE 30
Studies, time since injury and age 31
Number of clusters 31
Optimal Clusters' Silhouette (k=5) 31
Optimal Clusters' tSNE 32
Age 33
Only age 33
Number of clusters 33
Optimal Clusters' Silhouette (k=3) 33
Optimal Clusters' Descriptive statistics 34
Optimal Clusters' tSNE 35
Age and time since injury 36
Number of clusters 36
Optimal Clusters' Silhouette (k=8) 36
Optimal Clusters' tSNE 37
Time since injury 38
Only time since injury 38
Number of clusters 38
Optimal Clusters' Silhouette (k=4) 38
Optimal Clusters' Descriptive statistics 39
Optimal Clusters' tSNE 40

Introduction

As presented in section 3.2 of the manuscript, the present study considers 7 numerical variables (T3, T5, T14, T15, T18, T22 and age) and a categorical variable (injury). In this Supplementary material document, we use the numerical variables (as well as other categorical variables such as stratified age and stratified time since injury) as input to the PAM clustering method with Gower distance. Different combinations of the 4 categorical variables have been analyzed and explored and assessed using the Silhouette index.
We also present the 2-dimensional projection of the obtained clusters using the t-Distributed Stochastic Neighbor Embedding (t-SNE) dimensionality reduction technique.

NOTES:
Categorical variables:
injury: (TBI, STROKE, OTHERABI)
studies: (primary, secondary, tertiary)
age: (17-30, 31-55, ≥56)
time since injury: (0-45, 46-90, 91-180, 181-364)

Numerical variables:
T3 (Temporal orientation): Test Barcelona
T5 (TMT-A): Sustained attention
T14 (cubss WAIS-III): planification
T15 (dígitos inversos WAIS-III): working memory [is interpreted in this work as a subdomain within executive functioning]
T18 (RAVLT. Delayed recall): memory
T22 (WCST. Perseverative errors): flexibility

Injury

Only injury

Number of clusters

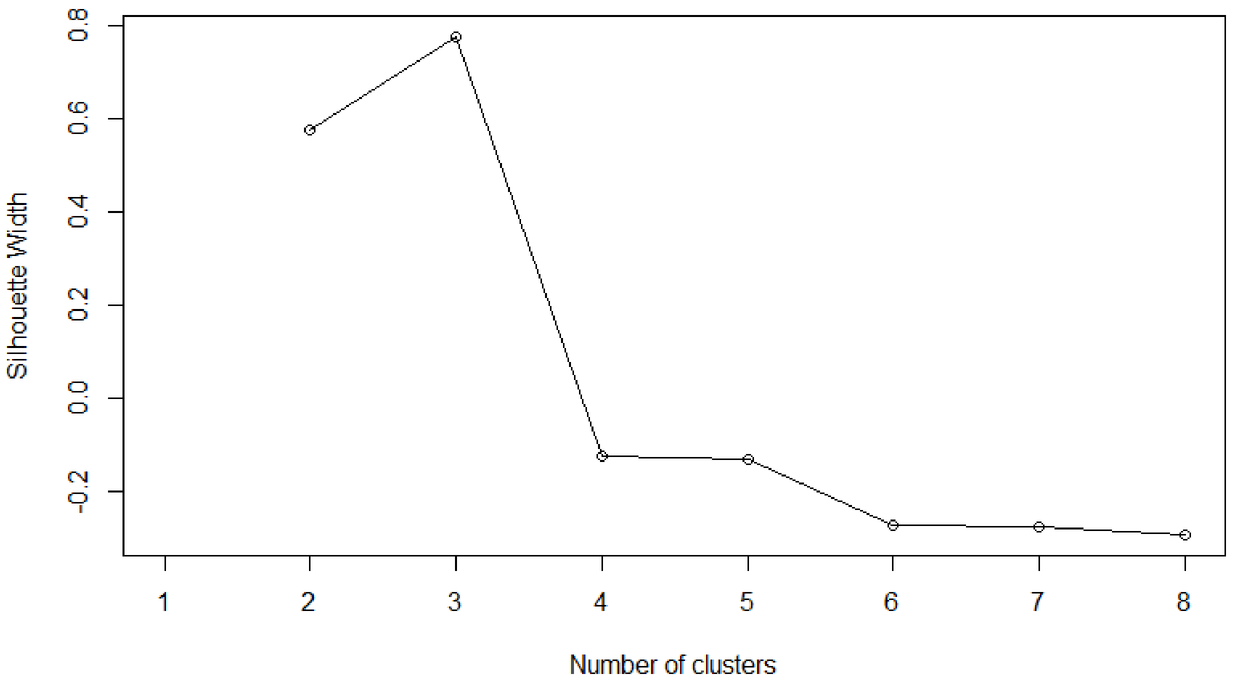

Optimal Clusters' Silhouette (k=3)

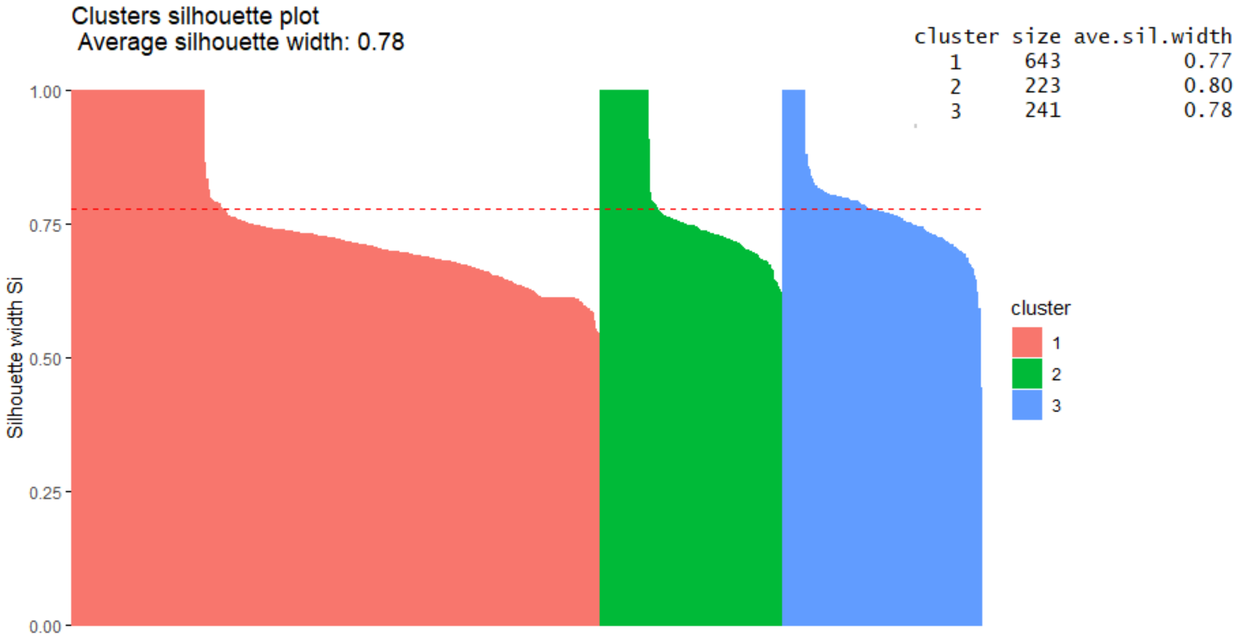

Optimal Clusters' Descriptive statistics

	1 (N=643)	2 (N=223)	3 (N=241)	Total (N=1107)	p value
T3					0.003
- Mean (SD)	20.054 (6.536)	19.655 (5.880)	21.425 (3.681)	20.318 (5.855)	
- Median (Q1, Q3)	23.000 (21.000, 23.000)	23.000 (18.000, 23.000)	23.000 (22.000, 23.000)	23.000 (22.000, 23.000)	
- Min - Max	0.000 - 23.000	0.000 - 23.000	0.000 - 23.000	0.000 - 23.000	
T5					0.002
- Mean (SD)	113.509 (96.156)	85.093 (56.011)	78.880 (58.272)	100.903 (84.446)	
- Median (Q1, Q3)	70.500 (48.000, 140.500)	71.000 (49.500, 108.000)	63.000 (40.000, 90.000)	68.000 (46.000, 119.000)	
- Min - Max	11.000 - 480.000	14.000 - 380.000	19.000 - 327.000	11.000 - 480.000	
T14					0.005
- Mean (SD)	25.387 (12.818)	20.714 (10.006)	24.159 (11.067)	24.217 (12.033)	
- Median (Q1, Q3)	24.000 (16.000, 33.000)	20.000 (12.000, 28.000)	24.000 (16.000, 30.000)	24.000 (14.750, 31.000)	
- Min - Max	1.000 - 68.000	1.000 - 46.000	1.000 - 58.000	1.000 - 68.000	
T15					0.015
- Mean (SD)	3.789 (1.020)	3.551 (0.955)	3.849 (0.986)	3.758 (1.003)	
- Median (Q1, Q3)	4.000 (3.000, 4.000)	4.000 (3.000, 4.000)	4.000 (3.000, 4.000)	4.000 (3.000, 4.000)	
- Min - Max	1.000 - 7.000	2.000 - 6.000	2.000 - 7.000	1.000 - 7.000	
T18					< 0.001
- Mean (SD)	3.618 (3.508)	4.361 (3.766)	6.332 (3.910)	4.435 (3.826)	
- Median (Q1, Q3)	3.000 (0.000, 6.000)	4.000 (1.000, 7.000)	6.000 (3.000, 9.000)	4.000 (1.000, 7.000)	
- Min - Max	0.000 - 15.000	0.000 - 14.000	0.000 - 15.000	0.000 - 15.000	
T22					< 0.001
- Mean (SD)	52.000 (38.979)	20.612 (14.570)	21.542 (15.710)	41.490 (35.953)	
- Median (Q1, Q3)	33.000 (18.000, 100.000)	16.000 (11.000, 30.000)	17.500 (9.000, 32.000)	27.000 (12.500, 70.500)	
- Min - Max	2.000 - 100.000	3.000 - 71.000	1.000 - 75.000	1.000 - 100.000	
type					< 0.001
- OTHERABI	0 (0.0%)	223 (100.0%)	0 (0.0%)	223 (20.1%)	
- STROKE	0 (0.0%)	0 (0.0%)	241 (100.0%)	241 (21.8%)	
- TBI	643 (100.0%)	0 (0.0%)	0 (0.0%)	643 (58.1%)	
studies					0.053
- primary	320 (49.8%)	97 (43.5%)	101 (41.9%)	518 (46.8%)	
- secondary	211 (32.8%)	75 (33.6%)	79 (32.8%)	365 (33.0%)	
- tertiary	112 (17.4%)	51 (22.9%)	61 (25.3%)	224 (20.2%)	
Age					< 0.001
- >55	102 (15.9%)	67 (30.0%)	91 (37.8%)	260 (23.5%)	
- 17-30	240 (37.3%)	33 (14.8%)	8 (3.3%)	281 (25.4%)	
- 31-55	301 (46.8%)	123 (55.2%)	142 (58.9%)	566 (51.1%)	
Agecont					< 0.001
- Mean (SD)	38.966 (14.951)	47.698 (14.085)	51.941 (9.505)	43.549 (14.840)	
- Median (Q1, Q3)	37.260 (26.023, 50.163)	48.803 (37.423, 57.871)	53.362 (46.485, 58.827)	44.466 (30.875, 55.107)	
- Min - Max	16.781 - 78.962	17.271 - 78.636	21.447 - 80.384	16.781 - 80.384	
TSOcont					< 0.001
- Mean (SD)	93.126 (65.123)	114.103 (80.095)	75.780 (58.779)	93.575 (68.215)	
- Median (Q1, Q3)	74.000 (48.000, 118.000)	91.000 (55.000, 156.500)	56.000 (37.000, 93.000)	73.000 (46.000, 117.500)	
- Min - Max	8.000 - 364.000	11.000 - 348.000	6.000 - 354.000	6.000 - 364.000	
TSO					< 0.001
- 0-45	140 (21.8%)	39 (17.5%)	91 (37.8%)	270 (24.4%)	
- 181-364	64 (10.0%)	45 (20.2%)	15 (6.2%)	124 (11.2%)	
- 46-90	258 (40.1%)	70 (31.4%)	82 (34.0%)	410 (37.0%)	
- 91-180	181 (28.1%)	69 (30.9%)	53 (22.0%)	303 (27.4%)	

Optimal Clusters' tSNE

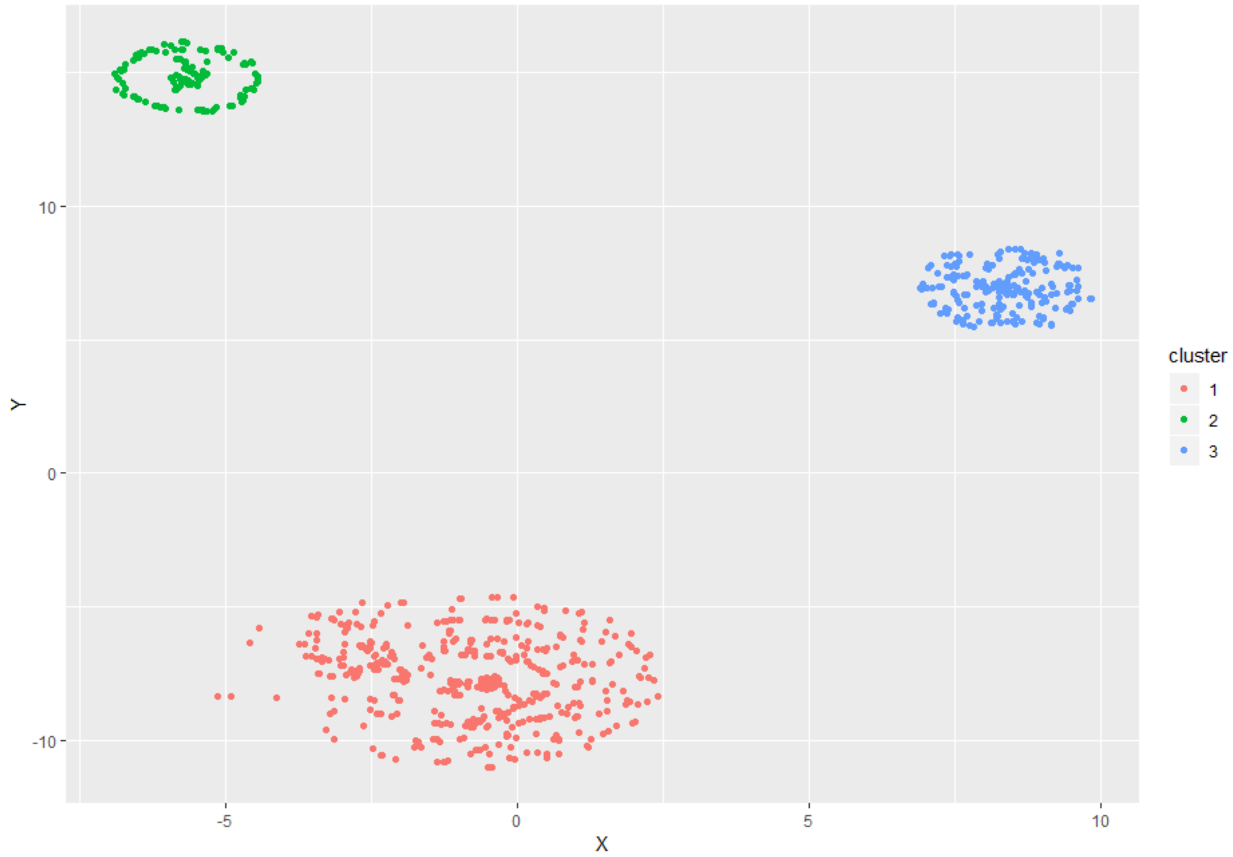

Injury and studies

Number of clusters

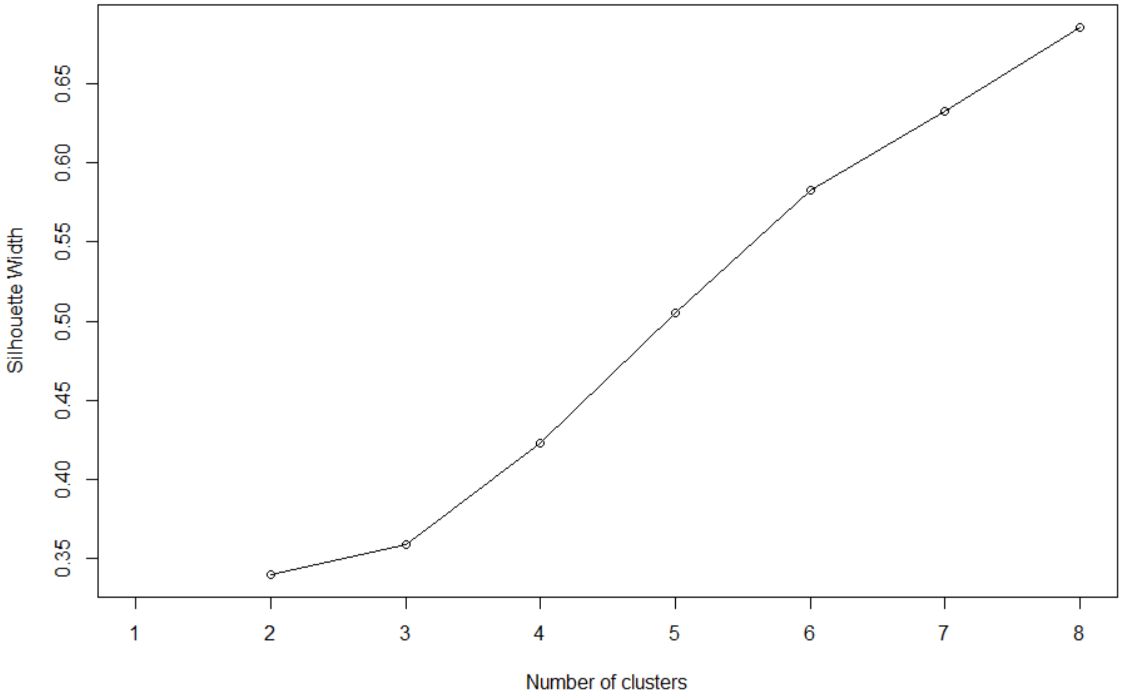

Optimal Clusters' Silhouette (k=5)

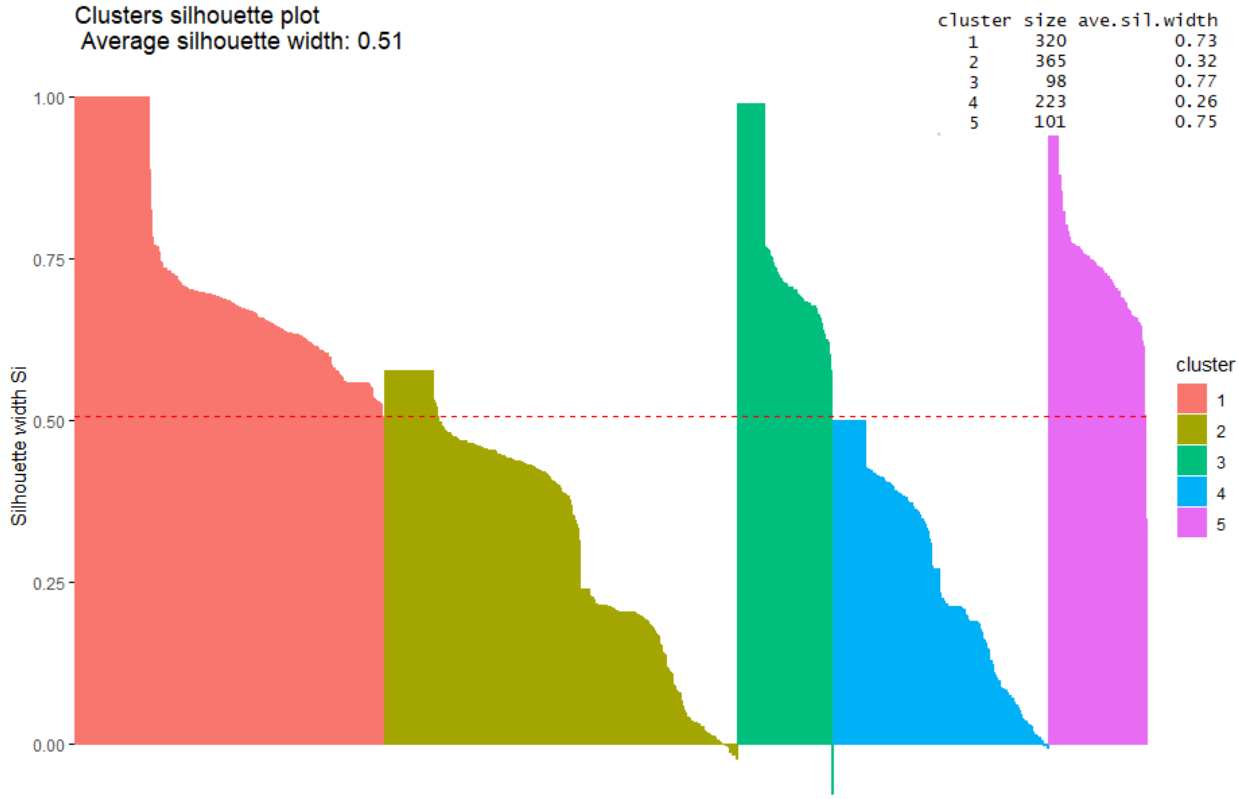

Optimal Clusters' Descriptive statistics

	1 (N=320)	2 (N=365)	3 (N=98)	4 (N=223)	5 (N=101)	Total (N=1107)	p value
T3							0.009
- Mean (SD)	19.646 (6.834)	20.608 (5.339)	19.732 (6.000)	20.288 (5.952)	21.722 (3.654)	20.318 (5.855)	
- Median (Q1, Q3)	23.000 (21.000, 23.000)	23.000 (21.750, 23.000)	23.000 (20.000, 23.000)	23.000 (22.000, 23.000)	23.000 (22.000, 23.000)	23.000 (22.000, 23.000)	
- Min - Max	0.000 - 23.000	0.000 - 23.000	0.000 - 23.000	0.000 - 23.000	0.000 - 23.000	0.000 - 23.000	
T5							0.013
- Mean (SD)	120.388 (99.099)	96.820 (79.674)	90.646 (68.518)	90.525 (79.438)	84.014 (59.659)	100.903 (84.446)	
- Median (Q1, Q3)	70.000 (51.000, 159.000)	70.000 (45.000, 115.000)	73.500 (48.500, 110.000)	61.000 (39.000, 100.000)	71.000 (44.000, 93.000)	68.000 (46.000, 119.000)	
- Min - Max	15.000 - 480.000	11.000 - 476.000	14.000 - 380.000	18.000 - 300.000	20.000 - 327.000	11.000 - 480.000	
T14							< 0.001
- Mean (SD)	22.422 (11.501)	25.074 (12.421)	19.696 (9.505)	27.717 (12.583)	22.908 (10.834)	24.217 (12.033)	
- Median (Q1, Q3)	20.000 (12.000, 28.000)	24.000 (16.000, 32.500)	16.000 (12.000, 28.000)	28.000 (20.000, 34.500)	24.000 (16.000, 28.000)	24.000 (14.750, 31.000)	
- Min - Max	4.000 - 59.000	1.000 - 68.000	2.000 - 40.000	1.000 - 56.000	1.000 - 58.000	1.000 - 68.000	
T15							< 0.001
- Mean (SD)	3.602 (0.953)	3.800 (0.940)	3.284 (0.884)	4.125 (1.120)	3.693 (0.951)	3.758 (1.003)	
- Median (Q1, Q3)	4.000 (3.000, 4.000)	4.000 (3.000, 4.000)	3.000 (3.000, 4.000)	4.000 (3.000, 5.000)	4.000 (3.000, 4.000)	4.000 (3.000, 4.000)	
- Min - Max	2.000 - 6.000	1.000 - 6.000	2.000 - 5.000	2.000 - 7.000	2.000 - 6.000	1.000 - 7.000	
T18							< 0.001
- Mean (SD)	3.407 (3.362)	4.428 (3.847)	3.866 (3.709)	5.506 (4.086)	5.711 (3.714)	4.435 (3.826)	
- Median (Q1, Q3)	2.000 (0.000, 5.500)	4.000 (1.000, 7.000)	3.000 (0.000, 7.000)	5.000 (2.000, 9.000)	6.000 (3.000, 8.000)	4.000 (1.000, 7.000)	
- Min - Max	0.000 - 13.000	0.000 - 14.000	0.000 - 11.000	0.000 - 15.000	0.000 - 15.000	0.000 - 15.000	
T22							< 0.001
- Mean (SD)	58.491 (38.540)	38.359 (35.175)	20.857 (13.660)	31.747 (32.163)	27.000 (16.752)	41.490 (35.953)	
- Median (Q1, Q3)	46.500 (21.500, 100.000)	24.500 (12.000, 49.250)	15.500 (11.000, 33.000)	22.000 (9.000, 35.500)	26.500 (13.000, 33.750)	27.000 (12.500, 70.500)	
- Min - Max	2.000 - 100.000	3.000 - 100.000	5.000 - 44.000	1.000 - 100.000	7.000 - 75.000	1.000 - 100.000	
type							< 0.001
- OTHERABI	0 (0.0%)	75 (20.5%)	98 (100.0%)	50 (22.4%)	0 (0.0%)	223 (20.1%)	
- STROKE	0 (0.0%)	79 (21.6%)	0 (0.0%)	61 (27.4%)	101 (100.0%)	241 (21.8%)	
- TBI	320 (100.0%)	211 (57.8%)	0 (0.0%)	112 (50.2%)	0 (0.0%)	643 (58.1%)	
studies							< 0.001
- primary	320 (100.0%)	0 (0.0%)	97 (99.0%)	0 (0.0%)	101 (100.0%)	518 (46.8%)	
- secondary	0 (0.0%)	365 (100.0%)	0 (0.0%)	0 (0.0%)	0 (0.0%)	365 (33.0%)	
- tertiary	0 (0.0%)	0 (0.0%)	1 (1.0%)	223 (100.0%)	0 (0.0%)	224 (20.2%)	
Age							< 0.001
- >55	58 (18.1%)	67 (18.4%)	35 (35.7%)	65 (29.1%)	35 (34.7%)	260 (23.5%)	
- 17-30	105 (32.8%)	116 (31.8%)	11 (11.2%)	46 (20.6%)	3 (3.0%)	281 (25.4%)	
- 31-55	157 (49.1%)	182 (49.9%)	52 (53.1%)	112 (50.2%)	63 (62.4%)	566 (51.1%)	
Agecont							< 0.001
- Mean (SD)	40.640 (14.726)	40.987 (15.624)	49.667 (13.486)	45.624 (14.073)	51.511 (8.820)	43.549 (14.840)	
- Median (Q1, Q3)	40.068 (28.505, 51.827)	41.274 (27.159, 52.504)	51.533 (40.929, 59.246)	46.332 (33.032, 57.942)	52.786 (46.786, 57.685)	44.466 (30.875, 55.107)	
- Min - Max	16.781 - 76.241	16.811 - 80.384	18.745 - 73.937	20.082 - 76.904	22.605 - 73.268	16.781 - 80.384	
TSOcont							< 0.001
- Mean (SD)	94.369 (63.531)	96.252 (70.263)	115.796 (77.280)	86.193 (68.533)	76.129 (58.813)	93.575 (68.215)	
- Median (Q1, Q3)	76.000 (50.000, 118.000)	74.000 (48.000, 121.000)	101.500 (55.250, 160.000)	66.000 (38.000, 105.000)	54.000 (39.000, 91.000)	73.000 (46.000, 117.500)	
- Min - Max	9.000 - 364.000	6.000 - 364.000	11.000 - 335.000	12.000 - 351.000	10.000 - 317.000	6.000 - 364.000	
TSO							< 0.001
- 0-45	64 (20.0%)	82 (22.5%)	16 (16.3%)	70 (31.4%)	38 (37.6%)	270 (24.4%)	
- 181-364	29 (9.1%)	49 (13.4%)	20 (20.4%)	20 (9.0%)	6 (5.9%)	124 (11.2%)	
- 46-90	129 (40.3%)	141 (38.6%)	29 (29.6%)	76 (34.1%)	35 (34.7%)	410 (37.0%)	
- 91-180	98 (30.6%)	93 (25.5%)	33 (33.7%)	57 (25.6%)	22 (21.8%)	303 (27.4%)	

Optimal Clusters' tSNE

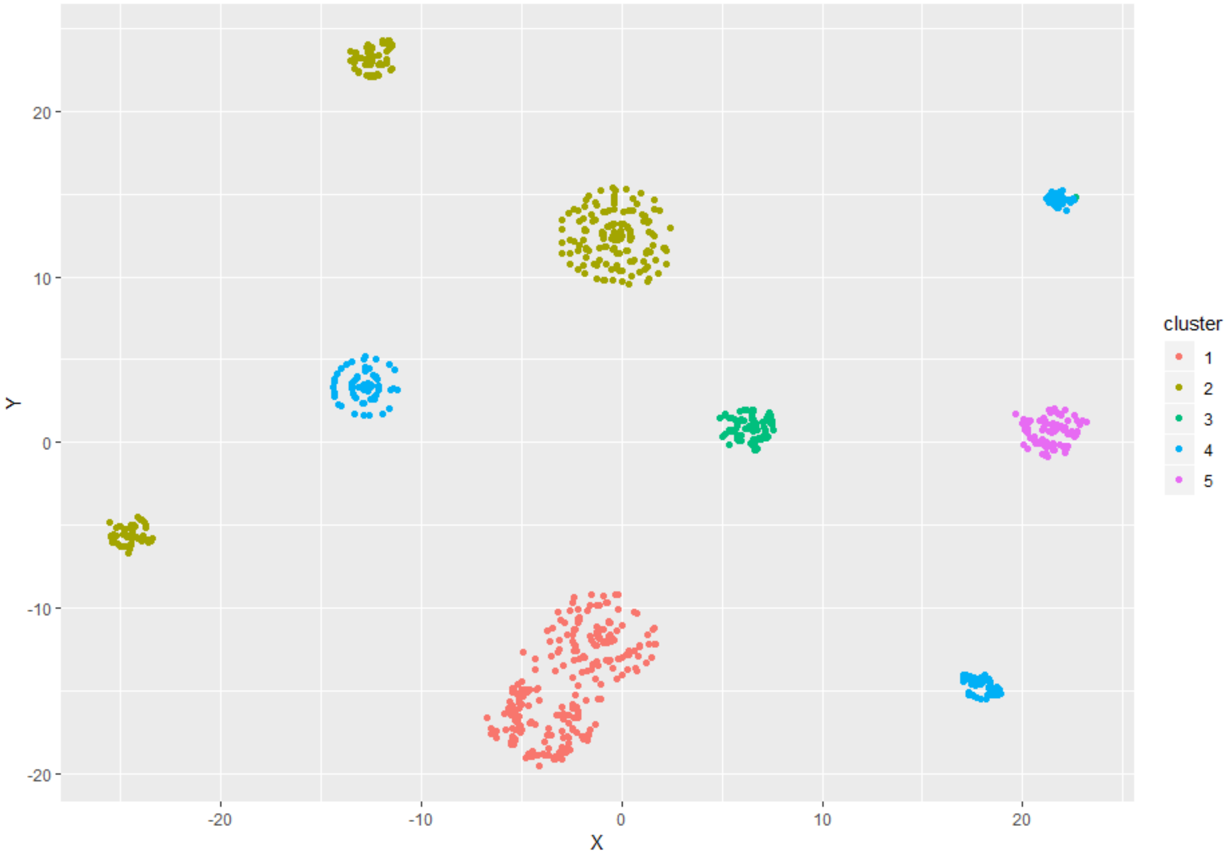

Injury and age

Number of clusters

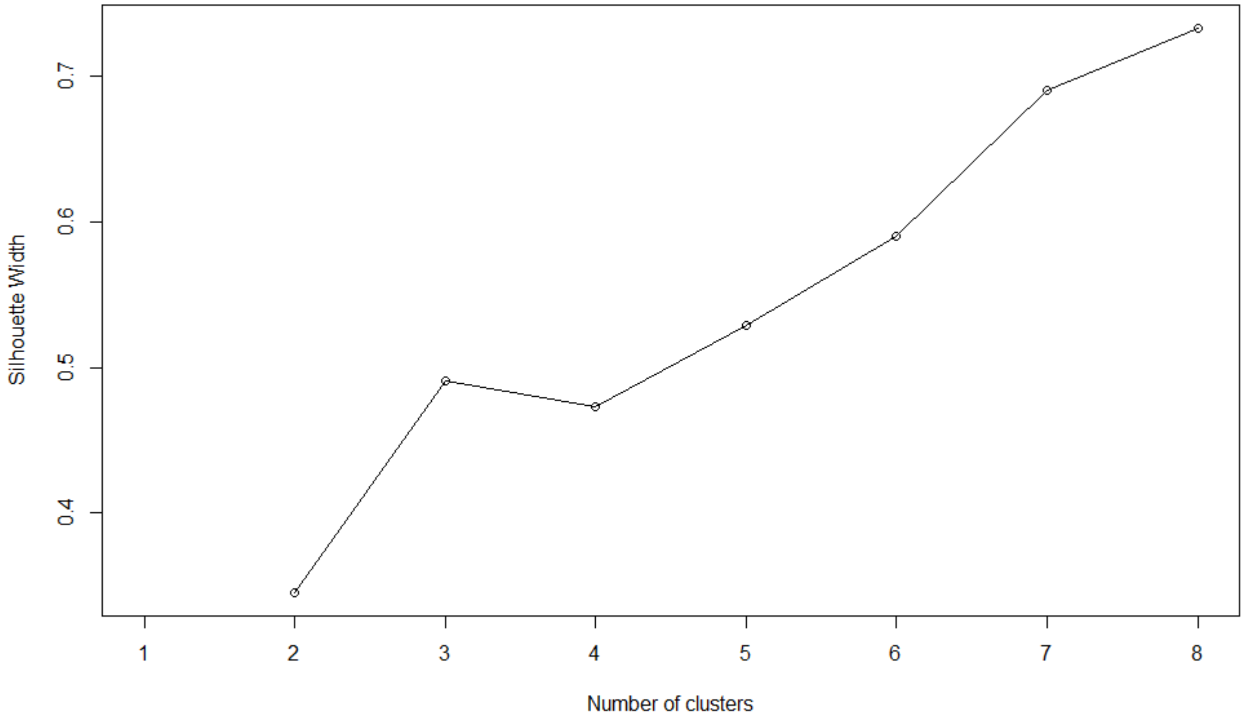

Optimal Clusters' Silhouette (k=5)

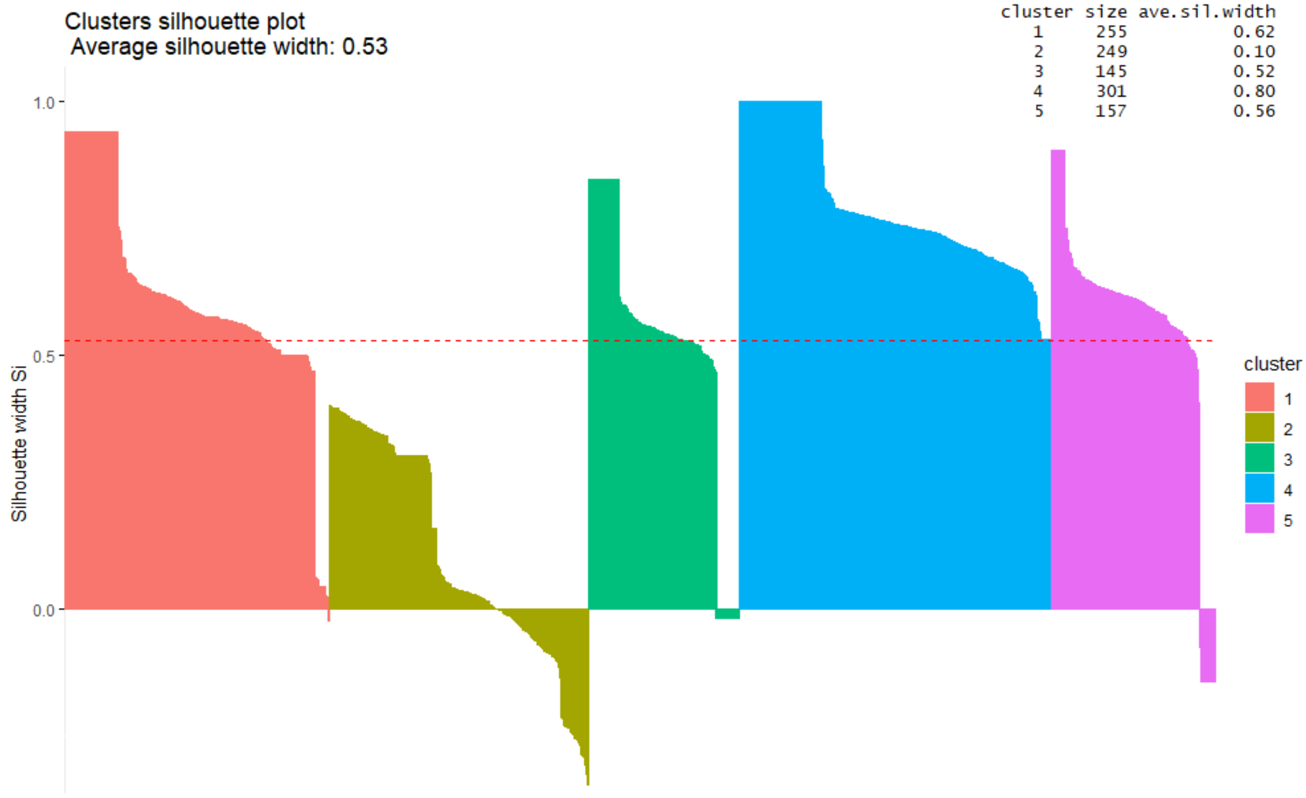

Optimal Clusters' Descriptive statistics

	1 (N=255)	2 (N=249)	3 (N=145)	4 (N=301)	5 (N=157)	Total (N=1107)	p value
T3							0.016
- Mean (SD)	18.726 (7.893)	20.872 (4.600)	19.532 (6.127)	20.752 (5.537)	21.623 (3.451)	20.318 (5.855)	
- Median (Q1, Q3)	23.000 (20.000, 23.000)	23.000 (22.000, 23.000)	23.000 (17.000, 23.000)	23.000 (22.000, 23.000)	23.000 (22.000, 23.000)	23.000 (22.000, 23.000)	
- Min - Max	0.000 - 23.000	0.000 - 23.000	0.000 - 23.000	0.000 - 23.000	0.000 - 23.000	0.000 - 23.000	
T5							< 0.001
- Mean (SD)	126.333 (102.422)	103.036 (73.519)	82.358 (55.334)	99.184 (90.613)	69.333 (52.225)	100.903 (84.446)	
- Median (Q1, Q3)	80.000 (49.000, 180.000)	81.000 (53.000, 133.500)	75.000 (48.500, 107.000)	63.000 (45.000, 96.250)	56.000 (35.500, 80.000)	68.000 (46.000, 119.000)	
- Min - Max	15.000 - 340.000	20.000 - 480.000	14.000 - 380.000	11.000 - 476.000	19.000 - 327.000	11.000 - 480.000	
T14							< 0.001
- Mean (SD)	26.707 (13.297)	20.642 (10.058)	22.214 (9.953)	25.587 (12.965)	25.871 (11.434)	24.217 (12.033)	
- Median (Q1, Q3)	27.000 (16.000, 36.000)	20.000 (12.000, 28.000)	24.000 (14.500, 29.000)	24.000 (16.000, 33.500)	25.000 (18.000, 32.000)	24.000 (14.750, 31.000)	
- Min - Max	2.000 - 68.000	1.000 - 51.000	2.000 - 46.000	4.000 - 59.000	1.000 - 58.000	1.000 - 68.000	
T15							0.091
- Mean (SD)	3.801 (1.074)	3.645 (1.038)	3.618 (0.959)	3.824 (0.984)	3.877 (0.889)	3.758 (1.003)	
- Median (Q1, Q3)	4.000 (3.000, 5.000)	4.000 (3.000, 4.000)	4.000 (3.000, 4.000)	4.000 (3.000, 4.000)	4.000 (3.000, 4.000)	4.000 (3.000, 4.000)	
- Min - Max	1.000 - 7.000	2.000 - 7.000	2.000 - 6.000	2.000 - 6.000	2.000 - 6.000	1.000 - 7.000	
T18							< 0.001
- Mean (SD)	3.769 (3.729)	4.340 (3.712)	4.625 (3.643)	3.719 (3.409)	6.725 (4.144)	4.435 (3.826)	
- Median (Q1, Q3)	3.000 (0.000, 6.000)	4.000 (1.000, 7.000)	4.000 (1.000, 7.000)	3.000 (1.000, 6.000)	7.000 (3.000, 10.000)	4.000 (1.000, 7.000)	
- Min - Max	0.000 - 14.000	0.000 - 14.000	0.000 - 12.000	0.000 - 15.000	0.000 - 15.000	0.000 - 15.000	
T22							< 0.001
- Mean (SD)	53.383 (39.938)	35.750 (30.894)	19.250 (12.425)	50.719 (37.950)	18.462 (12.535)	41.490 (35.953)	
- Median (Q1, Q3)	35.000 (17.500, 100.000)	27.000 (11.500, 41.500)	16.500 (10.500, 27.750)	31.000 (19.500, 100.000)	14.500 (9.000, 29.250)	27.000 (12.500, 70.500)	
- Min - Max	2.000 - 100.000	3.000 - 100.000	3.000 - 44.000	2.000 - 100.000	1.000 - 48.000	1.000 - 100.000	
type							< 0.001
- OTHERABI	12 (4.7%)	66 (26.5%)	145 (100.0%)	0 (0.0%)	0 (0.0%)	223 (20.1%)	
- STROKE	3 (1.2%)	81 (32.5%)	0 (0.0%)	0 (0.0%)	157 (100.0%)	241 (21.8%)	
- TBI	240 (94.1%)	102 (41.0%)	0 (0.0%)	301 (100.0%)	0 (0.0%)	643 (58.1%)	
studies							0.021
- primary	110 (43.1%)	119 (47.8%)	63 (43.4%)	157 (52.2%)	69 (43.9%)	518 (46.8%)	
- secondary	104 (40.8%)	69 (27.7%)	46 (31.7%)	90 (29.9%)	56 (35.7%)	365 (33.0%)	
- tertiary	41 (16.1%)	61 (24.5%)	36 (24.8%)	54 (17.9%)	32 (20.4%)	224 (20.2%)	
Age							< 0.001
- >55	0 (0.0%)	223 (89.6%)	22 (15.2%)	0 (0.0%)	15 (9.6%)	260 (23.5%)	
- 17-30	255 (100.0%)	26 (10.4%)	0 (0.0%)	0 (0.0%)	0 (0.0%)	281 (25.4%)	
- 31-55	0 (0.0%)	0 (0.0%)	123 (84.8%)	301 (100.0%)	142 (90.4%)	566 (51.1%)	
Agecont							< 0.001
- Mean (SD)	23.700 (4.155)	58.771 (12.497)	47.639 (9.375)	42.932 (6.825)	49.054 (7.314)	43.549 (14.840)	
- Median (Q1, Q3)	23.441 (20.081, 27.492)	60.499 (57.893, 64.907)	47.827 (40.888, 53.942)	42.101 (36.770, 48.740)	49.416 (44.614, 53.696)	44.466 (30.875, 55.107)	
- Min - Max	16.781 - 30.995	17.271 - 78.962	31.055 - 72.036	31.126 - 55.819	31.811 - 80.384	16.781 - 80.384	
TSOcont							0.002
- Mean (SD)	90.808 (65.511)	92.888 (70.254)	115.614 (83.496)	91.950 (60.745)	81.924 (63.392)	93.575 (68.215)	
- Median (Q1, Q3)	71.000 (46.000, 115.000)	71.000 (48.000, 113.000)	92.000 (54.000, 165.000)	73.000 (50.000, 117.000)	64.000 (39.000, 107.000)	73.000 (46.000, 117.500)	
- Min - Max	9.000 - 364.000	6.000 - 364.000	11.000 - 348.000	8.000 - 349.000	10.000 - 354.000	6.000 - 364.000	
TSO							< 0.001
- 0-45	63 (24.7%)	59 (23.7%)	29 (20.0%)	61 (20.3%)	58 (36.9%)	270 (24.4%)	
- 181-364	26 (10.2%)	25 (10.0%)	32 (22.1%)	28 (9.3%)	13 (8.3%)	124 (11.2%)	
- 46-90	100 (39.2%)	97 (39.0%)	41 (28.3%)	125 (41.5%)	47 (29.9%)	410 (37.0%)	
- 91-180	66 (25.9%)	68 (27.3%)	43 (29.7%)	87 (28.9%)	39 (24.8%)	303 (27.4%)	

<u>*Optimal Clusters' tSNE*</u>

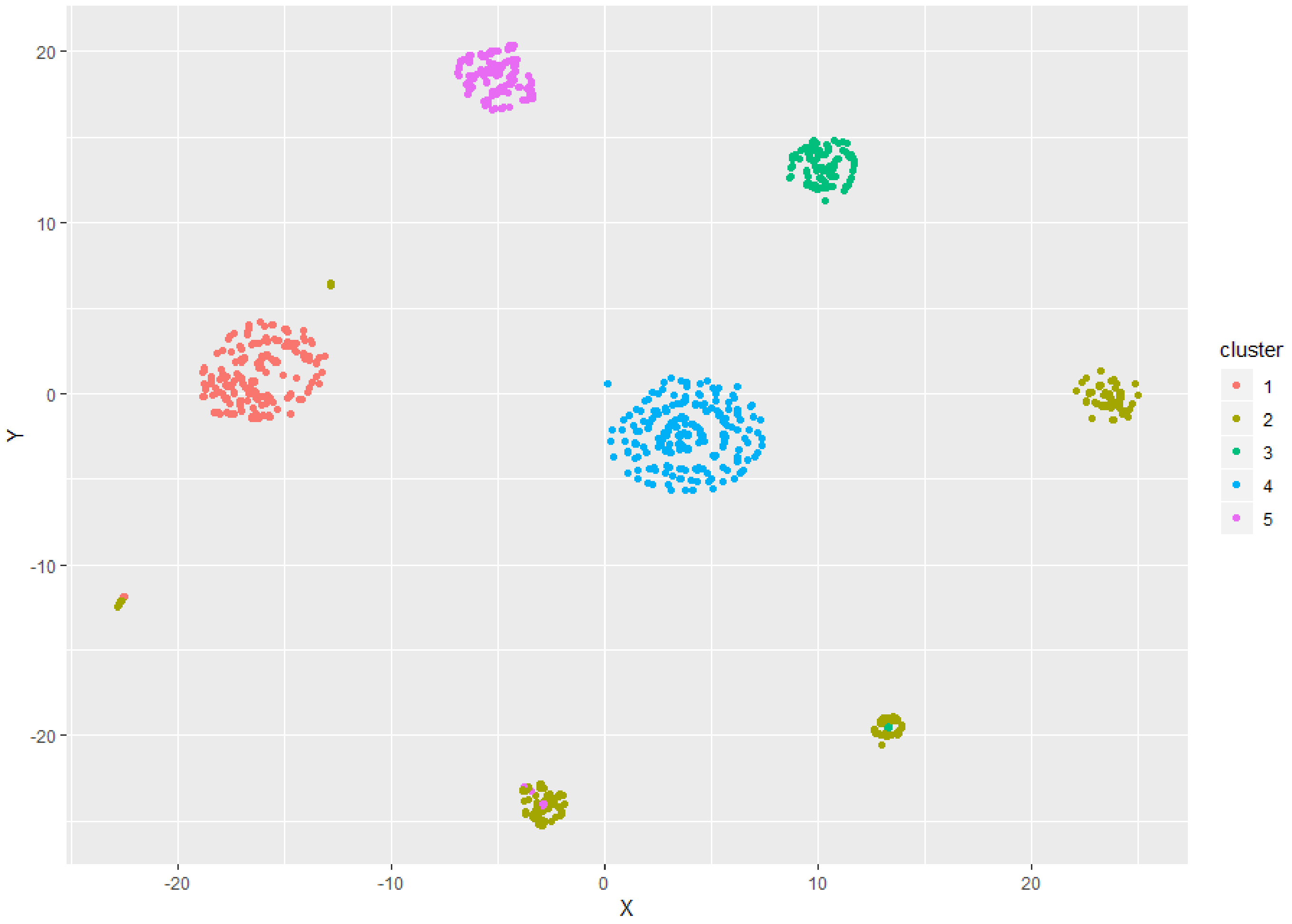

Injury and time since injury

Number of clusters

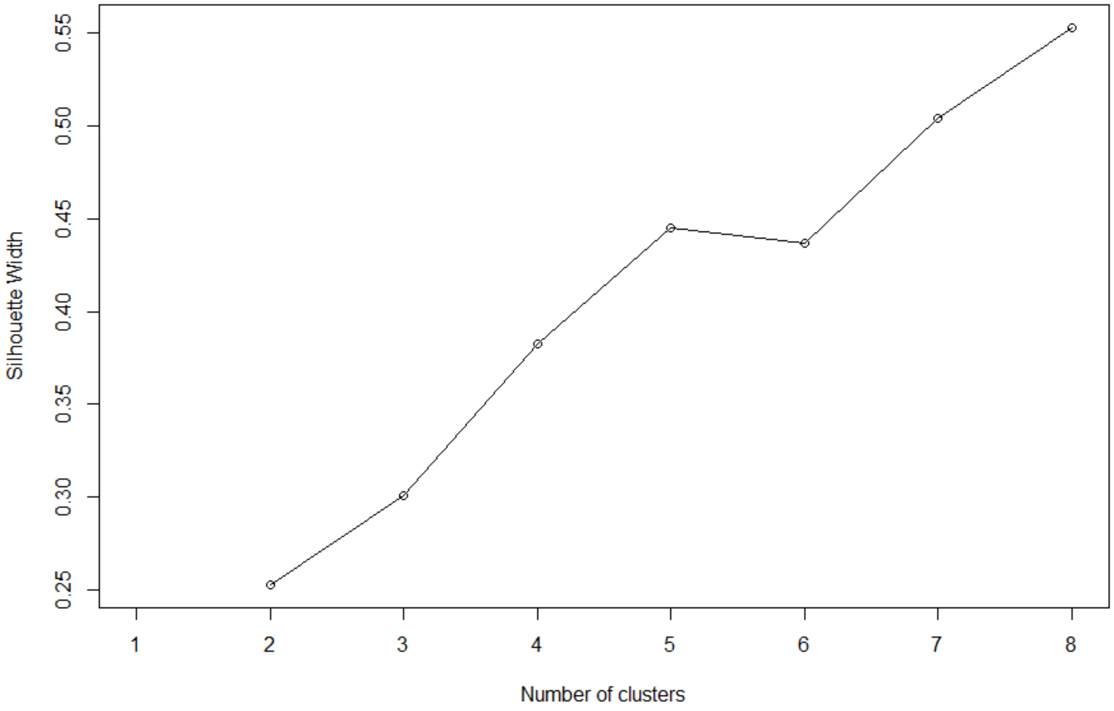

Optimal Clusters' Silhouette (k=5)

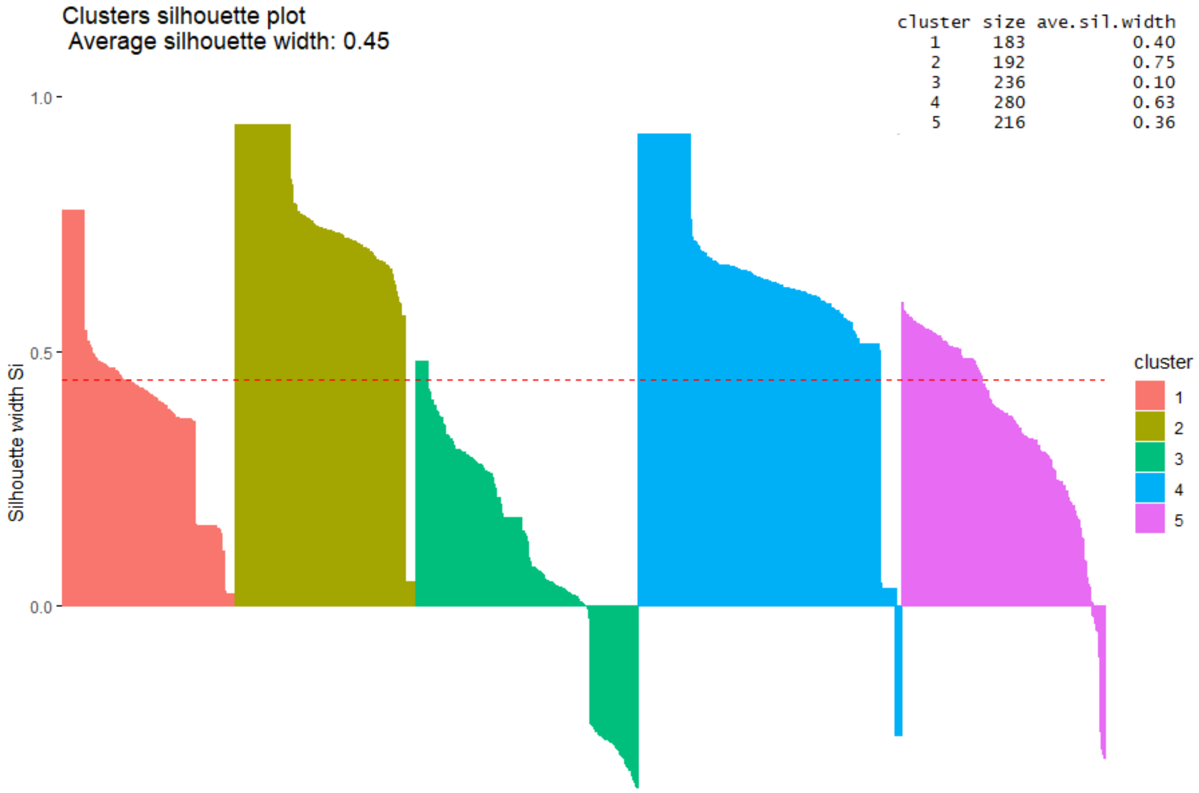

Optimal Clusters' Descriptive statistics

	1 (N=183)	2 (N=192)	3 (N=236)	4 (N=280)	5 (N=216)	Total (N=1107)	p value
T3							0.003
- Mean (SD)	19.461 (7.555)	20.959 (4.918)	19.782 (5.529)	19.502 (7.277)	21.792 (2.881)	20.318 (5.855)	
- Median (Q1, Q3)	23.000 (21.000, 23.000)	23.000 (22.000, 23.000)	23.000 (18.000, 23.000)	23.000 (21.000, 23.000)	23.000 (22.000, 23.000)	23.000 (22.000, 23.000)	
- Min - Max	0.000 - 23.000	0.000 - 23.000	0.000 - 23.000	0.000 - 23.000	7.000 - 23.000	0.000 - 23.000	
T5							< 0.001
- Mean (SD)	96.679 (89.346)	124.465 (98.427)	88.503 (60.114)	121.124 (100.584)	76.856 (57.017)	100.903 (84.446)	
- Median (Q1, Q3)	60.000 (45.000, 104.250)	90.000 (50.500, 167.000)	75.000 (52.000, 109.000)	77.000 (50.000, 153.750)	62.000 (38.000, 89.250)	68.000 (46.000, 119.000)	
- Min - Max	11.000 - 300.000	15.000 - 476.000	14.000 - 380.000	20.000 - 480.000	19.000 - 300.000	11.000 - 480.000	
T14							< 0.001
- Mean (SD)	27.161 (12.846)	24.187 (13.506)	19.782 (9.781)	25.686 (12.468)	25.050 (11.113)	24.217 (12.033)	
- Median (Q1, Q3)	26.000 (16.000, 36.000)	22.000 (12.000, 31.000)	18.000 (12.000, 27.750)	25.000 (16.000, 32.000)	25.000 (16.000, 31.000)	24.000 (14.750, 31.000)	
- Min - Max	4.000 - 68.000	2.000 - 59.000	1.000 - 47.000	1.000 - 63.000	1.000 - 58.000	1.000 - 68.000	
T15							< 0.001
- Mean (SD)	3.898 (1.076)	3.622 (1.054)	3.393 (0.902)	3.873 (0.972)	4.005 (0.952)	3.758 (1.003)	
- Median (Q1, Q3)	4.000 (3.000, 4.250)	4.000 (3.000, 4.000)	3.000 (3.000, 4.000)	4.000 (3.000, 5.000)	4.000 (3.000, 5.000)	4.000 (3.000, 4.000)	
- Min - Max	2.000 - 7.000	1.000 - 6.000	2.000 - 6.000	2.000 - 7.000	2.000 - 7.000	1.000 - 7.000	
T18							< 0.001
- Mean (SD)	4.400 (3.877)	3.205 (3.600)	4.095 (3.466)	3.284 (3.186)	6.628 (3.939)	4.435 (3.826)	
- Median (Q1, Q3)	3.000 (1.000, 7.000)	2.000 (0.000, 5.000)	4.000 (1.000, 6.000)	2.000 (0.000, 5.000)	7.000 (3.750, 9.000)	4.000 (1.000, 7.000)	
- Min - Max	0.000 - 15.000	0.000 - 14.000	0.000 - 14.000	0.000 - 14.000	0.000 - 15.000	0.000 - 15.000	
T22							< 0.001
- Mean (SD)	45.275 (37.268)	51.471 (38.613)	24.214 (17.433)	57.712 (40.401)	21.627 (18.017)	41.490 (35.953)	
- Median (Q1, Q3)	30.000 (18.000, 100.000)	36.000 (18.000, 100.000)	22.000 (11.000, 34.250)	50.500 (17.750, 100.000)	16.000 (9.000, 31.000)	27.000 (12.500, 70.500)	
- Min - Max	2.000 - 100.000	5.000 - 100.000	4.000 - 100.000	2.000 - 100.000	1.000 - 100.000	1.000 - 100.000	
type							< 0.001
- OTHERABI	11 (6.0%)	0 (0.0%)	185 (78.4%)	15 (5.4%)	12 (5.6%)	223 (20.1%)	
- STROKE	0 (0.0%)	11 (5.7%)	24 (10.2%)	7 (2.5%)	199 (92.1%)	241 (21.8%)	
- TBI	172 (94.0%)	181 (94.3%)	27 (11.4%)	258 (92.1%)	5 (2.3%)	643 (58.1%)	
studies							0.128
- primary	86 (47.0%)	101 (52.6%)	112 (47.5%)	136 (48.6%)	83 (38.4%)	518 (46.8%)	
- secondary	60 (32.8%)	58 (30.2%)	76 (32.2%)	96 (34.3%)	75 (34.7%)	365 (33.0%)	
- tertiary	37 (20.2%)	33 (17.2%)	48 (20.3%)	48 (17.1%)	58 (26.9%)	224 (20.2%)	
Age							< 0.001
- >55	25 (13.7%)	40 (20.8%)	72 (30.5%)	45 (16.1%)	78 (36.1%)	260 (23.5%)	
- 17-30	76 (41.5%)	58 (30.2%)	43 (18.2%)	98 (35.0%)	6 (2.8%)	281 (25.4%)	
- 31-55	82 (44.8%)	94 (49.0%)	121 (51.3%)	137 (48.9%)	132 (61.1%)	566 (51.1%)	
Agecont							< 0.001
- Mean (SD)	37.635 (15.118)	41.394 (14.567)	46.829 (14.843)	39.732 (14.958)	51.842 (9.268)	43.549 (14.840)	
- Median (Q1, Q3)	35.016 (24.510, 50.163)	41.470 (28.323, 52.723)	47.974 (35.960, 57.986)	38.275 (27.227, 50.344)	52.767 (46.577, 58.429)	44.466 (30.875, 55.107)	
- Min - Max	16.781 - 74.901	16.811 - 76.241	17.216 - 78.636	17.449 - 80.384	21.447 - 74.501	16.781 - 80.384	
TSOcont							< 0.001
- Mean (SD)	71.262 (86.681)	125.766 (25.545)	139.504 (82.492)	65.446 (12.727)	70.148 (64.051)	93.575 (68.215)	
- Median (Q1, Q3)	37.000 (29.500, 43.500)	121.000 (104.000, 146.250)	115.500 (76.750, 197.250)	66.000 (54.000, 75.000)	48.000 (34.750, 76.000)	73.000 (46.000, 117.500)	
- Min - Max	8.000 - 364.000	91.000 - 180.000	11.000 - 364.000	46.000 - 90.000	6.000 - 354.000	6.000 - 364.000	
TSO							< 0.001
- 0-45	151 (82.5%)	0 (0.0%)	16 (6.8%)	0 (0.0%)	103 (47.7%)	270 (24.4%)	
- 181-364	32 (17.5%)	0 (0.0%)	72 (30.5%)	0 (0.0%)	20 (9.3%)	124 (11.2%)	
- 46-90	0 (0.0%)	0 (0.0%)	55 (23.3%)	280 (100.0%)	75 (34.7%)	410 (37.0%)	
- 91-180	0 (0.0%)	192 (100.0%)	93 (39.4%)	0 (0.0%)	18 (8.3%)	303 (27.4%)	

<u>*Optimal Clusters' tSNE*</u>

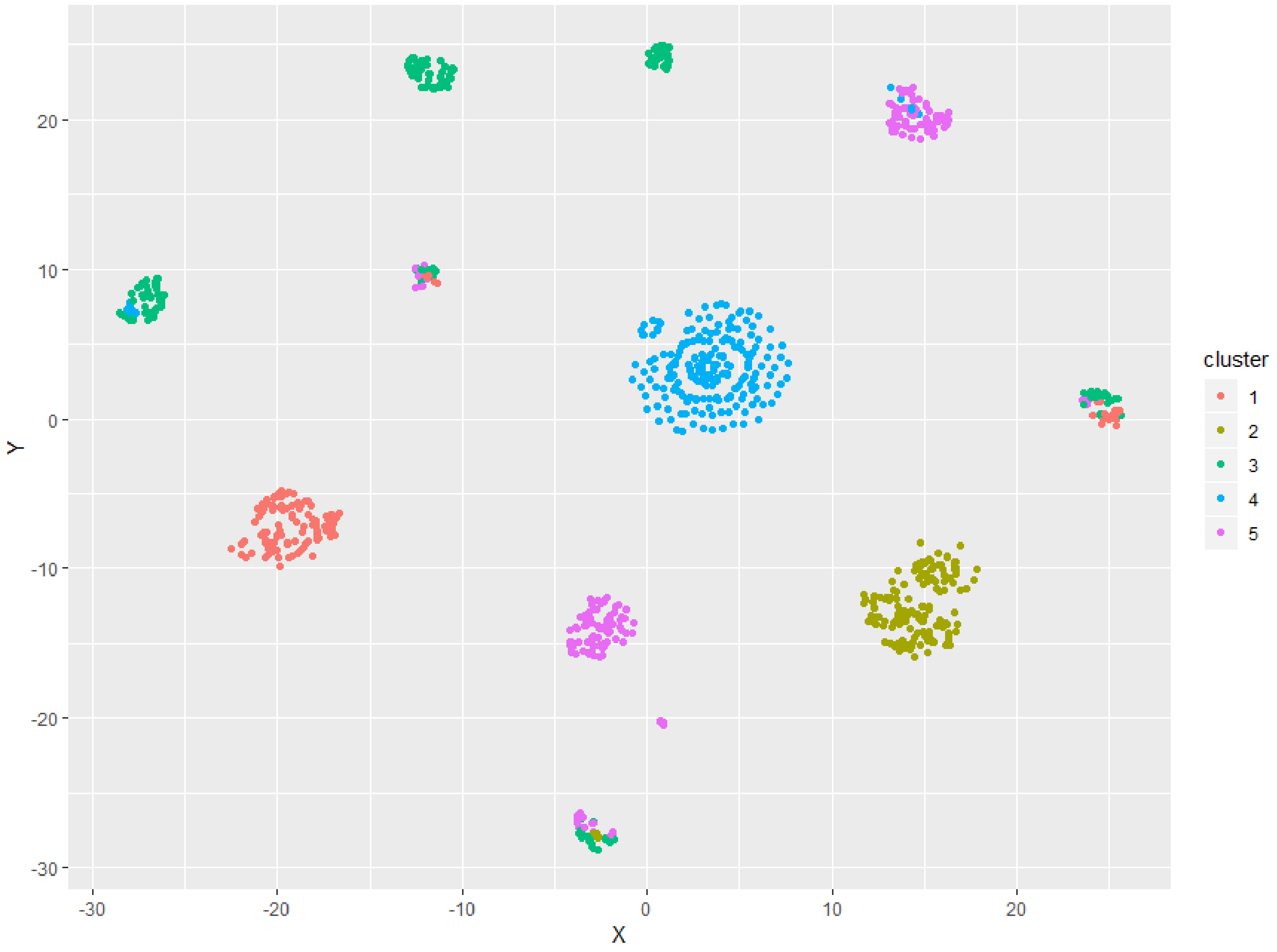

Injury, studies and age

Number of clusters

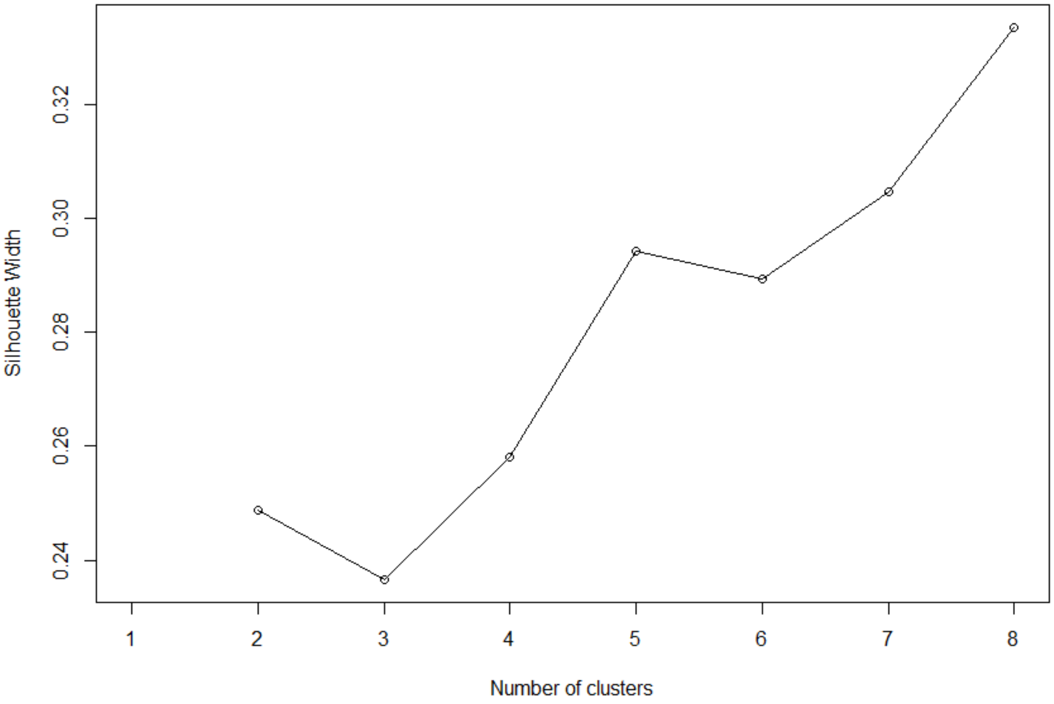

Optimal Clusters' Silhouette (k=5)

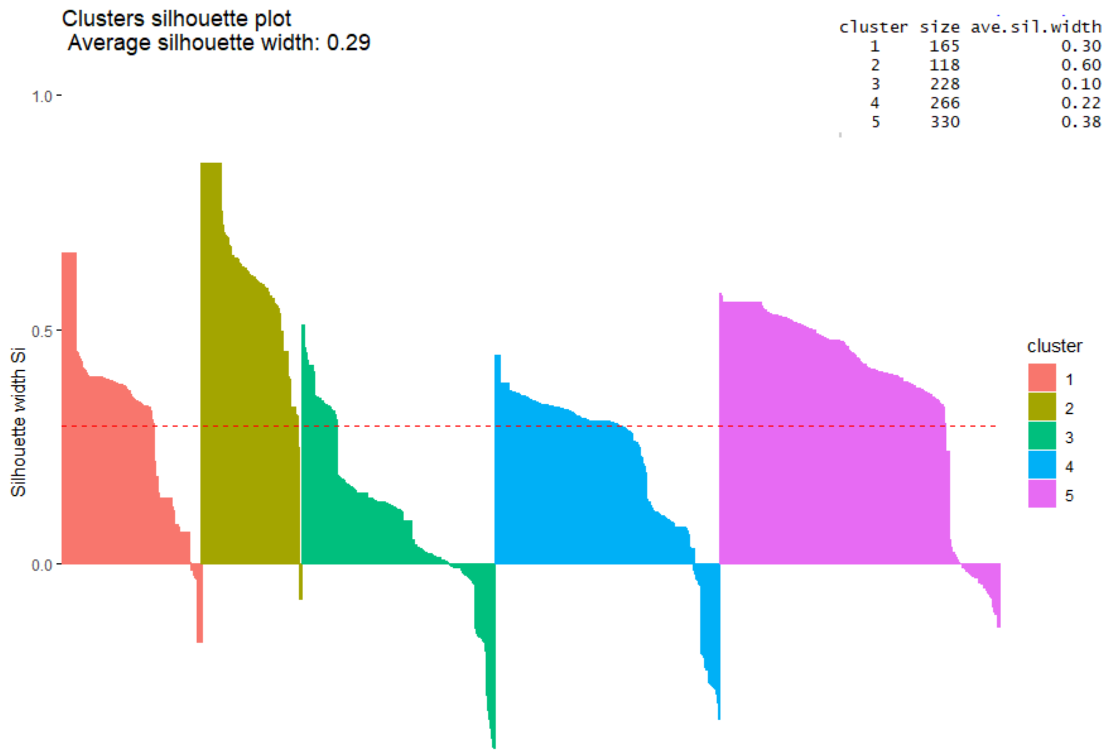

Optimal Clusters' tSNE

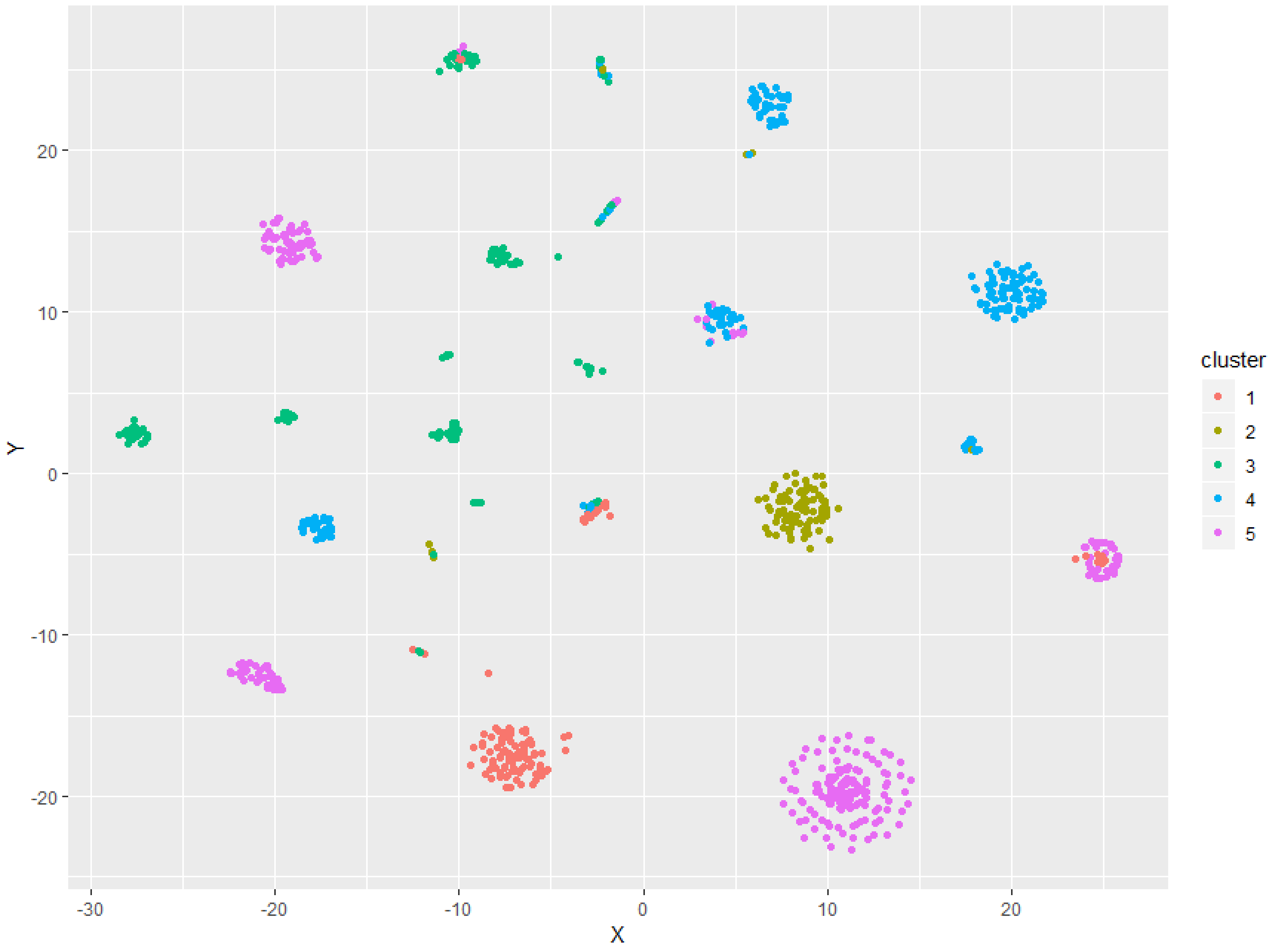

Injury, studies and time since injury

Number of clusters

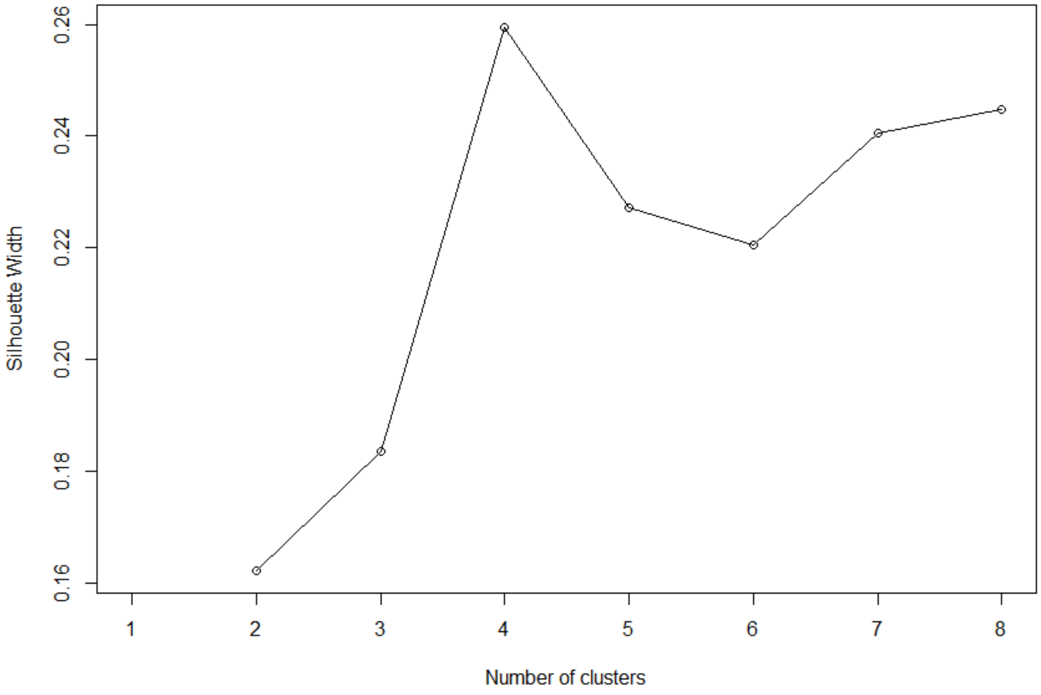

Optimal Clusters' Silhouette (k=4)

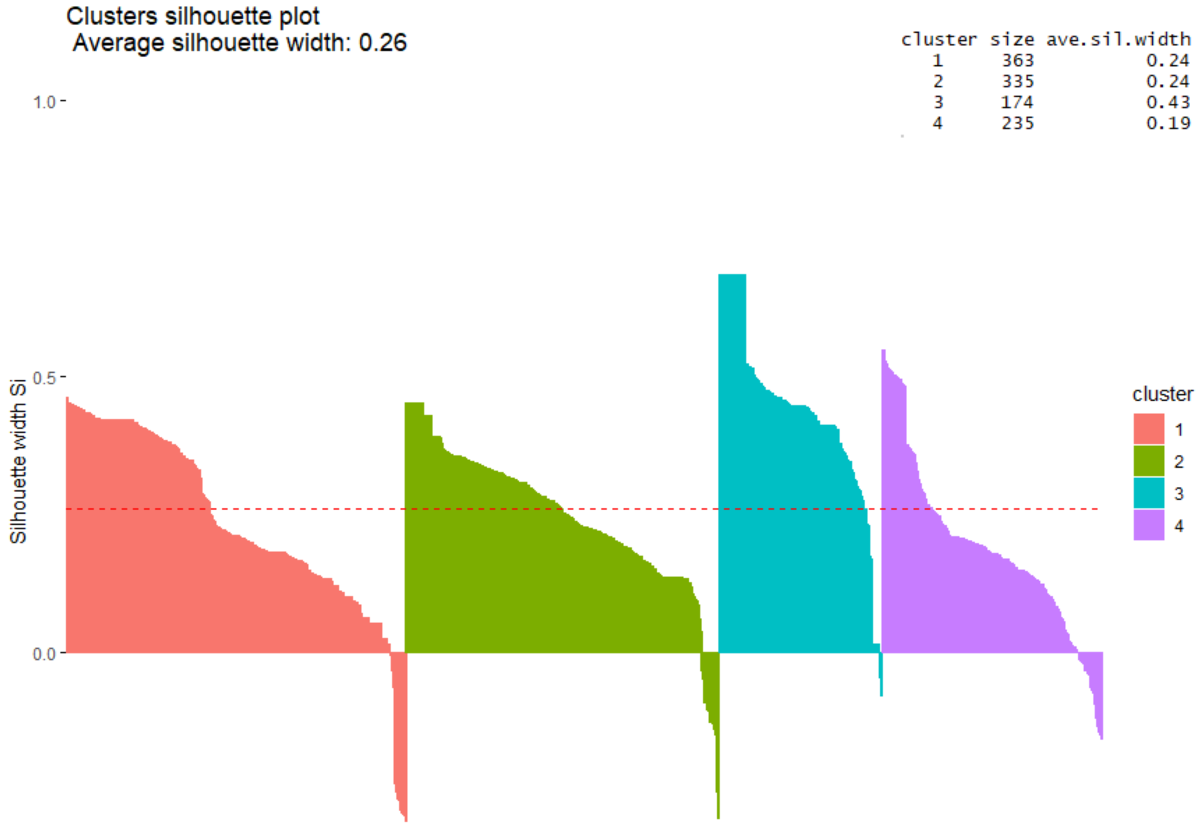

Optimal Clusters' tSNE

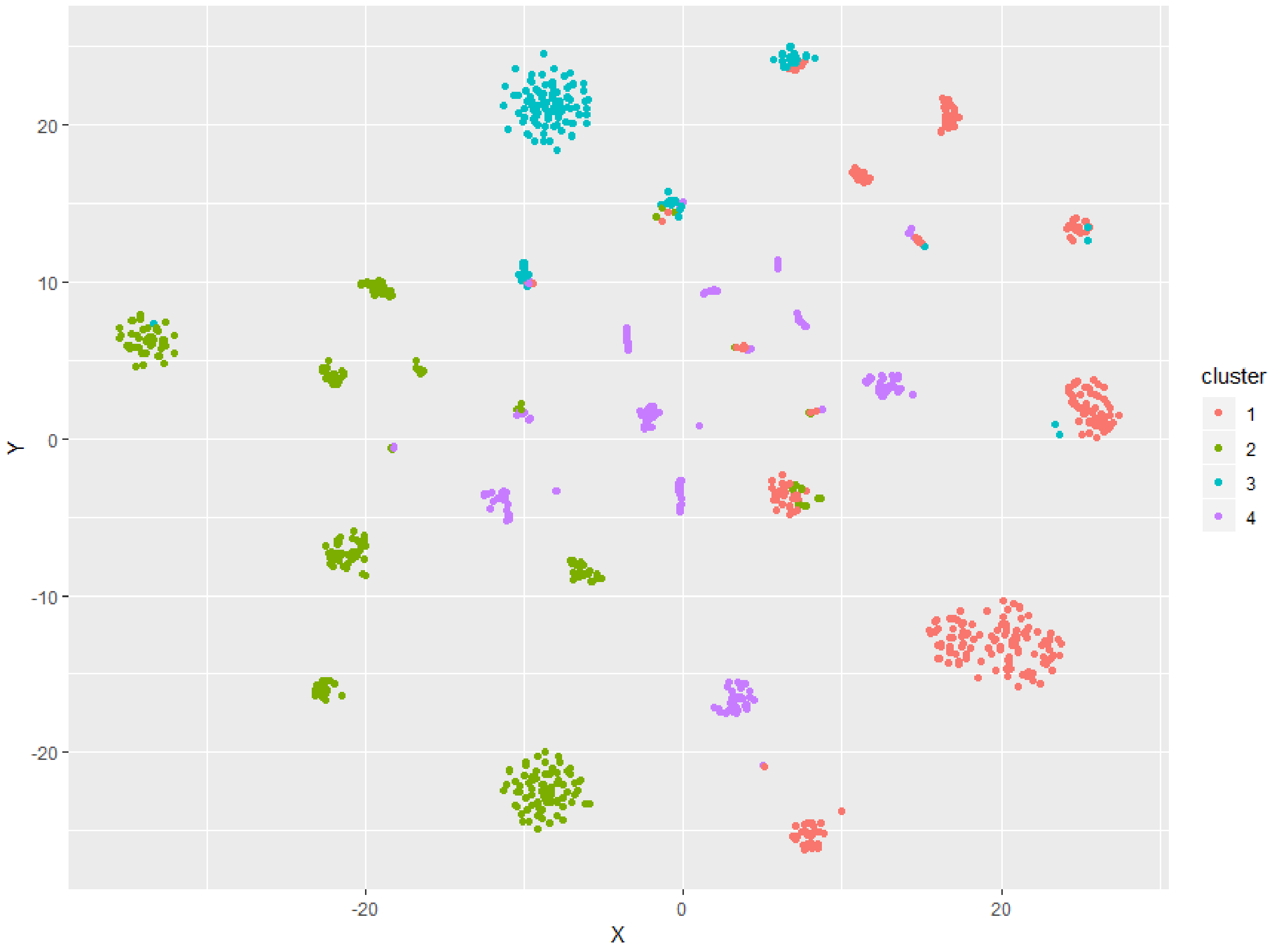

Injury, time since injury and age

<u>Number of clusters</u>

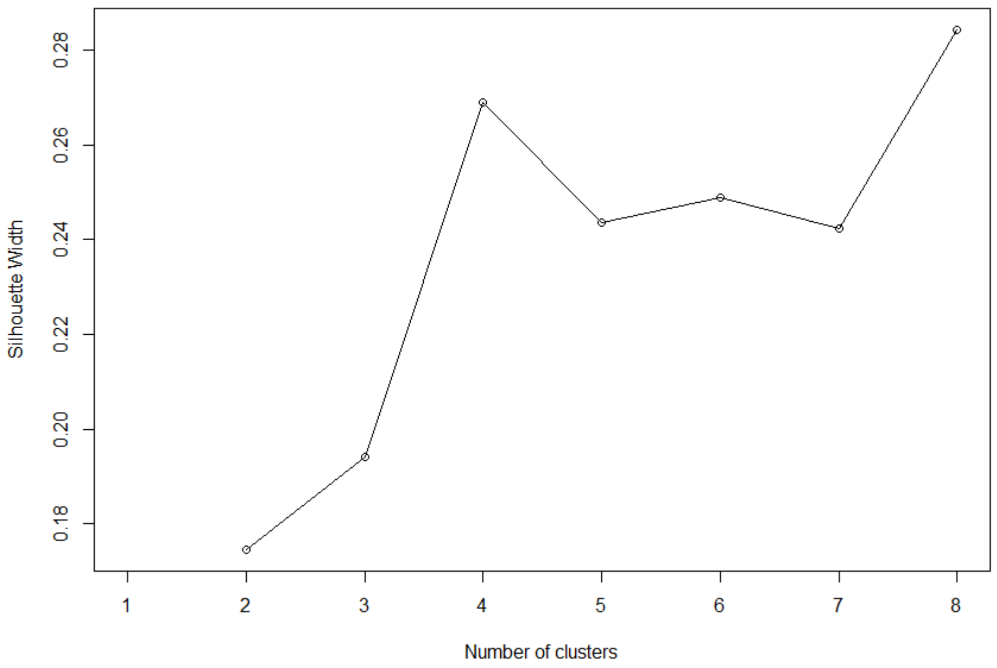

<u>Optimal Clusters' Silhouette (k=4)</u>

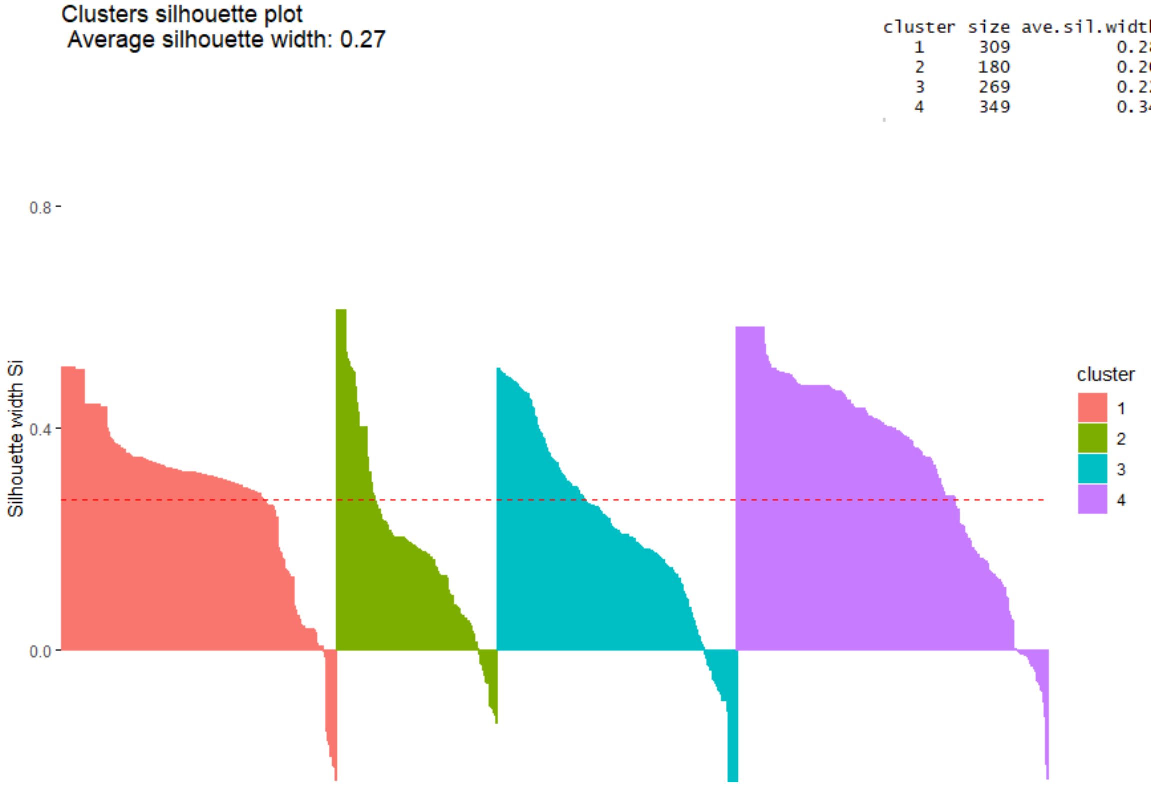

cluster	size	ave.sil.width
1	309	0.28
2	180	0.20
3	269	0.22
4	349	0.34

<u>Optimal Clusters' tSNE</u>

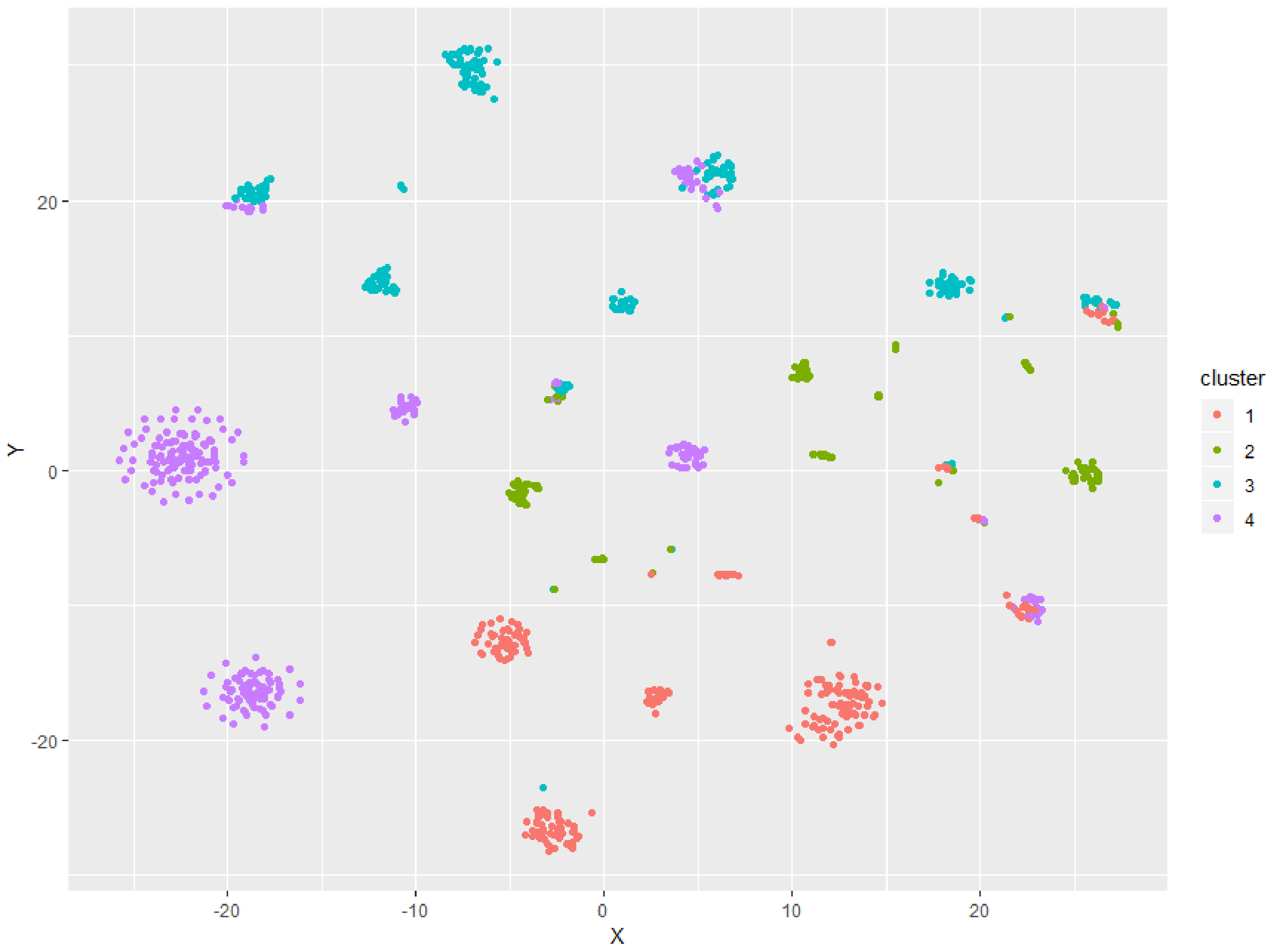

Injury, studies, time since injury and age

Number of clusters

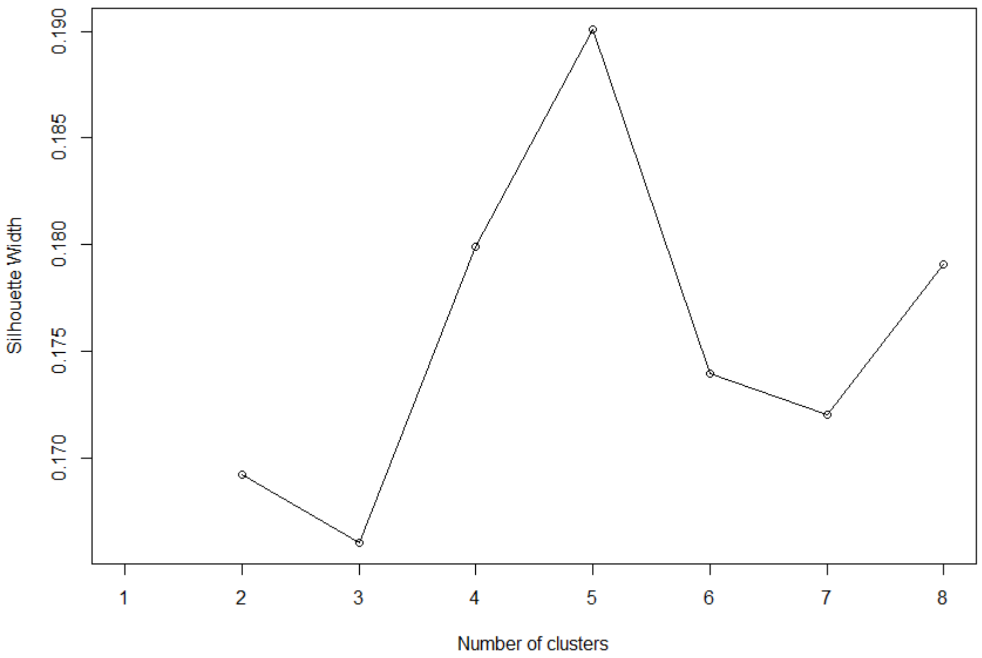

Optimal Clusters' Silhouette (k=5)

Clusters silhouette plot
Average silhouette width: 0.19

cluster	size	ave.sil.width
1	225	0.19
2	246	0.17
3	180	0.13
4	288	0.23
5	168	0.23

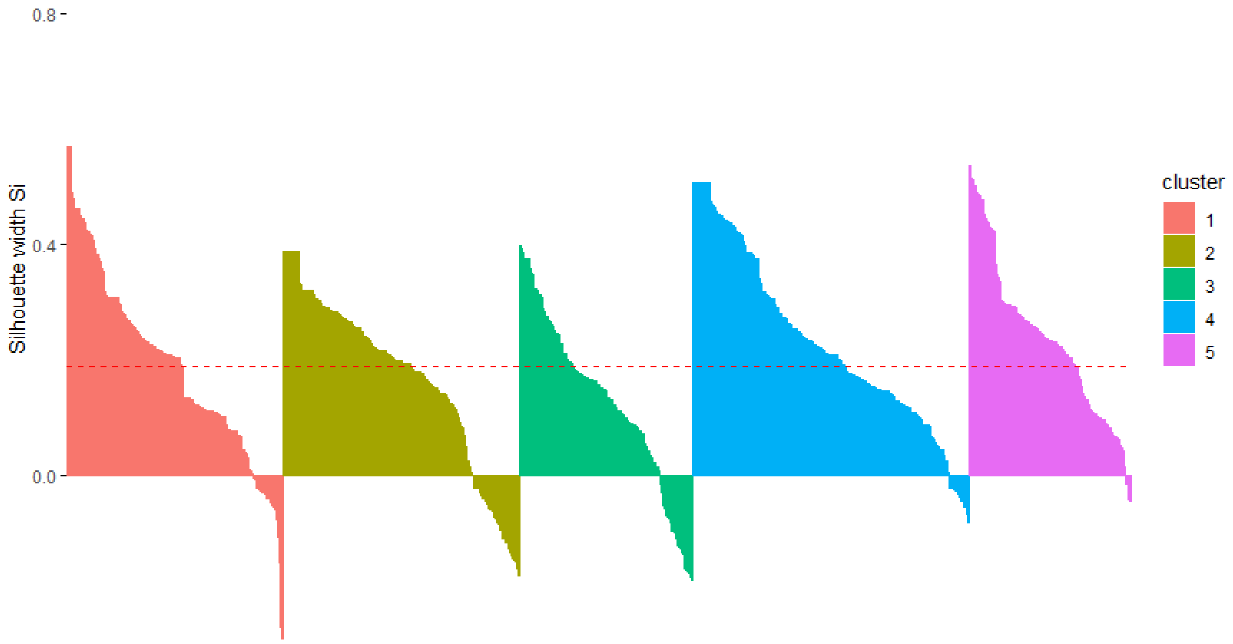

Optimal Clusters' tSNE

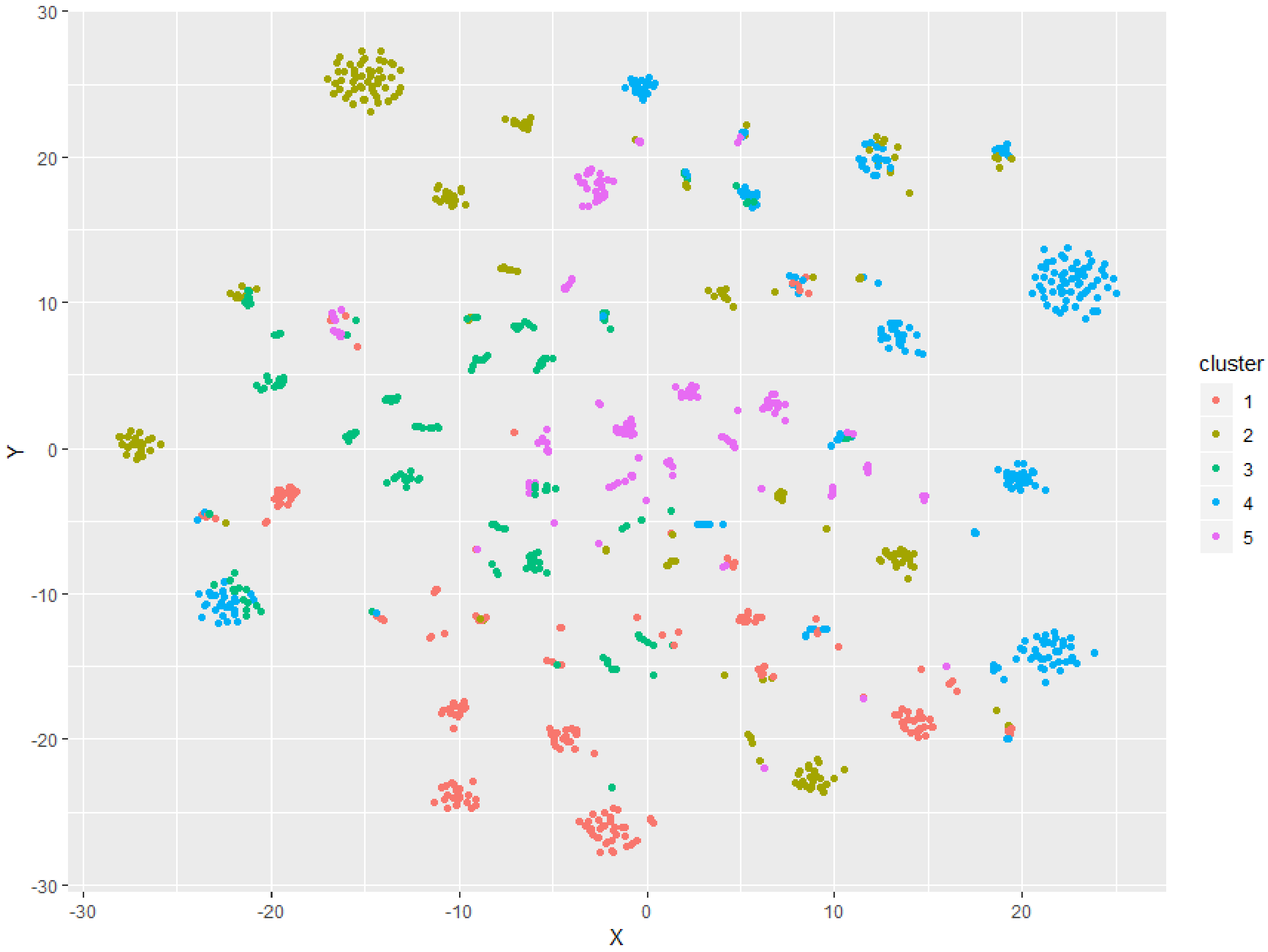

Studies

Only studies

Number of clusters

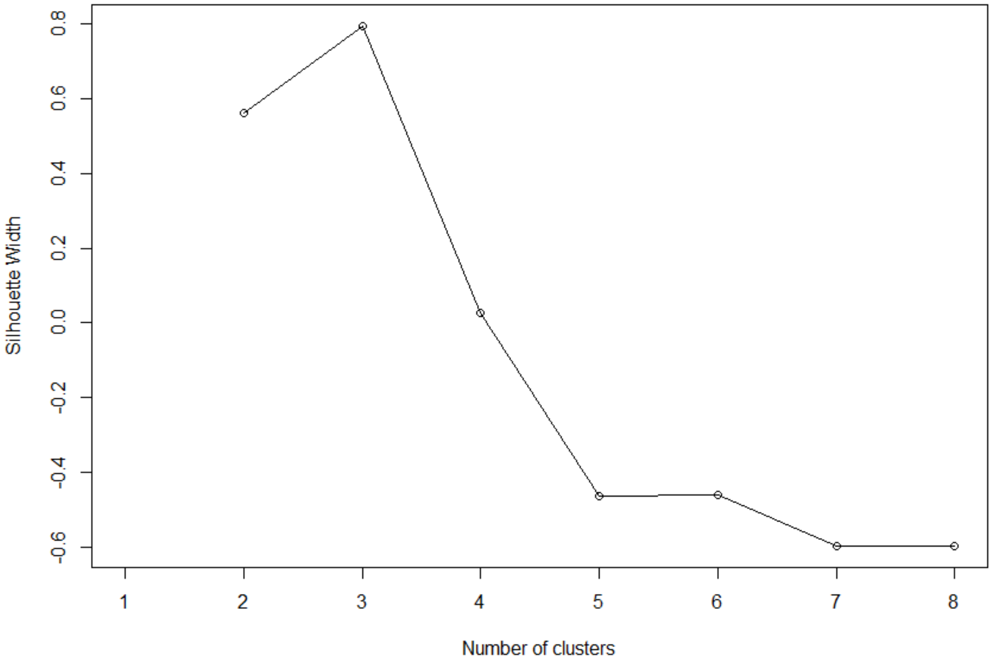

Optimal Clusters' Silhouette (k=3)

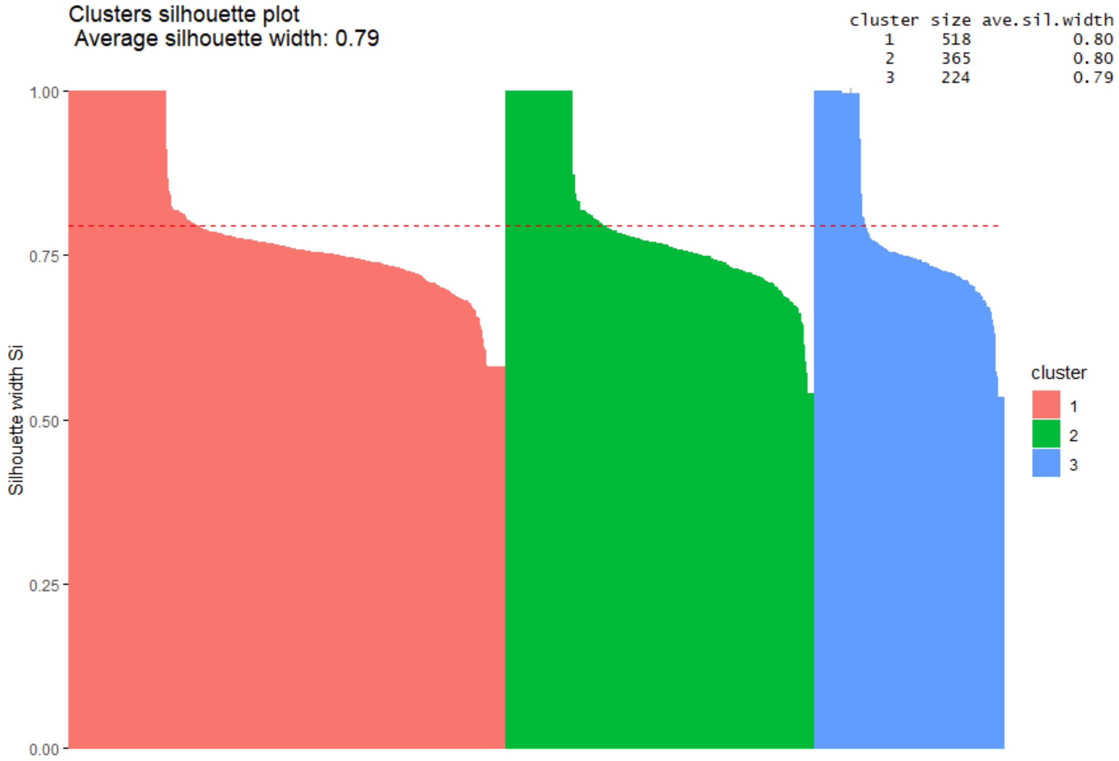

Optimal Clusters' Descriptive statistics

	1 (N=518)	2 (N=365)	3 (N=224)	Total (N=1107)	p value
T3					0.294
- Mean (SD)	20.174 (6.088)	20.608 (5.339)	20.170 (6.134)	20.318 (5.855)	
- Median (Q1, Q3)	23.000 (21.000, 23.000)	23.000 (21.750, 23.000)	23.000 (22.000, 23.000)	23.000 (22.000, 23.000)	
- Min - Max	0.000 - 23.000	0.000 - 23.000	0.000 - 23.000	0.000 - 23.000	
T5					0.020
- Mean (SD)	108.310 (89.298)	96.820 (79.674)	90.525 (79.438)	100.903 (84.446)	
- Median (Q1, Q3)	71.500 (50.000, 135.250)	70.000 (45.000, 115.000)	61.000 (39.000, 100.000)	68.000 (46.000, 119.000)	
- Min - Max	14.000 - 480.000	11.000 - 476.000	18.000 - 300.000	11.000 - 480.000	
T14					< 0.001
- Mean (SD)	22.175 (11.044)	25.074 (12.421)	27.516 (12.738)	24.217 (12.033)	
- Median (Q1, Q3)	20.000 (12.000, 28.000)	24.000 (16.000, 32.500)	28.000 (20.000, 34.250)	24.000 (14.750, 31.000)	
- Min - Max	1.000 - 59.000	1.000 - 68.000	1.000 - 56.000	1.000 - 68.000	
T15					< 0.001
- Mean (SD)	3.566 (0.948)	3.800 (0.940)	4.125 (1.120)	3.758 (1.003)	
- Median (Q1, Q3)	4.000 (3.000, 4.000)	4.000 (3.000, 4.000)	4.000 (3.000, 5.000)	4.000 (3.000, 4.000)	
- Min - Max	2.000 - 6.000	1.000 - 6.000	2.000 - 7.000	1.000 - 7.000	
T18					< 0.001
- Mean (SD)	4.000 (3.613)	4.428 (3.847)	5.472 (4.096)	4.435 (3.826)	
- Median (Q1, Q3)	3.000 (1.000, 7.000)	4.000 (1.000, 7.000)	5.000 (2.000, 9.000)	4.000 (1.000, 7.000)	
- Min - Max	0.000 - 15.000	0.000 - 14.000	0.000 - 15.000	0.000 - 15.000	
T22					< 0.001
- Mean (SD)	49.399 (37.047)	38.359 (35.175)	31.747 (32.163)	41.490 (35.953)	
- Median (Q1, Q3)	33.500 (18.000, 100.000)	24.500 (12.000, 49.250)	22.000 (9.000, 35.500)	27.000 (12.500, 70.500)	
- Min - Max	2.000 - 100.000	3.000 - 100.000	1.000 - 100.000	1.000 - 100.000	
type					0.053
- OTHERABI	97 (18.7%)	75 (20.5%)	51 (22.8%)	223 (20.1%)	
- STROKE	101 (19.5%)	79 (21.6%)	61 (27.2%)	241 (21.8%)	
- TBI	320 (61.8%)	211 (57.8%)	112 (50.0%)	643 (58.1%)	
studies					< 0.001
- primary	518 (100.0%)	0 (0.0%)	0 (0.0%)	518 (46.8%)	
- secondary	0 (0.0%)	365 (100.0%)	0 (0.0%)	365 (33.0%)	
- tertiary	0 (0.0%)	0 (0.0%)	224 (100.0%)	224 (20.2%)	
Age					0.002
- >55	128 (24.7%)	67 (18.4%)	65 (29.0%)	260 (23.5%)	
- 17-30	119 (23.0%)	116 (31.8%)	46 (20.5%)	281 (25.4%)	
- 31-55	271 (52.3%)	182 (49.9%)	113 (50.4%)	566 (51.1%)	
Agecont					< 0.001
- Mean (SD)	44.440 (14.375)	40.987 (15.624)	45.666 (14.055)	43.549 (14.840)	
- Median (Q1, Q3)	46.401 (33.149, 55.823)	41.274 (27.159, 52.504)	46.342 (33.068, 57.922)	44.466 (30.875, 55.107)	
- Min - Max	16.781 - 76.241	16.811 - 80.384	20.082 - 76.904	16.781 - 80.384	
TSOcont					0.033
- Mean (SD)	94.402 (65.726)	96.252 (70.263)	87.304 (70.371)	93.575 (68.215)	
- Median (Q1, Q3)	75.000 (47.250, 120.000)	74.000 (48.000, 121.000)	66.000 (38.000, 105.250)	73.000 (46.000, 117.500)	
- Min - Max	9.000 - 364.000	6.000 - 364.000	12.000 - 351.000	6.000 - 364.000	
TSO					0.105
- 0-45	118 (22.8%)	82 (22.5%)	70 (31.2%)	270 (24.4%)	
- 181-364	54 (10.4%)	49 (13.4%)	21 (9.4%)	124 (11.2%)	
- 46-90	193 (37.3%)	141 (38.6%)	76 (33.9%)	410 (37.0%)	
- 91-180	153 (29.5%)	93 (25.5%)	57 (25.4%)	303 (27.4%)	

Optimal Clusters' tSNE

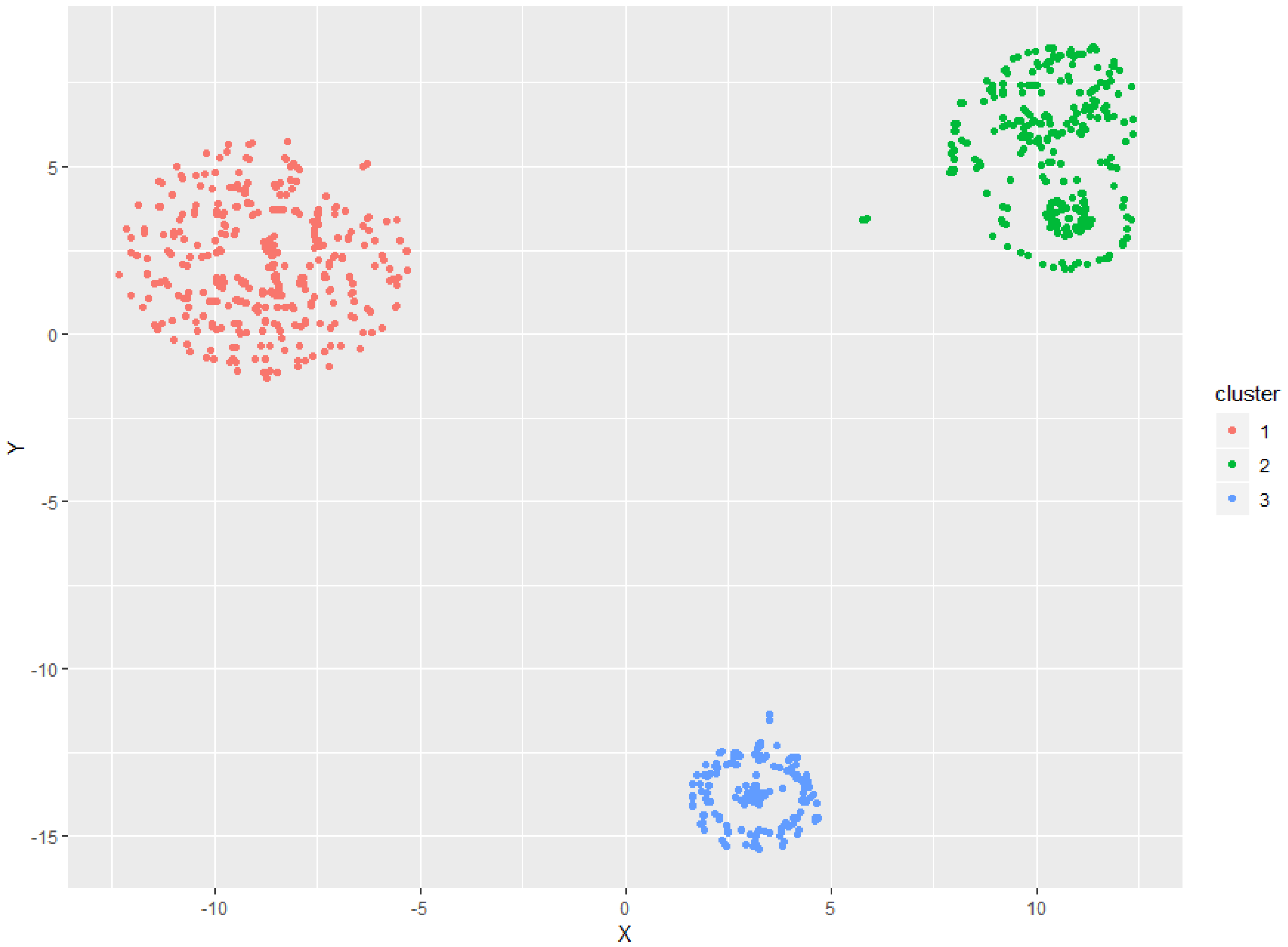

Studies and age

Number of clusters

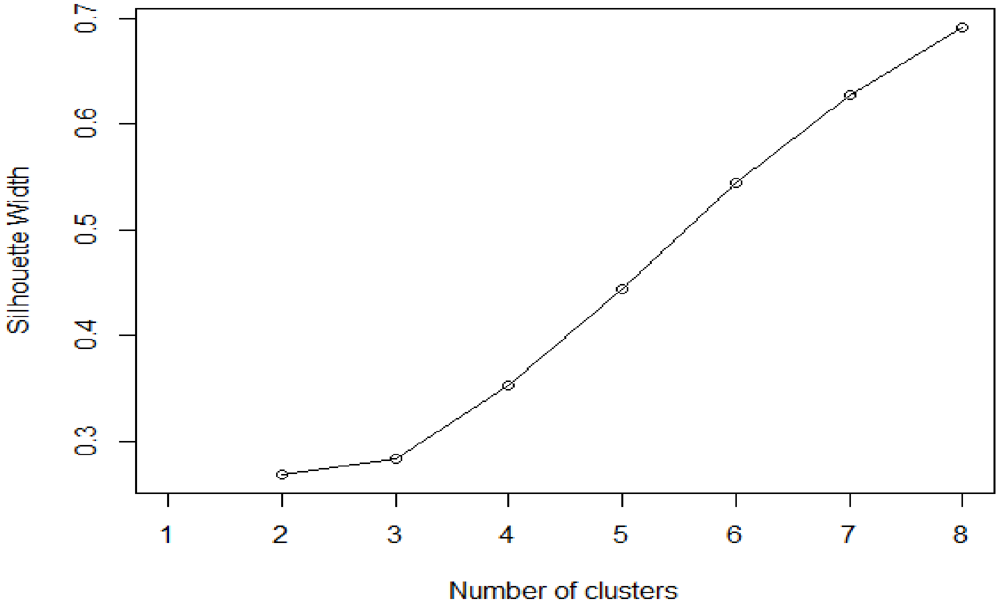

Optimal Clusters' Silhouette (k=6)

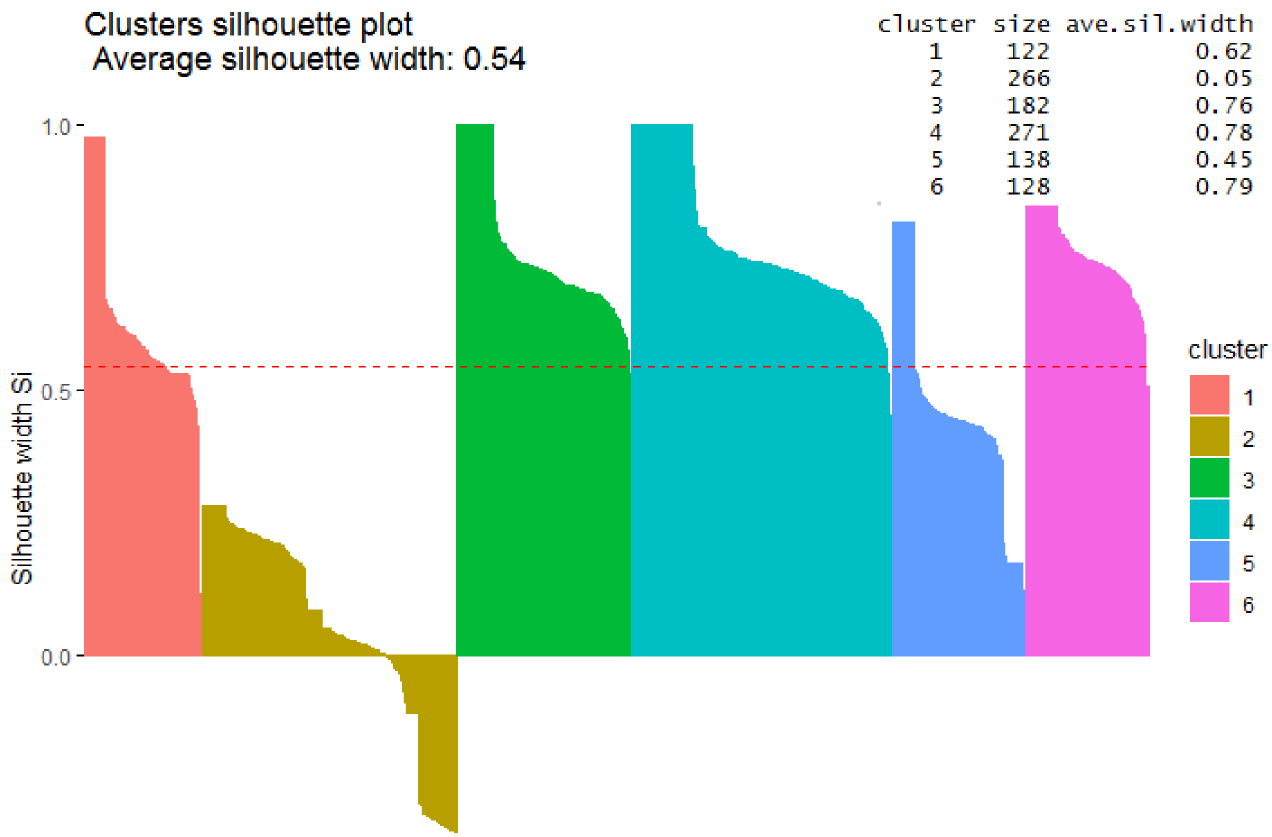

Optimal Clusters' tSNE

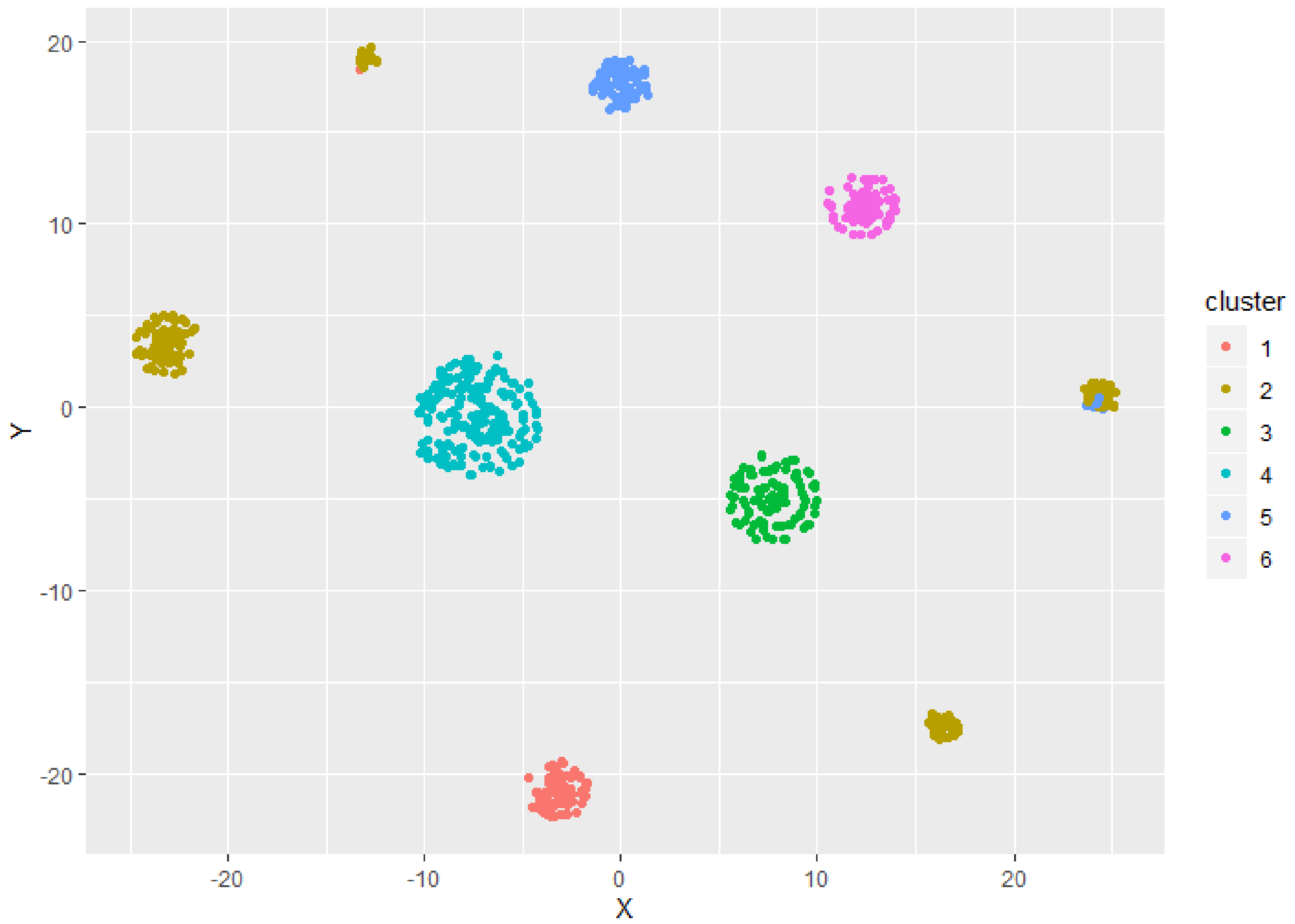

Studies and time since injury

Number of clusters

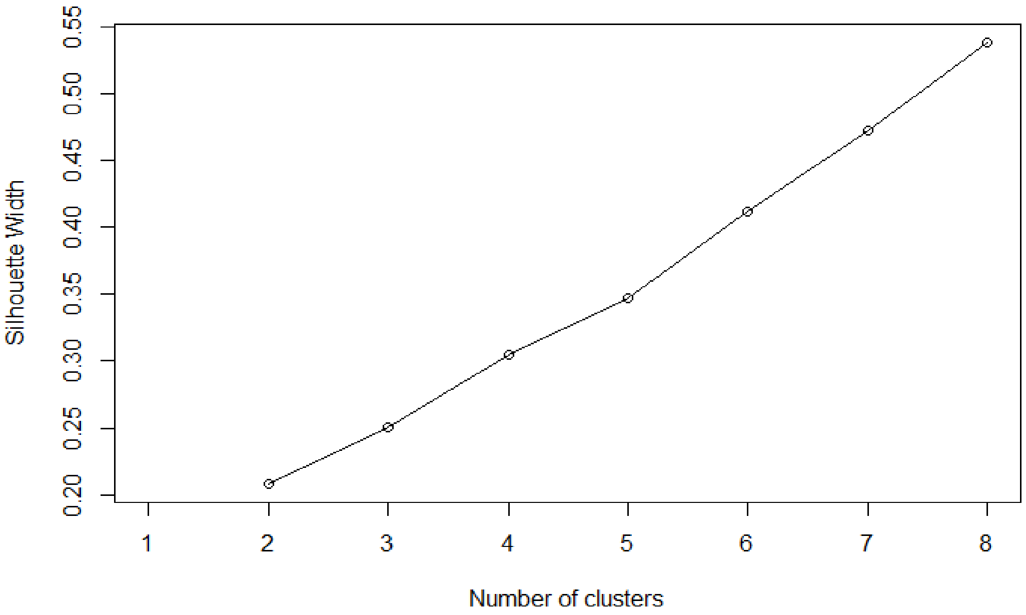

Optimal Clusters' Silhouette (k=8)

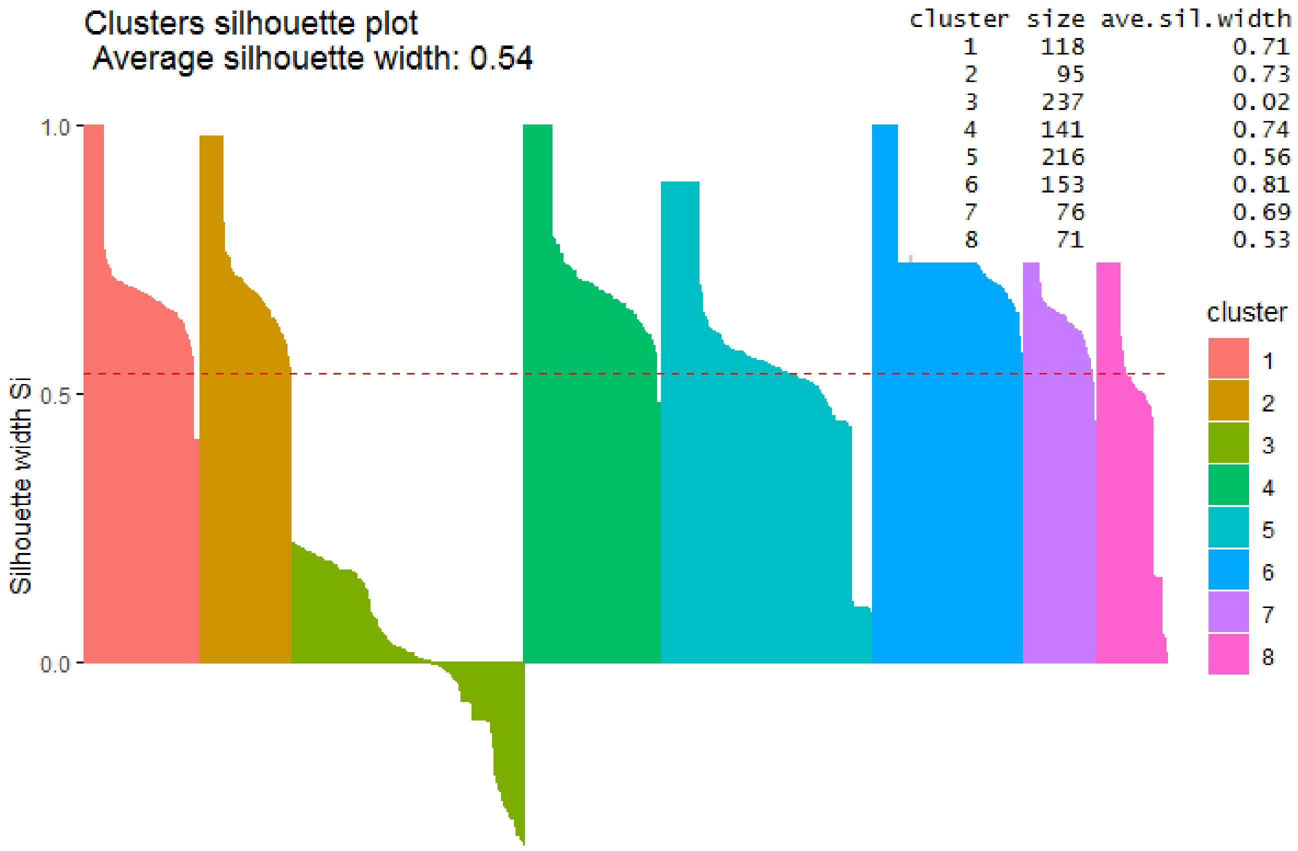

Optimal Clusters' tSNE

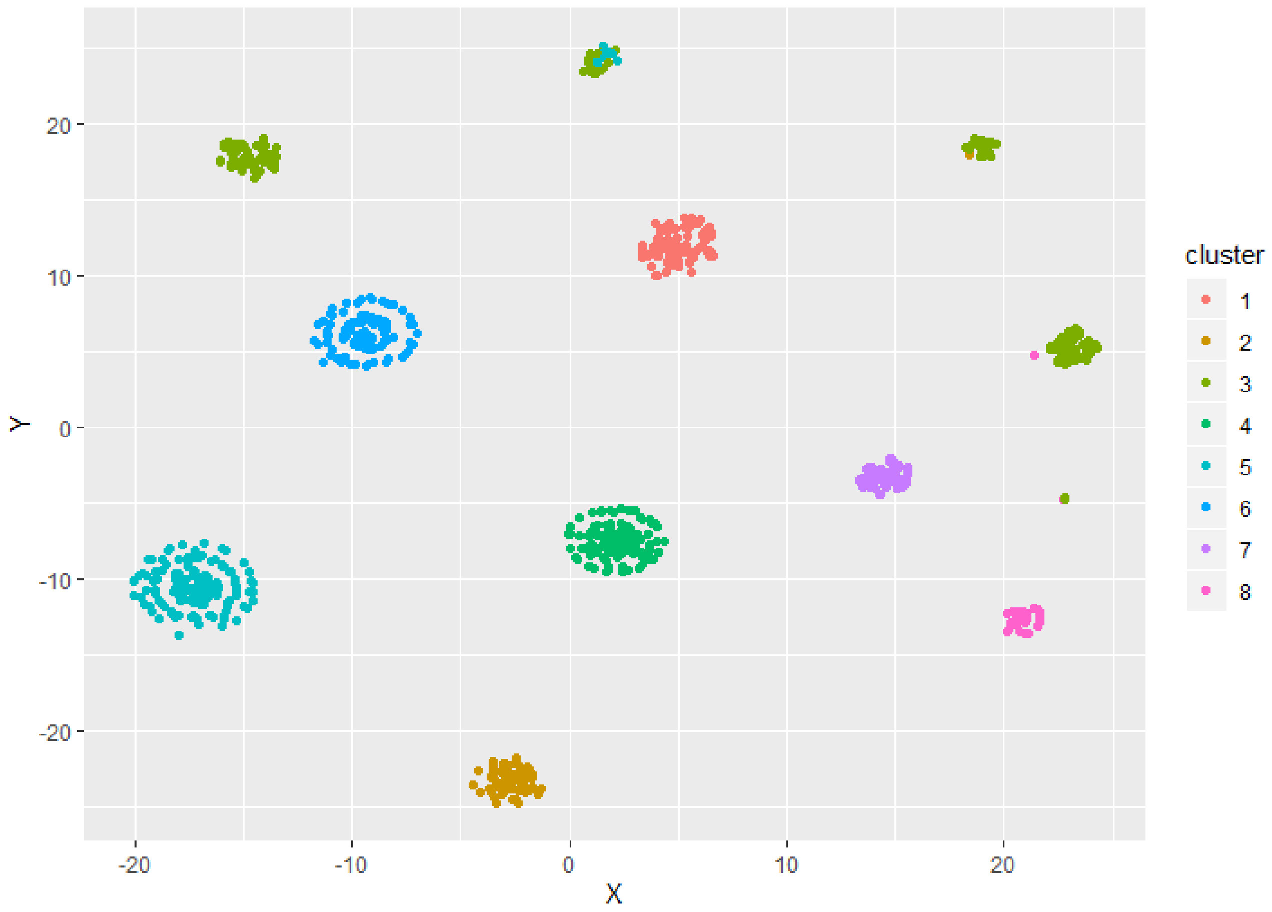

Studies, time since injury and age

Number of clusters

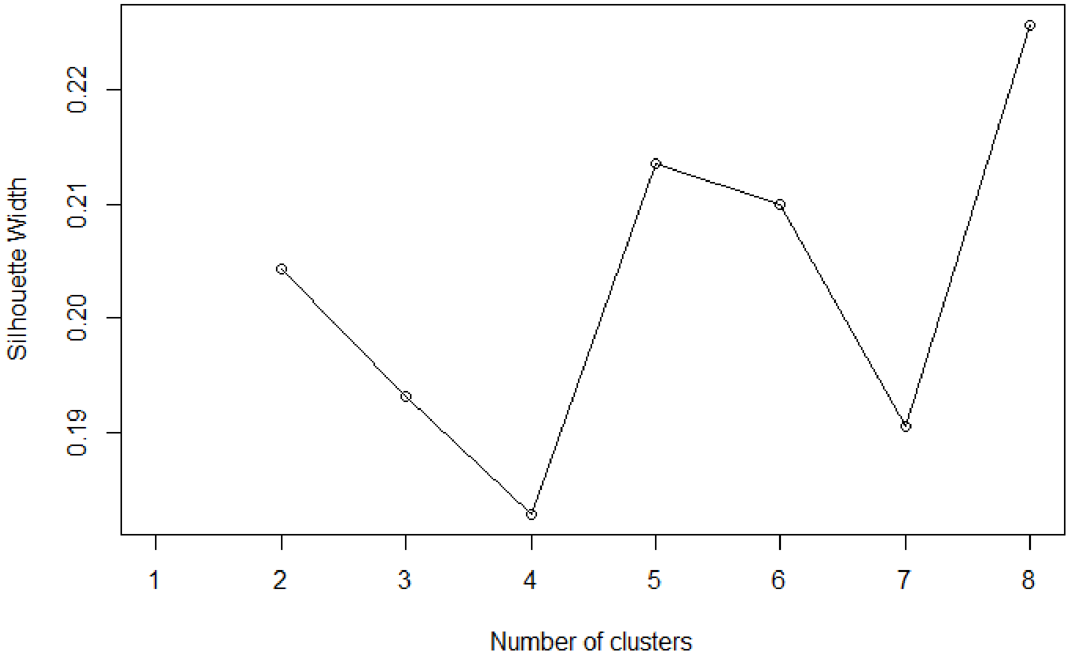

Optimal Clusters' Silhouette (k=5)

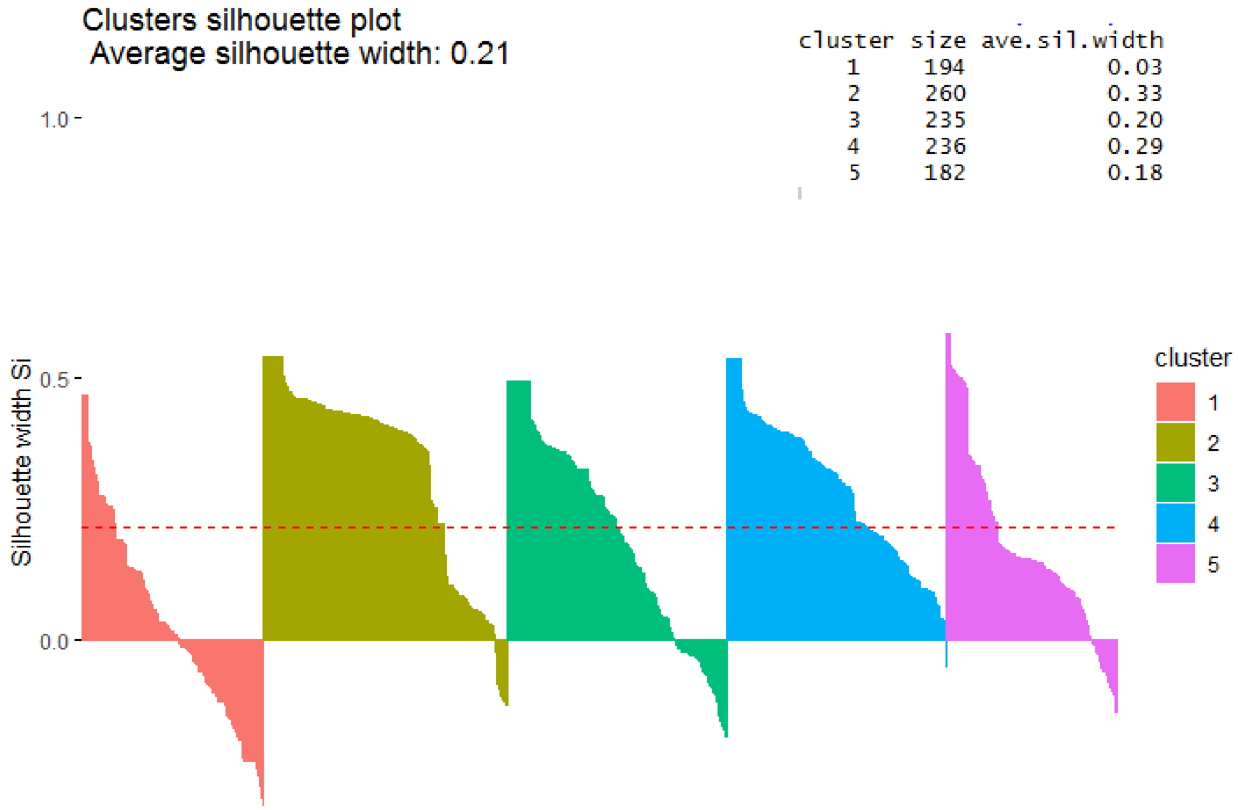

cluster	size	ave.sil.width
1	194	0.03
2	260	0.33
3	235	0.20
4	236	0.29
5	182	0.18

Optimal Clusters' tSNE

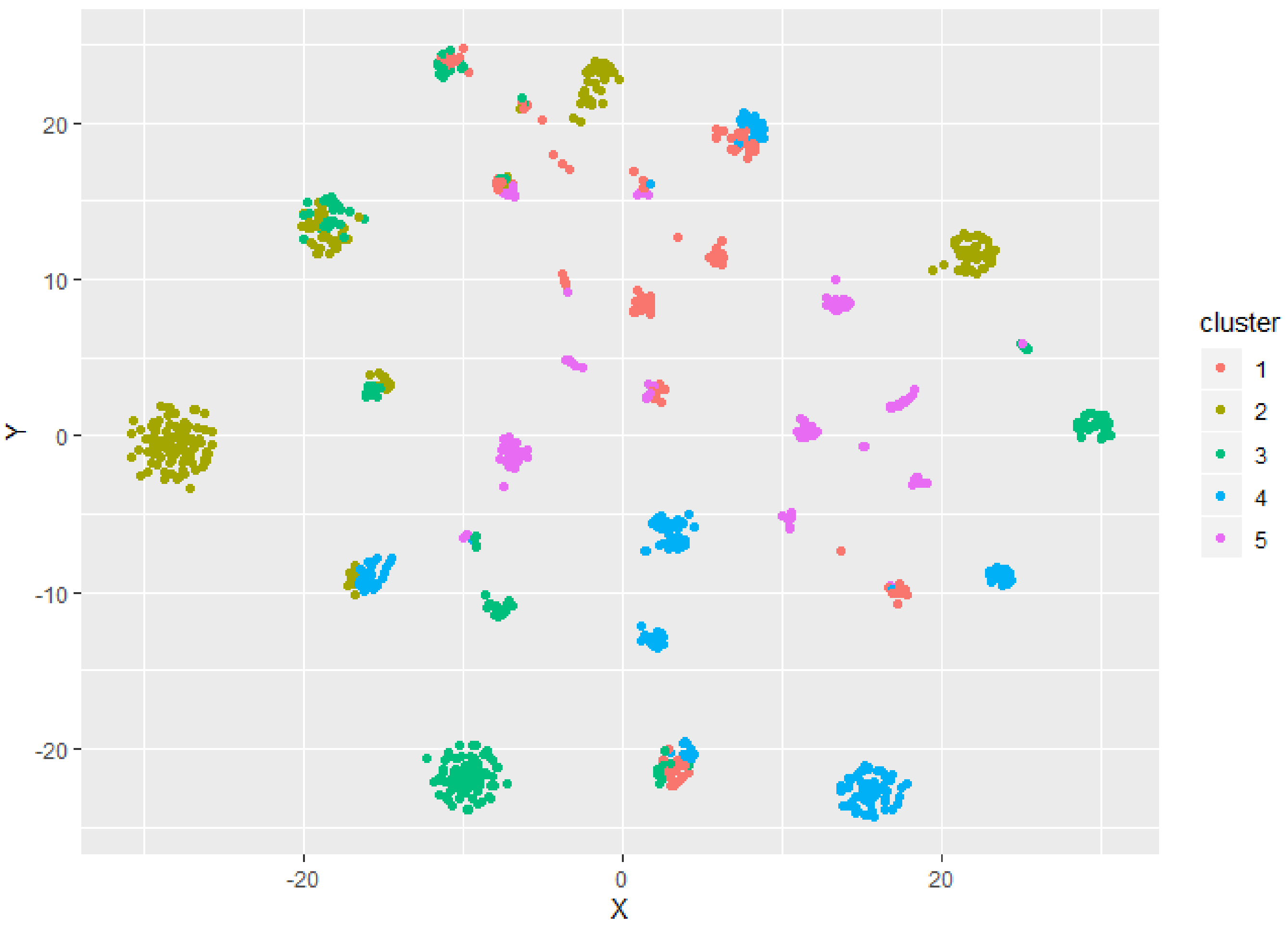

Age

Only age

Number of clusters

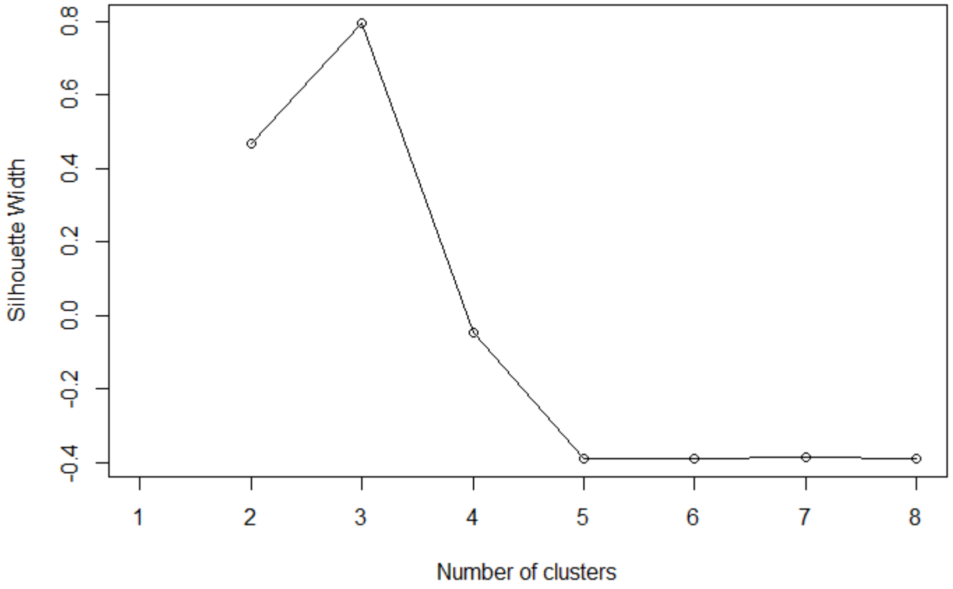

Optimal Clusters' Silhouette (k=3)

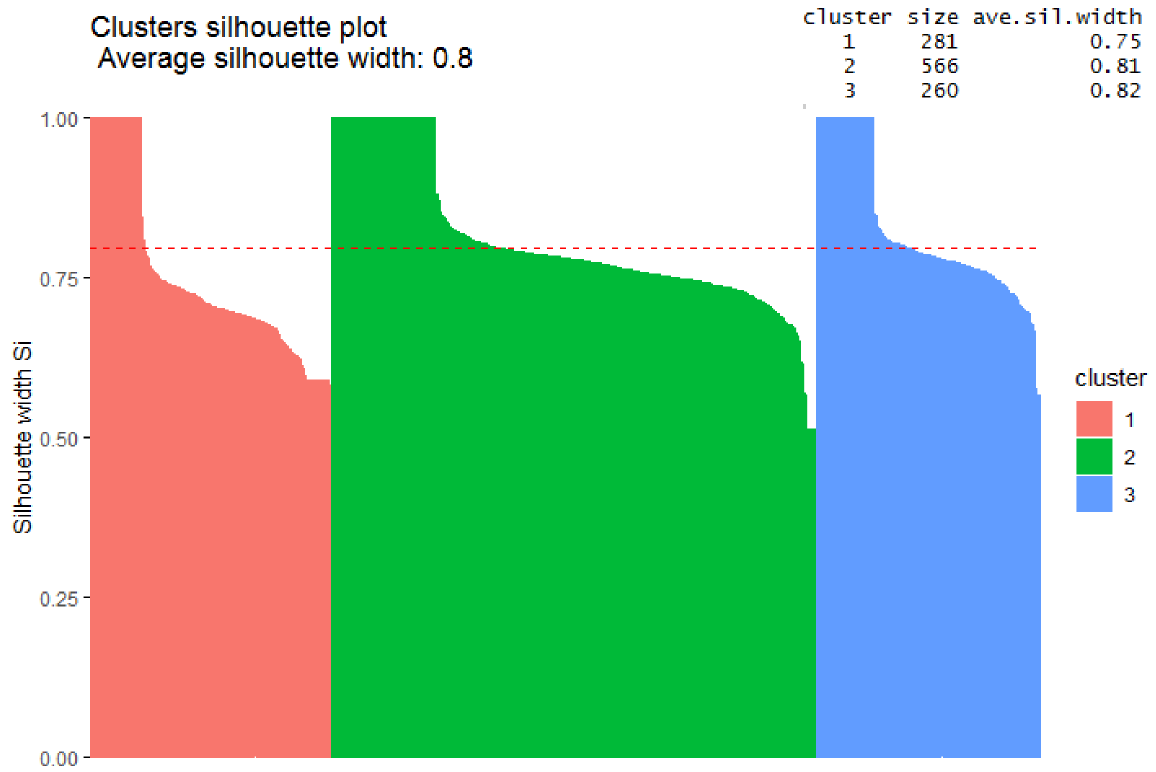

Optimal Clusters' Descriptive statistics

	1 (N=281)	2 (N=566)	3 (N=260)	Total (N=1107)	p value
T3					0.071
- Mean (SD)	19.067 (7.562)	20.796 (5.114)	20.660 (4.954)	20.318 (5.855)	
- Median (Q1, Q3)	23.000 (21.000, 23.000)	23.000 (22.000, 23.000)	23.000 (22.000, 23.000)	23.000 (22.000, 23.000)	
- Min - Max	0.000 - 23.000	0.000 - 23.000	0.000 - 23.000	0.000 - 23.000	
T5					< 0.001
- Mean (SD)	121.467 (98.502)	87.244 (75.907)	107.299 (78.108)	100.903 (84.446)	
- Median (Q1, Q3)	77.000 (49.000, 155.000)	63.000 (43.000, 90.000)	84.500 (53.000, 140.000)	68.000 (46.000, 119.000)	
- Min - Max	15.000 - 340.000	11.000 - 476.000	20.000 - 480.000	11.000 - 480.000	
T14					< 0.001
- Mean (SD)	25.794 (13.123)	24.974 (12.043)	20.699 (9.996)	24.217 (12.033)	
- Median (Q1, Q3)	24.000 (16.000, 34.000)	24.000 (16.000, 32.000)	20.000 (12.000, 28.000)	24.000 (14.750, 31.000)	
- Min - Max	1.000 - 68.000	1.000 - 59.000	1.000 - 51.000	1.000 - 68.000	
T15					0.231
- Mean (SD)	3.764 (1.047)	3.801 (0.951)	3.652 (1.069)	3.758 (1.003)	
- Median (Q1, Q3)	4.000 (3.000, 4.000)	4.000 (3.000, 4.000)	4.000 (3.000, 4.000)	4.000 (3.000, 4.000)	
- Min - Max	1.000 - 7.000	2.000 - 6.000	2.000 - 7.000	1.000 - 7.000	
T18					0.018
- Mean (SD)	3.995 (3.844)	4.784 (3.891)	4.143 (3.603)	4.435 (3.826)	
- Median (Q1, Q3)	3.000 (0.000, 6.000)	4.000 (1.000, 8.000)	4.000 (1.000, 7.000)	4.000 (1.000, 7.000)	
- Min - Max	0.000 - 14.000	0.000 - 15.000	0.000 - 14.000	0.000 - 15.000	
T22					0.012
- Mean (SD)	51.295 (39.787)	35.806 (33.038)	38.226 (31.789)	41.490 (35.953)	
- Median (Q1, Q3)	33.000 (13.500, 100.000)	23.500 (12.000, 43.250)	30.000 (12.000, 57.000)	27.000 (12.500, 70.500)	
- Min - Max	2.000 - 100.000	1.000 - 100.000	3.000 - 100.000	1.000 - 100.000	
type					< 0.001
- OTHERABI	33 (11.7%)	123 (21.7%)	67 (25.8%)	223 (20.1%)	
- STROKE	8 (2.8%)	142 (25.1%)	91 (35.0%)	241 (21.8%)	
- TBI	240 (85.4%)	301 (53.2%)	102 (39.2%)	643 (58.1%)	
studies					0.002
- primary	119 (42.3%)	271 (47.9%)	128 (49.2%)	518 (46.8%)	
- secondary	116 (41.3%)	182 (32.2%)	67 (25.8%)	365 (33.0%)	
- tertiary	46 (16.4%)	113 (20.0%)	65 (25.0%)	224 (20.2%)	
Age					< 0.001
- >55	0 (0.0%)	0 (0.0%)	260 (100.0%)	260 (23.5%)	
- 17-30	281 (100.0%)	0 (0.0%)	0 (0.0%)	281 (25.4%)	
- 31-55	0 (0.0%)	566 (100.0%)	0 (0.0%)	566 (51.1%)	
Agecont					< 0.001
- Mean (SD)	23.868 (4.184)	44.563 (6.959)	62.614 (5.332)	43.549 (14.840)	
- Median (Q1, Q3)	23.622 (20.252, 27.597)	45.037 (38.861, 50.527)	61.093 (58.817, 65.234)	44.466 (30.875, 55.107)	
- Min - Max	16.781 - 30.995	31.055 - 55.970	56.074 - 80.384	16.781 - 80.384	
TSOcont					0.855
- Mean (SD)	92.206 (68.051)	93.588 (67.884)	95.027 (69.335)	93.575 (68.215)	
- Median (Q1, Q3)	71.000 (47.000, 113.000)	73.000 (46.000, 117.750)	75.000 (47.750, 118.250)	73.000 (46.000, 117.500)	
- Min - Max	6.000 - 364.000	8.000 - 354.000	12.000 - 364.000	6.000 - 364.000	
TSO					0.879
- 0-45	67 (23.8%)	140 (24.7%)	63 (24.2%)	270 (24.4%)	
- 181-364	32 (11.4%)	65 (11.5%)	27 (10.4%)	124 (11.2%)	
- 46-90	112 (39.9%)	206 (36.4%)	92 (35.4%)	410 (37.0%)	
- 91-180	70 (24.9%)	155 (27.4%)	78 (30.0%)	303 (27.4%)	

Optimal Clusters' tSNE

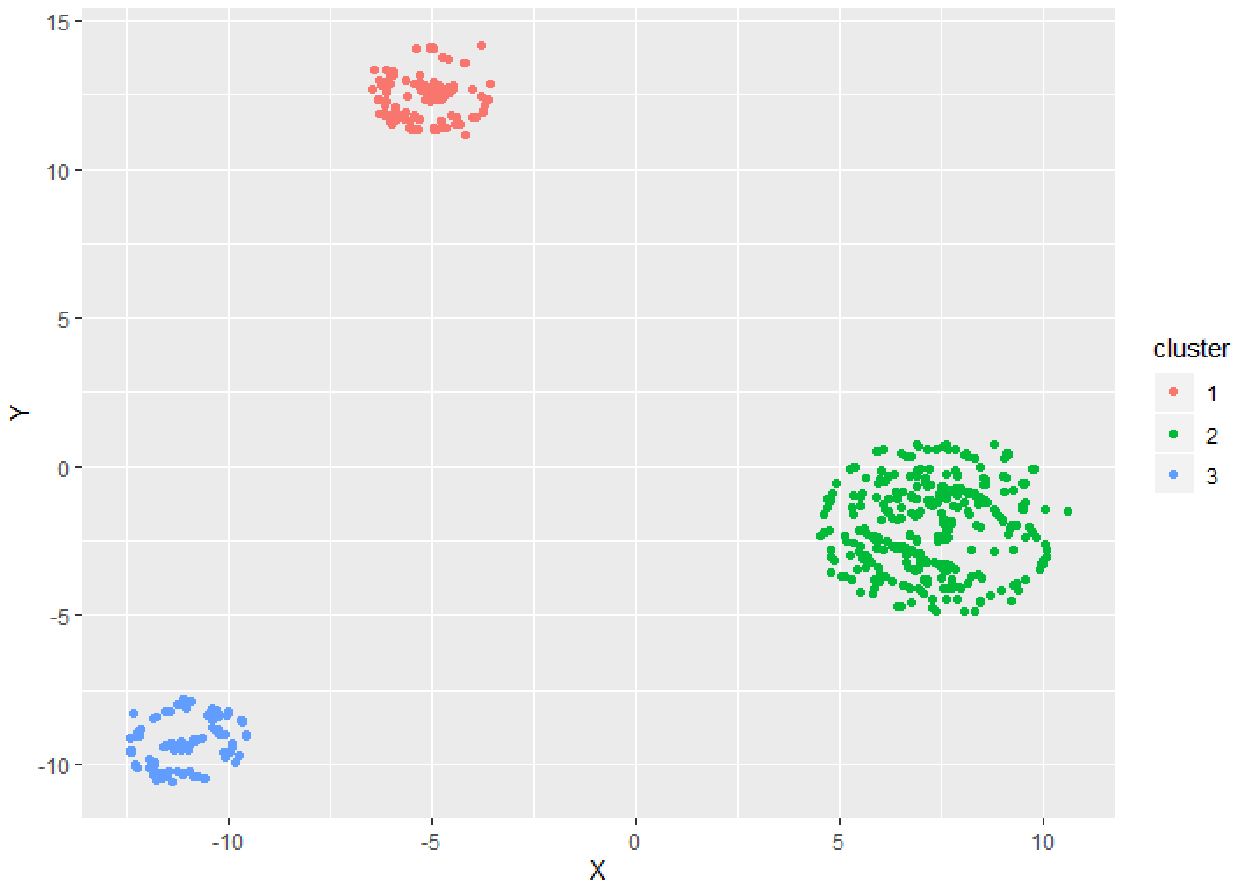

Age and time since injury

Number of clusters

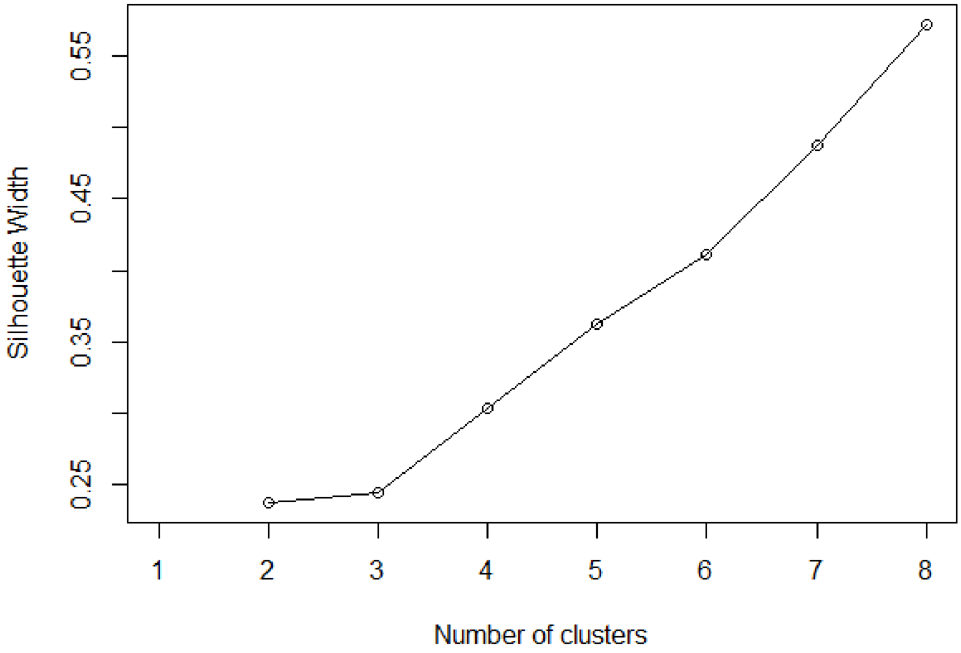

Optimal Clusters' Silhouette (k=8)

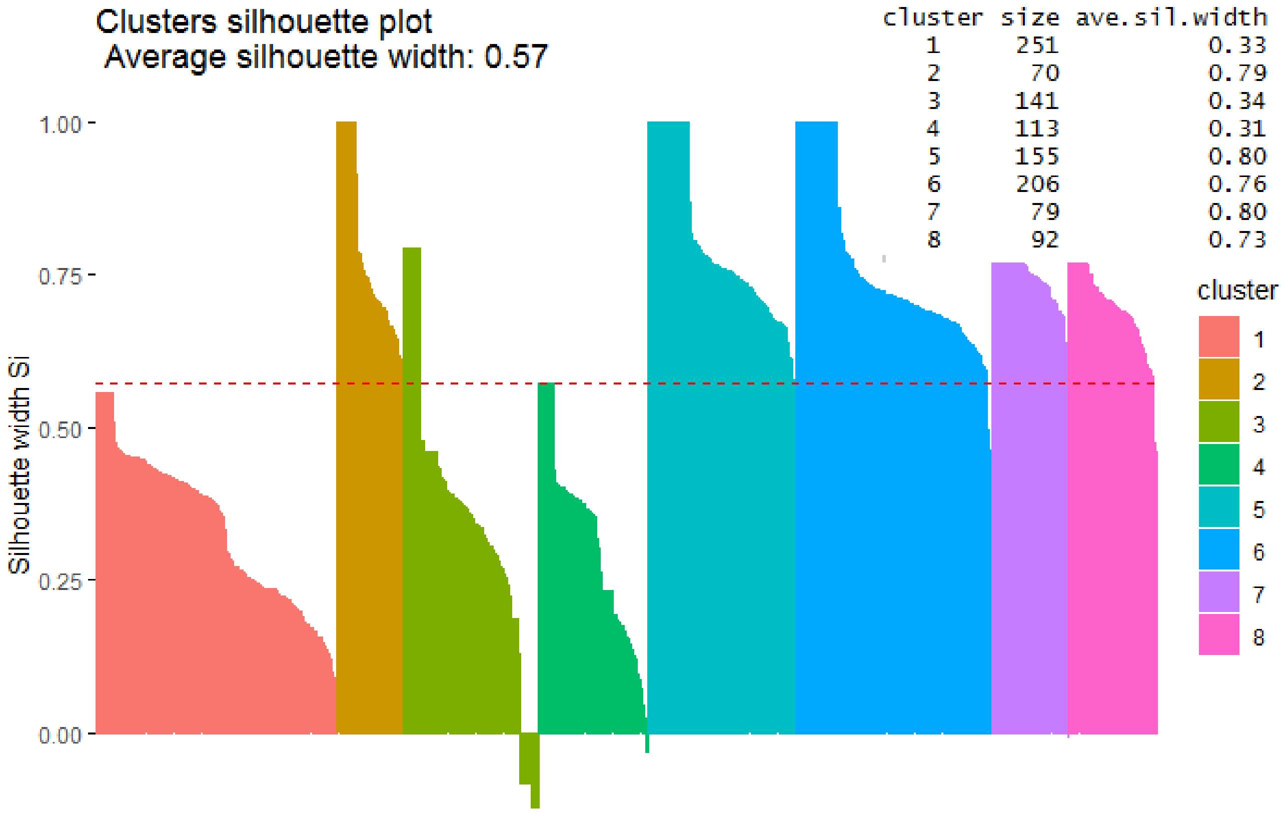

Optimal Clusters' tSNE

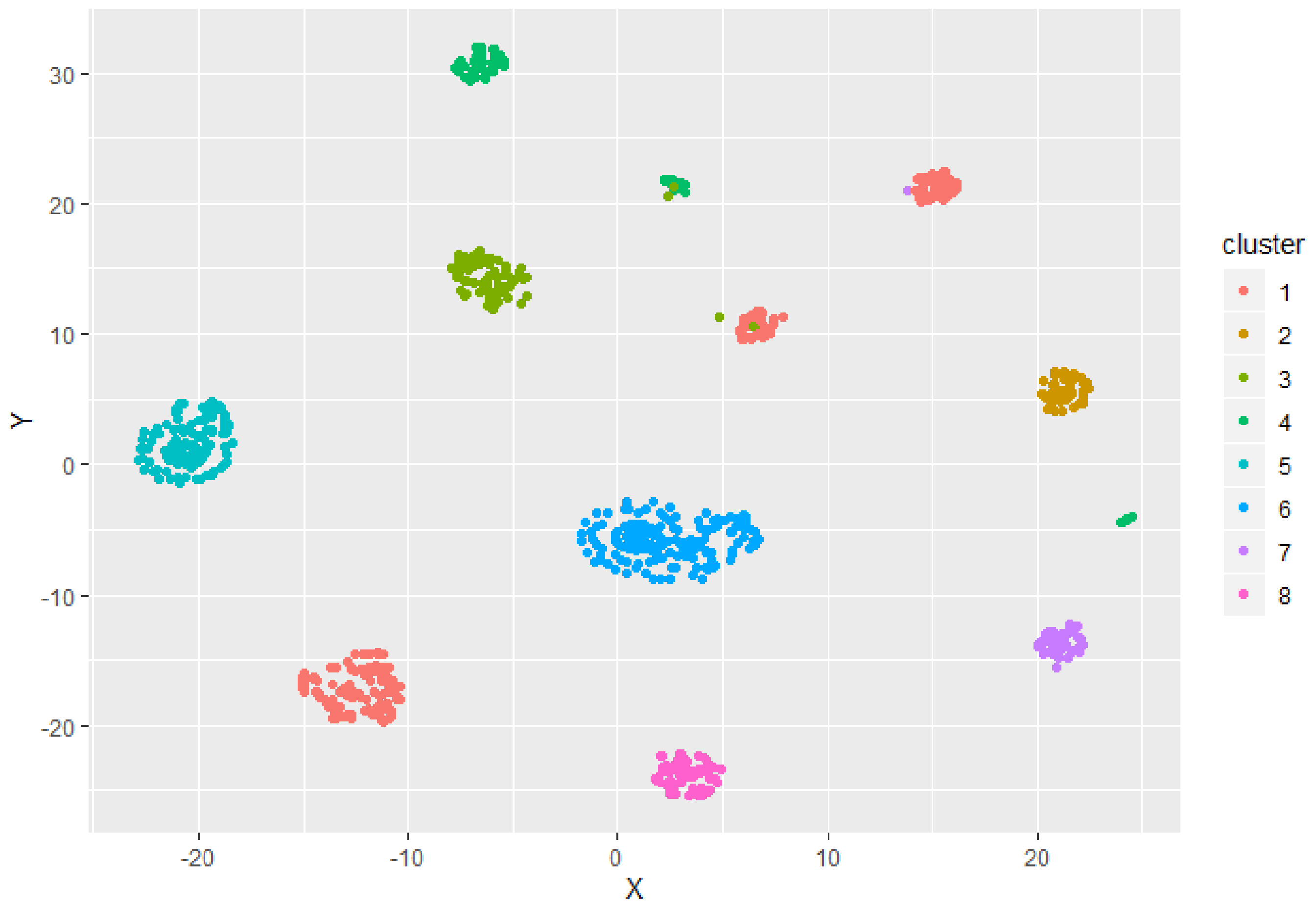

Time since injury

Only time since injury

Number of clusters

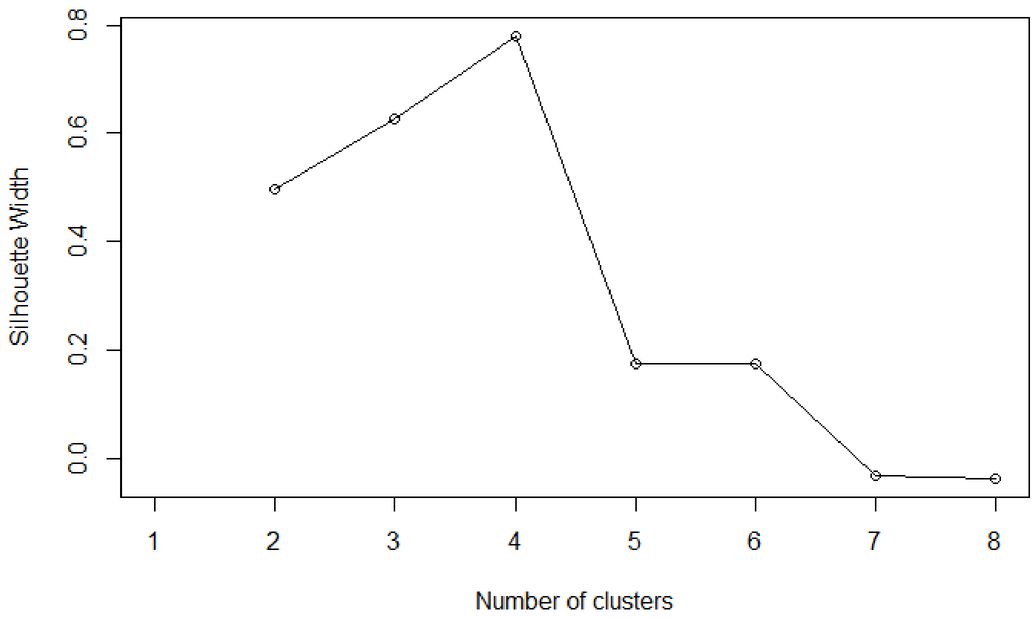

Optimal Clusters' Silhouette (k=4)

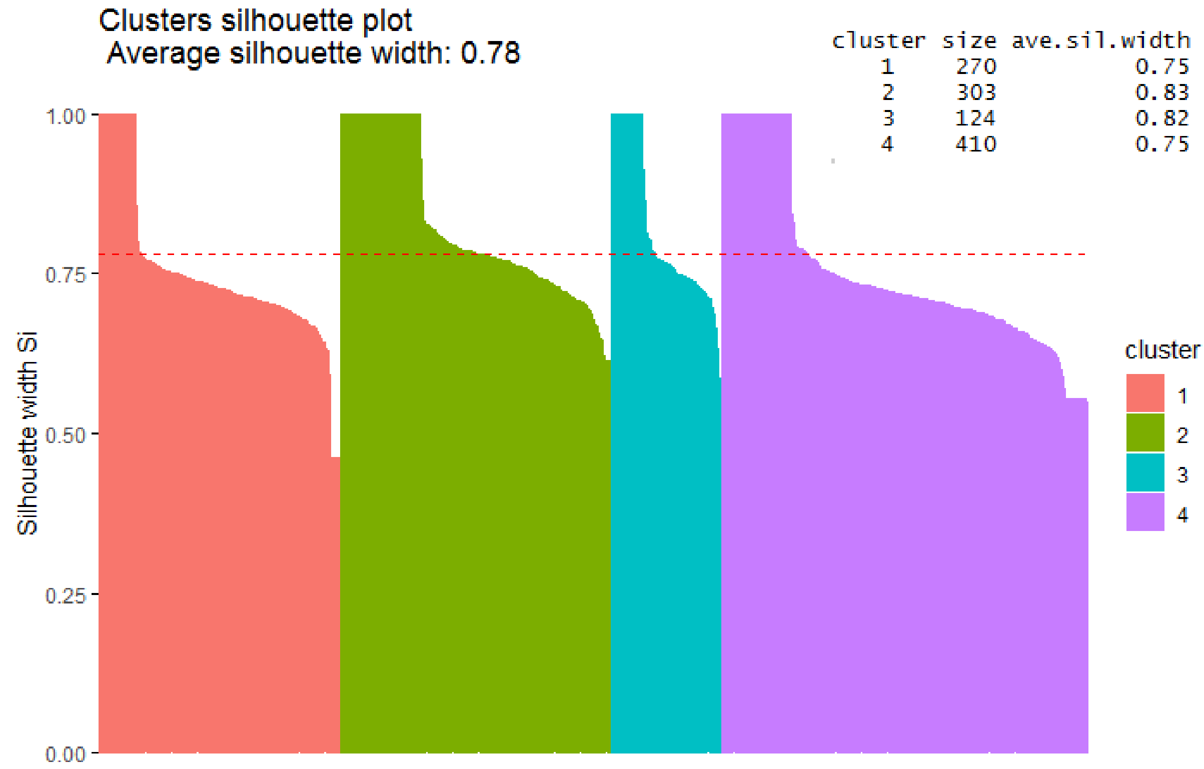

Optimal Clusters' Descriptive statistics

	1 (N=270)	2 (N=303)	3 (N=124)	4 (N=410)	Total (N=1107)	p value
T3						0.015
- Mean (SD)	20.921 (5.523)	20.165 (5.518)	19.909 (5.822)	20.108 (6.274)	20.318 (5.855)	
- Median (Q1, Q3)	23.000 (22.000, 23.000)	23.000 (21.000, 23.000)	22.500 (20.000, 23.000)	23.000 (22.000, 23.000)	23.000 (22.000, 23.000)	
- Min - Max	0.000 - 23.000	0.000 - 23.000	0.000 - 23.000	0.000 - 23.000	0.000 - 23.000	
T5						< 0.001
- Mean (SD)	84.267 (72.714)	111.348 (88.041)	95.286 (76.652)	108.076 (90.268)	100.903 (84.446)	
- Median (Q1, Q3)	60.000 (38.000, 95.000)	79.000 (51.250, 140.000)	71.500 (45.250, 114.000)	70.000 (48.000, 125.000)	68.000 (46.000, 119.000)	
- Min - Max	11.000 - 300.000	15.000 - 476.000	14.000 - 340.000	20.000 - 480.000	11.000 - 480.000	
T14						0.220
- Mean (SD)	25.136 (12.403)	23.267 (12.426)	22.206 (10.991)	24.607 (11.740)	24.217 (12.033)	
- Median (Q1, Q3)	24.000 (16.000, 33.000)	21.000 (12.000, 29.750)	20.000 (12.000, 28.500)	24.000 (16.000, 30.500)	24.000 (14.750, 31.000)	
- Min - Max	1.000 - 68.000	2.000 - 59.000	2.000 - 52.000	1.000 - 63.000	1.000 - 68.000	
T15						0.027
- Mean (SD)	3.883 (1.026)	3.601 (0.991)	3.650 (1.069)	3.799 (0.966)	3.758 (1.003)	
- Median (Q1, Q3)	4.000 (3.000, 4.000)	4.000 (3.000, 4.000)	4.000 (3.000, 4.000)	4.000 (3.000, 4.000)	4.000 (3.000, 4.000)	
- Min - Max	2.000 - 7.000	1.000 - 6.000	2.000 - 6.000	2.000 - 7.000	1.000 - 7.000	
T18						< 0.001
- Mean (SD)	5.527 (3.929)	3.655 (3.719)	4.329 (3.839)	4.174 (3.662)	4.435 (3.826)	
- Median (Q1, Q3)	6.000 (2.000, 8.000)	3.000 (0.000, 6.000)	4.000 (1.000, 7.000)	4.000 (1.000, 7.000)	4.000 (1.000, 7.000)	
- Min - Max	0.000 - 15.000	0.000 - 14.000	0.000 - 15.000	0.000 - 15.000	0.000 - 15.000	
T22						0.117
- Mean (SD)	34.450 (31.596)	42.903 (35.574)	33.636 (30.588)	48.050 (39.309)	41.490 (35.953)	
- Median (Q1, Q3)	25.000 (9.000, 44.000)	30.500 (14.750, 76.750)	25.000 (12.000, 37.000)	29.000 (13.000, 100.000)	27.000 (12.500, 70.500)	
- Min - Max	2.000 - 100.000	5.000 - 100.000	4.000 - 100.000	1.000 - 100.000	1.000 - 100.000	
type						< 0.001
- OTHERABI	39 (14.4%)	69 (22.8%)	45 (36.3%)	70 (17.1%)	223 (20.1%)	
- STROKE	91 (33.7%)	53 (17.5%)	15 (12.1%)	82 (20.0%)	241 (21.8%)	
- TBI	140 (51.9%)	181 (59.7%)	64 (51.6%)	258 (62.9%)	643 (58.1%)	
studies						0.105
- primary	118 (43.7%)	153 (50.5%)	54 (43.5%)	193 (47.1%)	518 (46.8%)	
- secondary	82 (30.4%)	93 (30.7%)	49 (39.5%)	141 (34.4%)	365 (33.0%)	
- tertiary	70 (25.9%)	57 (18.8%)	21 (16.9%)	76 (18.5%)	224 (20.2%)	
Age						0.879
- >55	63 (23.3%)	78 (25.7%)	27 (21.8%)	92 (22.4%)	260 (23.5%)	
- 17-30	67 (24.8%)	70 (23.1%)	32 (25.8%)	112 (27.3%)	281 (25.4%)	
- 31-55	140 (51.9%)	155 (51.2%)	65 (52.4%)	206 (50.2%)	566 (51.1%)	
Agecont						0.482
- Mean (SD)	43.802 (14.909)	44.536 (14.619)	43.124 (15.666)	42.783 (14.706)	43.549 (14.840)	
- Median (Q1, Q3)	45.247 (31.359, 55.023)	45.323 (33.223, 56.282)	43.662 (28.920, 54.164)	44.116 (30.111, 54.514)	44.466 (30.875, 55.107)	
- Min - Max	16.781 - 74.901	16.811 - 78.636	16.989 - 73.704	17.271 - 80.384	16.781 - 80.384	
TSOcont						< 0.001
- Mean (SD)	32.315 (8.617)	124.013 (25.424)	246.395 (52.980)	65.205 (12.724)	93.575 (68.215)	
- Median (Q1, Q3)	34.000 (26.250, 39.000)	118.000 (104.000, 142.500)	230.500 (202.000, 281.750)	65.000 (54.000, 75.000)	73.000 (46.000, 117.500)	
- Min - Max	6.000 - 45.000	91.000 - 180.000	182.000 - 364.000	46.000 - 90.000	6.000 - 364.000	
TSO						< 0.001
- 0-45	270 (100.0%)	0 (0.0%)	0 (0.0%)	0 (0.0%)	270 (24.4%)	
- 181-364	0 (0.0%)	0 (0.0%)	124 (100.0%)	0 (0.0%)	124 (11.2%)	
- 46-90	0 (0.0%)	0 (0.0%)	0 (0.0%)	410 (100.0%)	410 (37.0%)	
- 91-180	0 (0.0%)	303 (100.0%)	0 (0.0%)	0 (0.0%)	303 (27.4%)	

Optimal Clusters' tSNE

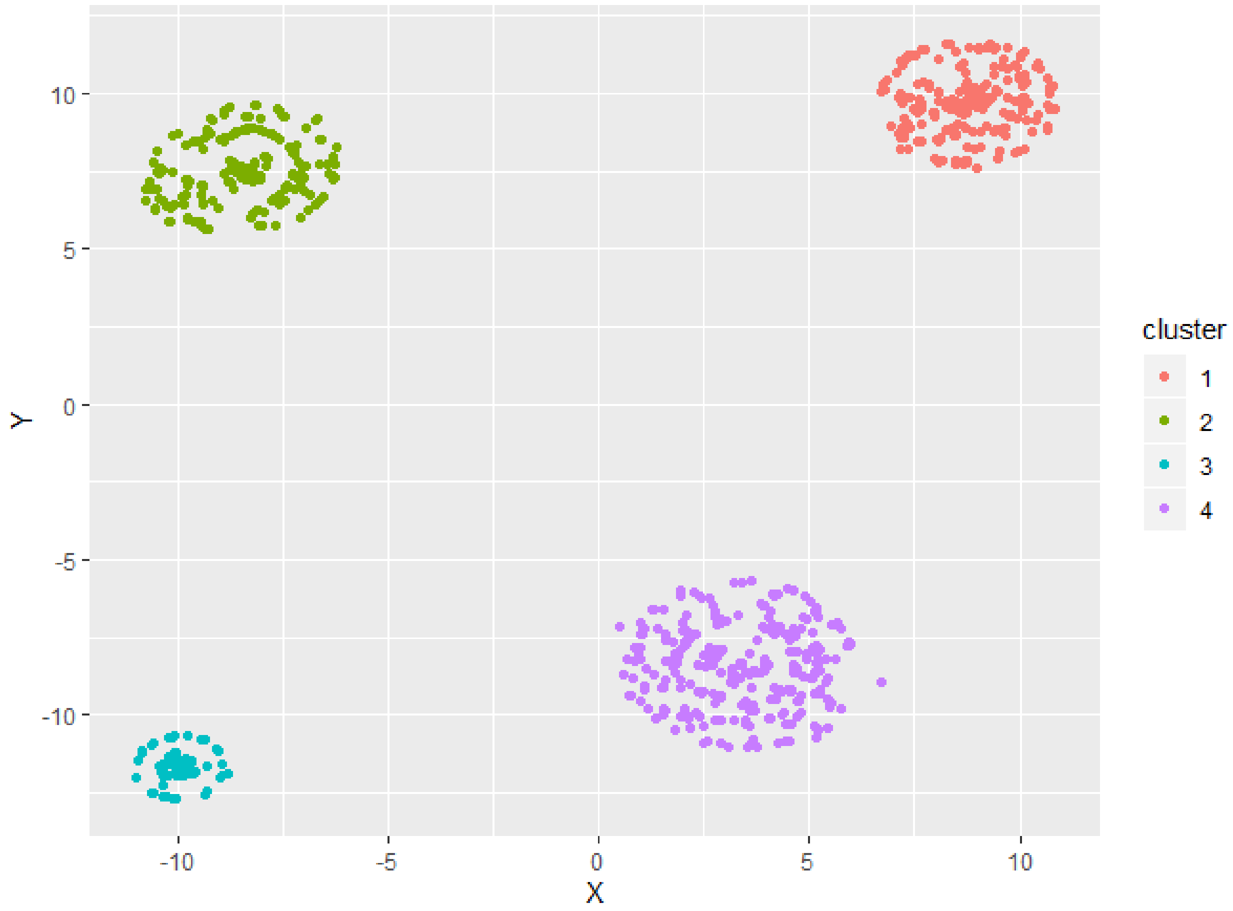

Supplement: Supplementary file 2 [file Data_Sheet_2.PDF]
